# Clinical trial registration and reporting: a survey of academic organizations in the United States

**DOI:** 10.1186/s12916-018-1042-6

**Published:** 2018-05-02

**Authors:** Evan Mayo-Wilson, James Heyward, Anthony Keyes, Jesse Reynolds, Sarah White, Nidhi Atri, Caleb Alexander, Audrey Omar, Daniel E. Ford, on behalf of the National Clinical Trials Registration and Results Reporting Taskforce Survey Subcommittee, Nidhi Atri, Hila Bernstein, Yolanda P. Davis, Keren Dunn, Carrie Dykes, James Heyward, M. E. Blair Holbein, Anthony Keyes, Evan Mayo-Wilson, Jesse Reynolds, Leah Silbert, Niem-Tzu ( Rebecca) Chen, Sarah White, Diane Lehman Wilson

**Affiliations:** 1 Department of Epidemiology, Johns Hopkins University Bloomberg School of Public Health, 615 North Wolfe Street, E6036, Baltimore, MD 21205 USA; 2 Clinical Research Projects, Johns Hopkins University School of Medicine, Baltimore, USA; 3 Yale Center for Analytical Studies, New Haven, USA; 4 Human Research Quality Improvement Program, Partners HealthCare, Boston, USA; 5 Johns Hopkins University School of Medicine, Baltimore, USA; 6 Departments of Epidemiology and Medicine, Johns Hopkins University Bloomberg School of Public Health, Baltimore, USA

**Keywords:** Clinical trials, Trial registration, Results reporting, Reporting bias

## Abstract

**Background:**

Many clinical trials conducted by academic organizations are not published, or are not published completely. Following the US Food and Drug Administration Amendments Act of 2007, “The Final Rule” (compliance date April 18, 2017) and a National Institutes of Health policy clarified and expanded trial registration and results reporting requirements. We sought to identify policies, procedures, and resources to support trial registration and reporting at academic organizations.

**Methods:**

We conducted an online survey from November 21, 2016 to March 1, 2017, before organizations were expected to comply with The Final Rule. We included active Protocol Registration and Results System (PRS) accounts classified by ClinicalTrials.gov as a “University/Organization” in the USA. PRS administrators manage information on ClinicalTrials.gov. We invited one PRS administrator to complete the survey for each organization account, which was the unit of analysis.

**Results:**

Eligible organization accounts (*N* = 783) included 47,701 records (e.g., studies) in August 2016. Participating organizations (366/783; 47%) included 40,351/47,701 (85%) records. Compared with other organizations, Clinical and Translational Science Award (CTSA) holders, cancer centers, and large organizations were more likely to participate. A minority of accounts have a registration (156/366; 43%) or results reporting policy (129/366; 35%). Of those with policies, 15/156 (11%) and 49/156 (35%) reported that trials must be registered before institutional review board approval is granted or before beginning enrollment, respectively. Few organizations use computer software to monitor compliance (68/366; 19%). One organization had penalized an investigator for non-compliance. Among the 287/366 (78%) accounts reporting that they allocate staff to fulfill ClinicalTrials.gov registration and reporting requirements, the median number of full-time equivalent staff is 0.08 (interquartile range = 0.02–0.25). Because of non-response and social desirability, this could be a “best case” scenario.

**Conclusions:**

Before the compliance date for The Final Rule, some academic organizations had policies and resources that facilitate clinical trial registration and reporting. Most organizations appear to be unprepared to meet the new requirements. Organizations could enact the following: adopt policies that require trial registration and reporting, allocate resources (e.g., staff, software) to support registration and reporting, and ensure there are consequences for investigators who do not follow standards for clinical research.

**Electronic supplementary material:**

The online version of this article (10.1186/s12916-018-1042-6) contains supplementary material, which is available to authorized users.

## Background

Clinical trials provide evidence about the safety and effectiveness of interventions (Table 1). They underpin health policy and regulation, and they inform patient and provider healthcare decision-making. Because many trials are not published [1–6], and because many published reports do not include all of the information needed to understand trial methods [7–10] and results [11–17], decisions based on published evidence alone may not lead to the best balance of benefits and harms for patients [18–21].

**Table 1:** Glossary of terms

Term	Definition
Application programming interface (API)	A set of methods to facilitate communication among software components, as described in Section 10 of the PRS User’s Guide (https://prsinfo.clinicaltrials.gov/prs-users-guide.html#section10)
Cancer center	An organization that specializes in the diagnosis and treatment of cancer, including organizations designated by the National Cancer Institute (see “National Cancer Institute cancer center”)
Clinical trial (“trial”)	A study in which human participants are assigned prospectively to receive one or more interventions (i.e., diagnostic, therapeutic, or other types) to evaluate the effects of the intervention(s) on health-related outcomes. For example, see [34, 36]
Clinical and Translational Science Awards (CTSA)	Awards funded by the National Center for Advancing Translational Sciences (NCATS), a part of the National Institutes of Health (NIH), to support a consortium of 64 medical research organizations (https://ncats.nih.gov/ctsa)
Food and Drug Administration Amendments Act of 2007 (FDAAA)	US Public Law 110-85, which established clinical trial registration and reporting requirements (section 801) [31].
Institutional review board (IRB)	A group of persons with responsibility for ensuring the protection of human subjects involved in research. For example, see [58–60]
Investigator	A researcher involved in a clinical trial [34, 36].
National Cancer Institute cancer center (NCI cancer center)	One of 69 organizations designated by the National Cancer Institute (NCI) that specialize in the diagnosis and treatment of cancer (https://www.cancer.gov/research/nci-role/cancer-centers)
Protocol Registration and Results System (PRS)	A web-based data entry system used to register studies on ClinicalTrials.gov and to submit results for registered studies
PRS organization account (“account”)	An account assigned to an organization and used to enter information about clinical trials in the Protocol Registration and Results System. An organization account may be managed by one or more administrators and may include trials conducted by multiple investigators
PRS administrator (“administrator”)	A person who manages an organization account in the Protocol Registration and Results System. Administrators are able to create accounts for individual investigators, review trial information, modify permissions for editing trial information, and check for problems
Trial registration (registration)	The process of entering a minimum dataset about a clinical trial in registry that is accessible to the public (e.g., ClinicalTrials.gov) [34, 36].
Responsible party	The person or entity responsible for submitting information about a clinical study to ClinicalTrials.gov and updating that information [34, 36].
Results	Summary information about intervention effects, including participant flow, outcome measures, and adverse events [34, 36].
Sponsor	The person or organization who oversees a clinical trial and is responsible for study data [34, 36].
The Final Rule (42 CFR 11)	A federal regulation that implements Section 801 of the Food and Drug Administration Amendments Act of 2007 (FDAAA) and expands requirements for trial registration and results reporting. The effective date is January 18, 2017 and the compliance date is April 18, 2017 [34].
University/organization	A “type of organization” used to classify PRS organization accounts by www.ClinicalTrials.gov.

To help participants enroll in trials, improve access to information, and reduce bias, authors have long proposed registering all trials prospectively [22–27]. The Food and Drug Administration Modernization Act of 1997 led to the creation of ClinicalTrials.gov, a publicly accessible database maintained by the National Library of Medicine (NLM), which launched in 2000 [28]. In 2004, the International Committee of Medical Journal Editors (ICMJE) announced that trials initiated from 2005 would have to be registered to be considered for publication [29, 30]. Title VIII of the Food and Drug Administration Amendments Act of 2007 (FDAAA) [31] required that certain trials of drugs, biologics, and medical devices be registered and that results for trials of approved products be posted on ClinicalTrials.gov. The FDAAA also authorized the Food and Drug Administration (FDA) to issue fines for non-compliance, currently up to $11,569 per trial per day [32]. “The Final Rule,”, which took effect on January 18, 2017, clarified and expanded requirements for registration and reporting (Box 1) [33, 34]; organizations were expected to be in compliance by April 18, 2017. In a complementary policy, the National Institutes of Health (NIH) issued broader requirements that apply to *all* trials funded by the NIH, including early trials and trials of behavioral interventions [35, 36].

There is little evidence about how academic organizations support trial registration and reporting, but some evidence suggests that they are unprepared to meet these requirements. For example, academic organizations have performed worse than industry in registering trials prospectively [37, 38] and reporting results [39–46].

## Methods

Between November 21, 2016 and March 1, 2017, we conducted an online survey of academic organizations in the USA. We surveyed administrators who are responsible for maintaining organization accounts on ClinicalTrials.gov. For each eligible ClinicalTrials.gov account, we asked one administrator to describe the policies and procedures and the available resources to support trial registration and reporting at their organization (Box 2).

### Identifying eligible PRS accounts

The online system used to enter information in the ClinicalTrials.gov database is called the Protocol Registration and Results System (PRS). Each study registered on ClinicalTrials.gov is associated with a “record” of that study, and each record is assigned to one PRS organization account. A record may or may not include study results. A single organization, such as a university or health system, might register trials using one or many accounts. For example, “Yale University” is one account; by comparison, “Harvard Medical School” and “Harvard School of Dental Medicine” are each separate accounts.

We used the PRS account as the unit of analysis because accounts related to the same organization often represent schools or departments that have separate policies and procedures related to trial registration and reporting. Furthermore, we are not aware of a reliable method to associate individual accounts with organization. For example, the “Johns Hopkins University” account includes mostly records from the Johns Hopkins University School of Medicine. Investigators at Johns Hopkins University also register trials using the accounts “Johns Hopkins Bloomberg School of Public Health,” “Johns Hopkins Children’s Hospital,” and “Sidney Kimmel Comprehensive Cancer Center.” Schools and hospitals related to Johns Hopkins University have distinct policies, faculties, administrative staff, and institutional review boards (IRBs).

We included all “active” accounts categorized by ClinicalTrials.gov as a “University/Organization” in the USA. We received a spreadsheet from the NLM with the number of records in each eligible account on August 4, 2016, and we received PRS administrator contact information from the NLM on September 28, 2016 and December 12, 2016.

### Survey design

We developed a draft survey based on investigators’ content knowledge and evidence from studies that were known to us at the time. We organized questions into three domains: (1) organization characteristics, (2) registration and results policies and practices, and (3) staff and resources. We also invited participants to describe any compliance efforts that our questions did not cover. We then piloted the survey among 14 members of the National Clinical Trials Registration and Results Reporting Taskforce. The final survey used skip logic so that participants saw only those questions that were relevant based on their previous answers. Responses were saved automatically, and participants could return to the survey at any time; this allowed participants to discuss their answers with organizational colleagues before submitting. We conducted the survey using Qualtrics software (www.qualtrics.com/); a copy is available as a Word document (Additional file 1) and on the Qualtrics website (http://bit.ly/2tCSqyl).

### Participant recruitment

One or more persons, called “PRS administrators” by ClinicalTrials.gov, may add or modify records in each account. Some PRS administrators are employed specifically to work on ClinicalTrials.gov, but many PRS administrators have little or no time budgeted by their organizations to work on ClinicalTrials.gov.

For each eligible account, we created a unique internet address (URL) which we emailed in an invitation letter to one administrator. For accounts with more than one administrator, we first contacted all administrators and asked them to select the appropriate administrator to complete this survey; then, we sent the survey to the nominated administrator. If an administrator did not complete the survey, EMW sent at least two reminders from his university email account after approximately 2 weeks and 4 weeks. For accounts with multiple administrators, we emailed all administrators if the designated administrator did not respond after two reminders. We instructed administrators associated with multiple accounts to complete a separate survey for each account.

Participants indicated their consent by continuing past the opening page and by completing the survey.

### Analyses

To analyze the results, we first excluded accounts that did not complete three required questions about whether they had: (1) a registration policy, (2) a results reporting policy, and (3) computer software to manage their records. We then calculated descriptive statistics using SPSS 24. For categorical data, we calculated the number and proportion of accounts that selected each response. For continuous data, we calculated the median and interquartile range (IQR) depending on the distribution of responses.

We conducted subgroup analyses to determine whether organization characteristics might be related to policies and resources. We compared:

1. Accounts affiliated with a Clinical and Translational Science Award (CTSA) versus other accounts
2. Accounts affiliated with a cancer center versus other accounts
3. Accounts with < 20 records, 20–99 records, and ≥ 100 records

We conducted a sensitivity analysis to determine whether the results might be sensitive to non-response bias by comparing accounts that responded before the effective date for The Final Rule (January 18, 2017) with accounts that responded on or after The Final Rule took effect.

## Results

### Characteristics of eligible accounts

We identified 783 eligible accounts (Additional file 2), which had 47,701 records by August 2016. The median number of records per account was 7 (IQR = 3–36), ranging from 1 (two accounts) to 1563 (mean = 61, standard deviation (SD) = 155). A minority of accounts are responsible for most records; 113/783 (14%) accounts had ≥ 100 records by August 2016, and these accounts were responsible for 38,311/47,701 (80%) records.

The median number of administrators per account was 1 (IQR = 1–3), and one organization had 182 registered administrators.

### Survey participation

**Fig. 1:**
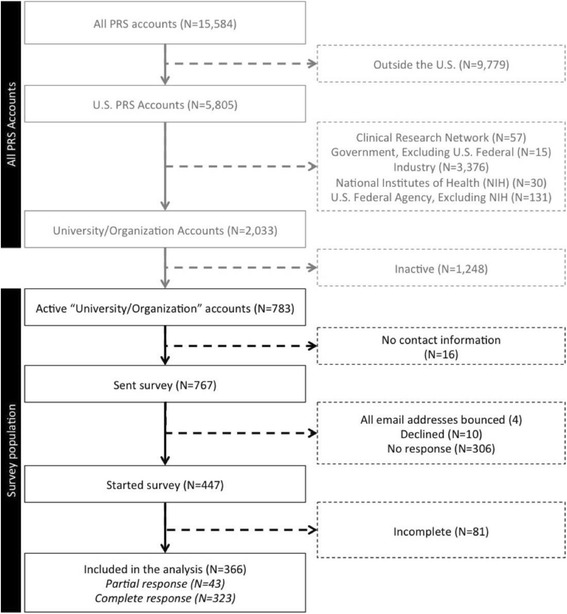
Flowchart: PRS accounts included in the survey

Of 783 eligible accounts, we found no contact details for 16 (2%) and attempted to contact 767 (98%). In four cases (< 1%), we were unable to identify a usable email address. Of eligible accounts, 10/783 (1%) emailed us to decline, 306/783 (39%) did not participate in the survey, and 81/783 (10%) did not provide sufficient information to be included in the analysis (Fig. 1). Two accounts reported that they had multiple policies related to the same account; we asked them to complete questions about their account characteristics but not to complete questions about their specific policies and resources.

Included accounts were responsible for 40,351/47,701 (85%) records registered by eligible accounts. We received a partial (43) or complete (323) survey for 366/783 (47%) eligible accounts (Additional file 3).

The first account completed the survey on November 21, 2016, and the last account completed the survey on March 21, 2017; 31/366 (9%) accounts completed the survey after January 17, 2017. Because of skip logic and because some accounts did not answer all possible questions, accounts answered between 6 and 42 questions (median 19, IQR 17–29).

### Policies and practices

Of 366 accounts, 156 (43%) reported that they have a registration policy and 129 (35%) have a results reporting policy (Table 2). Policies came into effect between 2000 and 2016 (median = 2013, IQR 2010–2015; mode = 2016).

**Table 2:** Clinical trial registration and results reporting policies

QUESTION (number of participants who viewed the question)	No.	Percentage
Trial registration policies		
Does the organization have a registration policy? (*N* = 366)^a^		
Yes	156	43%
No	173	47%
Don’t know	37	10%
Does the policy cover investigators joining the organization? (*N* = 156)^b^		
Yes	57	37%
No	76	49%
Don’t know	23	15%
Skipped (did not answer)	0	0%
Does the policy cover investigators leaving the organization? (N = 156)^b^		
Yes	38	24%
No	87	56%
Don’t know	31	20%
Skipped (did not answer)	0	0%
According to the policy, when must trials be registered? (*N* = 139)^b,c^		
Before IRB approval	15	11%
Before enrollment begins	49	35%
Within 21 days of beginning enrollment	31	22%
Requirements differ among trials	21	15%
This is not addressed in the policy	18	13%
Don’t know	4	3%
Skipped (did not answer)	1	1%
According to the policy, who is responsible for determining whether a trial must be registered? (*N* = 129)^b,c^		
Principal investigator	72	56%
Institutional review board	20	16%
PRS administrator	35	27%
Other	14	11%
This responsibility is not assigned in the policy	12	9%
Don’t know	0	0%
Skipped (did not answer)	0	0%
According to the policy, can investigators be penalized by the organization for failing to register a trial? (*N* = 139)^b^		
Yes	27	23%
No	91	91%
Don’t know	21	21%
Skipped (did not answer)	0	0%
Date the trial registration policy came into effect (*N* = 139)^b,d^		
Minimum (year)	2000	
Maximum (year)	2016	
Median (year)	2013	
Results reporting policies		
Does the organization have a results reporting policy? (N = 366)^a^		
Yes	129	35%
No	193	53%
Don’t know	44	12%
According to the policy, who is responsible for monitoring if results are reported on time? (*N* = 115)^b,c^		
Principal investigator	54	47%
Institutional review board	5	4%
PRS administrator	68	59%
Other	12	10%
This responsibility is not assigned in the policy	18	16%
Don’t know	1	1%
Skipped (did not answer)	0	0%
According to the policy, can investigators be penalized by the organization for failing to report a trial (*N* = 114)^e^		
Yes	21	18%
No	75	66%
Don’t know	18	16%
Skipped (did not answer)	0	0%

^a^An answer to this question was required for an account to be included in the analysis; accounts that did not see or skipped this question were excluded from all analyses

^b^The number of possible responses (i.e., the denominator) includes the accounts with a relevant policy that viewed this question. The number of accounts that viewed each question is less than the total number of accounts in the study because (1) participants did not see all questions because of skip logic, and (2) some participants discontinued the survey before viewing all questions

^c^Because participants could “check all that apply,” the sum of all categories exceeds the number of participants who responded (i.e., some participants selected multiple responses)

^d^Because 50 (36%) selected “Don’t know,” 89 accounts are included in the analysis

^e^Of 111 accounts who viewed either question about penalties for (1) failing to register or (2) failing to report a trial, 17 (15%) responded “Yes” to both questions, and 31 (28%) responded “Yes” to one or both questions

Among those accounts with policies, most policies require registration of trials applicable under FDAAA (118/140, 84%) and funded by the NIH (72/140, 51%) (Additional file 4). Polices include different requirements for time of registration (Table 3); most require that trials be registered before IRB approval is granted (15/156; 11%), before enrollment begins (49/156; 35%), or within 21 days of beginning enrollment (31/156; 22%). A minority of policies address handling trials associated with investigators joining (57/156; 37%) and leaving organizations (38/156; 24%).

**Table 3:** Resources to support clinical trial registration and results reporting

QUESTION (number of participants who viewed question)	No.	Percentage
Does the organization have an electronic system for managing trial registration or results reporting? (*N* = 366)^a^		
Yes^b^	68	19%
No	272	74%
Don’t know	26	7%
Which functions do staff who support registration and results reporting perform (*N* = 342)^c^		
Group training (e.g., classroom style)	61	18%
Individual training	151	44%
Enter data for principal investigators (PIs)	174	51%
Maintain an educational website	57	17%
Notify PIs about problems or sanctions	241	70%
Assistance with analysis	58	17%
Respond to questions	241	70%
Review problem records	262	77%
Other	28	8%
Don’t know	22	6%
Skipped (did not answer)	0	0%
What is the highest qualification of any staff member? (*N* = 315)^d^		
High school	11	3%
Bachelors	68	22%
Masters	123	39%
Higher degree^d^	109	35%
Skipped (did not answer)	4	1%
Does the organization monitor compliance with results reporting requirements? (*N* = 116)		
Yes	99	85%
No	10	9%
Don’t know	7	6%
Skipped (did not answer)	0	0%
Who monitors compliance with results reporting requirements? (*N* = 99)^c,f^		
PRS administrator	85	86%
Institutional review board (IRB)^g^	11	11%
Other	20	20%
Don’t know	0	0%
Skipped (did not answer)	0	0%
Number of full-time equivalent (FTE) staff (*N* = 287)^h^	Median = 0.08	IQR = 0.02 to 0.25

^a^An answer to this question was required for an account to be included in the analysis; accounts that did not see or skipped this question were excluded from all analyses

^b^Of the 68 accounts that use an electronic management system (“computer software”), 2 (3%) use an application programming interface (API) to communicate with ClinicalTrials.gov

^c^Because participants could “check all that apply,” the sum of all categories exceeds the number of participants who responded (i.e., some participants selected multiple responses)

^d^The number of possible responses (i.e., the denominator) includes the accounts with a relevant policy that viewed this question. The number of accounts that viewed each question is less than the total number of accounts in the study because (1) participants did not see all questions because of skip logic, and (2) some participants discontinued the survey before viewing all questions

^e^Higher degrees include JD (*N* = 21, 7%), PhD (*N* = 69, 22%), and MD (*N* = 32, 10%); 13 accounts selected 2 higher degrees (8 both PhD and JD, 5 both PhD and MD)

^f^The number of possible responses was limited to the accounts that reported monitoring compliance with their results reporting policy

^g^Of the 11 accounts reporting that IRBs monitor trial registration, 4 indicated that the IRB requires registration for approval for some (*N* = 3) or all trials (*N* = 1)

^h^Results are the median and interquartile range. We also calculated mean = 0.3, standard deviation = 0.6

Responsibility for registering trials is most often assigned to principal investigators (72/129; 56%). Responsibility for monitoring whether results are reported on time is most often assigned to principal investigators (54/115, 47%) and administrators (68/115, 59%).

Some policies allow organizations to penalize investigators who fail to register trials (27/115; 18%) or fail to report results (21/114; 18%). One account (< 1%) reported that their organization had penalized an investigator for non-compliance.

### Resources

Few accounts use computer software to manage their records (68/366; 19%). Of those that use computer software, two use the application programming interface (API) to connect with ClinicalTrials.gov (Table 3).

Among the 287/366 (78%) accounts that allocate staff to fulfill ClinicalTrials.gov registration and reporting requirements, the median number of full-time equivalent (FTE) staff is 0.08 (IQR = 0.02–0.25). Among the staff who support ClinicalTrials.gov registration and reporting requirements, the staff member with the highest level of education has a graduate degree (232/411; 75%) more often than a bachelor’s degree (68/411; 22%) or a high school diploma (11/411; 3%). At the time of this survey, 34/338 (10%) planned to hire more staff, while 217/338 (64%) and 87/338 (26%) did not plan to hire more staff or did not know, respectively. Among accounts affiliated with a CTSA, 24/109 (22%) receive support for ClinicalTrials.gov compliance from the CTSA.

Staff perform various roles, including educating investigators individually (151/342; 44%) and in groups (61/42; 18%), entering data for principal investigators (174/342; 51%), maintaining educational websites (57/342; 17%), notifying investigators about problems (241/342; 70%), assisting with analysis (58/342; 17%), responding to questions (241/342; 70%), and reviewing problem records (262/342; 77%).

### Subgroup analyses

Registration and reporting policies are more common among the following accounts: (1) those with many records, (2) those affiliated with CTSAs, and (3) those affiliated with cancer centers (Table 4). For example, most cancer centers have a registration policy (61/97; 63%) and a reporting policy (52/97; 54%); a minority of other accounts have a registration policy (94/267; 35%) or a reporting policy (77/267; 28%).

**Table 4:** Subgroup analysis

	CTSA affiliation				Cancer center affiliation^a^				Account size						Total	
QUESTION (number of participants who viewed question)	CTSA (*N* = 109)		Not CTSA (*N* = 257)		Cancer center (*N* = 97)		Not cancer center (*N* = 267)		≥ 100 records (*N* = 98)		20–99 records (*N* = 77)		< 20 reords (*N* = 191)		(N = 366)	
Number of records	29,076		11,275		24,970		14,940		35,269		3756		1326		40,351	
Does the organization have a registration policy? (*N* = 366)																
Yes	58	53%	98	38%	61	63%	94	35%	63	64%	33	43%	60	31%	156	43%
No	40	37%	133	52%	30	31%	142	53%	29	30%	39	51%	105	55%	173	47%
Don’t know	11	10%	26	10%	6	6%	31	12%	6	6%	5	6%	26	14%	37	10%
Does the organization have a results reporting policy? (*N* = 366)																
Yes	52	48%	77	30%	52	54%	77	28%	55	56%	26	34%	48	25%	129	35%
No	46	42%	147	57%	37	38%	154	58%	36	37%	44	57%	113	59%	193	53%
Don’t know	11	10%	33	13%	8	8%	36	14%	7	7%	7	9%	30	16%	44	12%
Does the organization have an electronic system for managing trial registration or results reporting? (*N* = 366)																
Yes	27	25%	41	16%	26	27%	41	15%	26	27%	7	9%	35	18%	68	19%
No	73	67%	199	77%	68	70%	203	76%	68	69%	66	86%	138	72%	272	74%
Don’t know	9	8%	17	7%	3	3%	23	9%	18	18%	4	5%	4	2%	26	7%
Number of full-time equivalent (FTE) staff (*N* = 288)^b^	*N* = 94 Mean (SD) = 0.59 (0.83)		*N* = 193 Mean (SD) = 0. 17 (0.39)		*N* = 84 Mean (SD) = 0.56 (0.81)		*N* = 201 Mean (SD) = 0.25 (0.72)		*N* = 88 Mean (SD) = 0.69 (0.83)		*N* = 61 Mean (SD) = 0.13 (0.24)		*N* = 138 Mean (SD) = 0.14 (0.41)		N = 287 Mean (SD) = 0.31 (0.60)	
	Median (IQR) = 0.25 (0.05 to 0.95)		Median (IQR) = 0.05 (0.02 to 0.15)		Median (IQR) = 0.25 (0.05 to 0.85)		Median (IQR) = 0.05 (0.02 to 0.15)		Median (IQR) = 0.42 (0.15 to 1.00)		Median (IQR) = 0.06 (0.02 to 0.15)		Median (IQR) = 0.05 (0.01 to 0.10)		Median (IQR) = 0.08 (0.02 to 0.25)	
	Range 0 to 5.6		Range 0 to 4		Range 0 to 5.6		Range 0 to 4		Range 0 to 5.6		Range 0 to 1.58		Range 0 to 4		Range 0 to 5.6	

*CTSA* Accounts affiliated with a Clinical and Translational Science Award (CTSA). The number of CTSA affiliated accounts exceeds the number of CTSAs because multiple accounts were sometimes affiliated with the same CTSA. *Not CTSA* Accounts not affiliated with a CTSA. *Cancer center* Accounts affiliated with a National Cancer Institute (NCI) cancer center or another cancer center. *Not cancer center* Accounts not affiliated with an NCI or other cancer center. *≥ 100 records* Accounts with 100 or more registered studies in the USA for which the organization was listed as the “lead sponsor.” *20–99 records* Accounts with between 20 and 99 registered studies. *< 20 records* Accounts with fewer than 20 registered studies

^a^Two accounts did not report whether they are affiliated with a cancer center; they are not included in this subgroup analysis

^b^Results are for accounts that responded to this question. In our initial analysis, we found potentially invalid data; for example, some participants entered “0.5” rather than “50%”. This occurred because a software bug prevented us from enforcing a data validation rule in the survey. To verify these results, we emailed administrators who indicated that staff spent ≤ 1% of their time on trial registration and reporting. Post hoc, we excluded two outliers because they appeared to report the total number of staff employed at the organization rather than the number of staff who support trial registration and results reporting

### Non-response bias

We found direct and indirect evidence of non-response bias, which suggests that our results might overestimate the amount of support available at academic organizations. For example, one administrator who declined to participate replied that their organization “does not have any central staff managing clinicaltrials.gov and does not utilize an institutional account.”

Account size was related to survey participation, and many participating accounts were large (Table 5). Of those accounts we invited to complete the survey that included < 20 records, 171/532 (32%) participated. By comparison, 98/113 (87%) accounts with ≥ 100 records participated.

**Table 5:** Characteristics of participants

QUESTION (number of participants who viewed the question)	No.	Percentage
Eligible accounts (*N* = 783)^a^		
< 20 records^b^	532	68%
20–99 records	138	18%
≥ 100 records	113	14%
Participants (*N* = 366)		
< 20 records	191	52%
20–99 records	77	21%
≥ 100 records	98	27%
Is the PRS account affiliated with a CTSA? (*N* = 366)		
Yes (selected a CTSA)	109	30%
No (selected “Not applicable”)	211	58%
Skipped (did not answer)	46	13%
Is the PRS account affiliated with one or more of the following? (*N* = 366)^a^		
NCI cancer center	62	17%
Other cancer center (not NCI designated)^c^	37	10%
Medical school	100	27%
Teaching hospital	138	38%
Other schools^d^	107	29%
Other	172	47%
Selected “Don’t know”	19	5%
Skipped (did not answer)	2	< 1%

^a^Because participants could “check all that apply,” the sum of all categories exceeds the number of accounts that participated (i.e., some participants selected multiple responses)

^b^Records include studies for which the organization was listed as the “lead sponsor” and the study was conducted in the USA; that is, we excluded records for which the principal investigator (PI) was the “lead sponsor,” and we excluded studies done outside the USA

^c^Two accounts selected both an “NCI cancer center” and an “Other cancer center”; thus, 97 accounts were affiliated with a cancer center

^d^ “Other schools” include: school of public health (*N* = 59, 16%), school of social work (*N* = 41, 11%), school of arts and sciences (*N* = 56, 15%), school of nursing (*N* = 72, 20%), school of dentistry (*N* = 40, 11%)

Participation might have been related to organization resources. Nearly all CTSAs (62/64; 97%) and most National Cancer Institute (NCI) cancer centers (55/69; 80%) participated in the survey (Table 5), including 48 accounts affiliated with both a cancer center and a CTSA. Furthermore, some included accounts were related; for example, 107 accounts were affiliated with one of the 62 CTSAs.

In a sensitivity analysis (Additional file 5), we found no clear differences in policies and computer software when comparing early and late responders. Most participants completed the survey before the effective date, so late responders included only 31/366 (8%) accounts.

## Discussion

### Summary of findings

To our knowledge, this is the largest and most comprehensive survey of organizations that register and report clinical trials on ClinicalTrials.gov. We had a high participation rate, and accounts that completed the survey conduct the overwhelming majority of clinical trials registered by academic organizations in the USA. We found that some organizations were prepared to meet trial registration and reporting requirements before The Final Rule took effect, but there is wide variation in practice. Most organizations do not have policies for trial registration and reporting. Most existing policies are consistent with FDAAA; however, most are not consistent with the ICMJE registration policy. Nearly half of existing policies do not require registration of all NIH-funded trials, though organizations could adapt their polices in response to the new NIH requirements. Few policies include penalties for investigators who do not register or report their trials. Although some organizations use computer software to monitor trial registration and reporting, only two have systems that connect directly with ClinicalTrials.gov (i.e., using API). Most staff who support trial registration and reporting have other responsibilities, and most organizations do not plan to hire more staff to support trial registration and reporting.

### Implications

Our results suggest that most organizations assign responsibility for trial registration and reporting to individual investigators and provide little oversight. Previous studies indicate that senior investigators often delegate this responsibility to their junior colleagues [47].

To our knowledge, the FDA has never assessed a civil monetary penalty for failing to register or report a trial, and the NIH has never penalized an organization for failing to meet their requirements. The ICMJE policy is not applied uniformly [48], and many published trials are still not registered prospectively and completely [37, 49–52]. Organizations may be more likely to comply with these requirements if they are held accountable for doing so by journals, FDA, and funders (see, e.g., http://www.who.int/ictrp/results/jointstatement/en).

Improving research transparency in the long term will require changes in norms and culture. Organizations could take four immediate steps to improve trial registration and reporting. First, organizations could offer education to help investigators understand these requirements. Second, organizations could implement policies and procedures to support trial registration and reporting. For example, organizations could require that investigators answer questions on IRB applications to identify clinical trials that require registration. Organizations could also require that investigators provide trial registration numbers before allowing trials to commence. Third, organizations could identify trials that do not meet trial registration and reporting requirements and help individual investigators bring those trials into compliance. Notably, software could provide automatic reminders when trial information needs to be updated [53] or when results will be due, and software could help organizations identify problems that require attention from leaders. Prospective reminders would allow administrators and investigators to update information before they become non-compliant with reporting requirements. Finally, organizations could ensure there are consequences for investigators who fail to meet trial registration and reporting requirements. For example, organizations could stop enrollment in ongoing trials or stop investigators from obtaining new grants [54].

### Limitations

Although we sent multiple reminders and gave participants months to respond, our results might be influenced by non-response and social desirability. However, such biases would lead us to overestimate support for research trial registration and reporting. Participating accounts conduct more trials than non-participating accounts, and they appear to be most likely to have policies and resources to support transparency.

Because we analyzed results by account, our results are not directly comparable with studies that grouped trials using the data fields “funder” [39, 40, 43], “sponsor” [41, 44], “collaborator” [41], or “affiliation” [42]. We analyzed results by account because (1) the account should usually represent the “responsible party,” which is the person or organization legally responsible for fulfilling trial registration and reporting requirements, and (2) because we were not aware of another method to identify all trials, or even all accounts, associated with each organization.

We could not always determine which trials were associated with specific organizations, and organizations might not know which accounts their investigators use. Organizations could work with ClincalTrials.gov to identify non-working email addresses, update administrators’ contact information, assign and identify an administrator responsible for overseeing each account, and create a one-to-one relationship between each account and organization. For example, ClinicalTrials.gov could identify multiple accounts managed by administrators at the same organization and help organizations move information into a single account. Organizations would need to prepare before centralizing their records; centralized administration could reduce trial registration and reporting if administrators lack the time, training, and resources to manage these tasks effectively.

We requested information from one administrator at each organization, and administrators might have been unaware of policies and practices that affect other parts of their organizations (e.g., IRBs, grant management). Finally, some organizations were misclassified on ClinicalTrials.gov (e.g., non-US organizations); we do not know how many organizations were inadvertently included or excluded because of misclassification.

### Future research

Further research is needed to determine how to support trial registration and reporting at different types of organizations. Some large organizations register several trials each week, while other organizations register a few trials each year. For small organizations, hiring staff to support trial registration and reporting could be prohibitively expensive. Further qualitative research could explore how different types of organizations are responding to these requirements.

Future surveys could examine predictors of compliance with trial registration and reporting requirements. Although there are important variations in policy and practice, additional quantitative analyses would have little immediate value because most organizations have low compliance [37–45]. Instead, detailed case studies might be most useful for identifying best practices. For example, Duke Medicine developed a centralized approach [55], and the US Department of Veterans Affairs (VA) described multiple efforts to support transparency, including an “internal web-based portal system” [54]. The National Clinical Trials Registration and Results Reporting Taskforce is a network of administrators who meet monthly by teleconference, share resources (e.g., educational materials), and provide informal peer education. As industry appears to be doing better than academia [37, 39–44], it might be useful for academic organizations to understand the methods industry uses to monitor and report compliance (see, e.g., [56]).

We surveyed organizations after the publication of The Final Rule, and most accounts completed the survey before The Final Rule took effect, several months before the compliance date [34]. Our results should be considered a “baseline” for future studies investigating whether organizations adopt new policies and procedures, and whether they allocate new resources, to fulfill registration and reporting requirements. The federal government estimates compliance costs for organizations will be $70,287,277 per year [34]. This survey, and future updates, could be used to improve estimates of the costs of compliance.

## Conclusions

To support clinical trial registration and results reporting, organizations should strongly consider adopting appropriate policies, allocating resources to implement those policies, and ensuring there are consequences for investigators who do not register and report the results of their research.

## Box 1: Registration and reporting requirements for clinical trials

International Committee of Medical Journal Editors (ICMJE)

 • To be considered for publication, clinical trials must be registered in public registry before enrolling participants [29, 30].

 • Reports of clinical trials must include a data sharing statement [57].

Food and Drug Administration Amendments Act of 2007 (FDAAA) and The Final Rule [31, 34]

 • Applicable clinical trials must be registered on ClinicalTrials.gov within 21 days of enrolling the first participant

 • Trial registrations must include the primary and secondary outcomes, including the specific measures and time-points that will be used to assess trial outcomes

 • Results must be reported within 12 months of the final data collection in support of the primary outcome. (The Final Rule expanded this requirement to include both approved and unapproved products.)

 • Results must include the primary and secondary outcomes, all serious adverse events, all-cause mortality, and adverse events occurring in 5% of participants

 • Results must include baseline information on age and gender and, if collected, for race or ethnic group

 • Records must be updatedas follows: within 15 days of changes to approval or clearance status; within 30 days of reaching the primary completion date; and within 30 days of changes to trial recruitment status, human subjects protection review board status, or responsible party

National Institutes of Health (NIH) [36]

 • Requirements for NIH-funded clinical trials mirror the requirements for applicable trials under FDAAA

 • All clinical trials funded by NIH (in whole or in part) must be registered, and their results must be reported, on ClinicalTrials.gov

## Box 2: Survey topics

Policies and procedures

 • Does the organization have a policy that requires investigators to register their trials? A results reporting policy?

 • Which trials are covered by these policies?

 • When did these policies come into effect?

 • Do these policies describe processes for investigators joining and leaving the organization?

 • Are there penalties for investigators who do not register their trials or report their results?

Staffing and support

 • Which functions do staff members perform (e.g., entering results, checking records, educating investigators)?

 • How many staff members are assigned to support trial registration and results reporting? How much time do they spend on these activities?

 • Are there plans to hire more staff in the future?

Monitoring systems

 • Does the organization have a system for monitoring trial registration and results reporting? For notifying investigators when results are due?

 • Does an IRB check whether trials are registered and reported?

## Additional files

Additional file 1: Survey instrument. (DOCX 500 kb)

Additional file 2: Eligible accounts. (DOCX 452 kb)

Additional file 3: Participating accounts. (DOCX 476 kb)

Additional file 4: Additional survey results. (DOCX 440 kb)

Additional file 5: Sensitivity analysis. (DOCX 436 kb)

## Abbreviations

API: Application programming interface
CTSA: Clinical and Translational Science Award
FDA: US Food and Drug Administration
FDAAA: Food and Drug Administration Amendments Act of 2007
HHS: Health and Human Services
ICMJE: International Committee of Medical Journal Editors
IRB: Institutional review board
NCI: National Cancer Institute
NIH: National Institutes of Health
NLM: National Library of Medicine
PRS: Protocol Registration and Results System

## References

[1] Ross JS, Mulvey GK, Hines EM, Nissen SE, Krumholz HM (2009). Trial publication after registration in ClinicalTrials.Gov: a cross-sectional analysis. PLoS Med.

[2] Dwan K, Gamble C, Williamson PR, Kirkham JJ, Reporting Bias Group (2013). Systematic review of the empirical evidence of study publication bias and outcome reporting bias – an updated review. PLoS One.

[3] Song F, Parekh S, Hooper L, Loke YK, Ryder J, Sutton AJ (2010). Dissemination and publication of research findings: an updated review of related biases. Health Technol Assess.

[4] Cruz ML, Xu J, Kashoki M, Lurie P (2017). Publication and Reporting of the Results of Postmarket Studies for Drugs Required by the US Food and Drug Administration, 2009 to 2013. JAMA Intern Med.

[5] Jones CW, Handler L, Crowell KE, Keil LG, Weaver MA, Platts-Mills TF (2013). Non-publication of large randomized clinical trials: cross sectional analysis. BMJ.

[6] Pica N, Bourgeois F. Discontinuation and nonpublication of randomized clinical trials conducted in children. Pediatrics. 2016;138(3) 10.1542/peds.2016-0223 PMC5005019 27492817

[7] Turner L, Shamseer L, Altman DG, Weeks L, Peters J, Kober T (2012). Consolidated Standards of Reporting Trials (CONSORT) and the completeness of reporting of randomised controlled trials (RCTs) published in medical journals. Cochrane Database Syst Rev.

[8] Wieseler B, Kerekes MF, Vervoelgyi V, McGauran N, Kaiser T (2012). Impact of document type on reporting quality of clinical drug trials: a comparison of registry reports, clinical study reports, and journal publications. BMJ.

[9] Dechartres A, Trinquart L, Atal I, Moher D, Dickersin K, Boutron I (2017). Evolution of poor reporting and inadequate methods over time in 20 920 randomised controlled trials included in Cochrane reviews: research on research study. BMJ.

[10] Glasziou P, Meats E, Heneghan C, Shepperd S (2008). What is missing from descriptions of treatment in trials and reviews?. BMJ.

[11] Dwan K, Altman DG, Cresswell L, Blundell M, Gamble CL, Williamson PR (2011). Comparison of protocols and registry entries to published reports for randomised controlled trials. Cochrane Database Syst Rev.

[12] Hart B, Lundh A, Bero L (2012). Effect of reporting bias on meta-analyses of drug trials: reanalysis of meta-analyses. BMJ.

[13] Rising K, Bacchetti P, Bero L (2008). Reporting bias in drug trials submitted to the Food and Drug Administration: review of publication and presentation. PLoS Med.

[14] Baudard M, Yavchitz A, Ravaud P, Perrodeau E, Boutron I (2017). Impact of searching clinical trial registries in systematic reviews of pharmaceutical treatments: methodological systematic review and reanalysis of meta-analyses. BMJ.

[15] Jones CW, Keil LG, Holland WC, Caughey MC, Platts-Mills TF (2015). Comparison of registered and published outcomes in randomized controlled trials: a systematic review. BMC Med.

[16] Pranic S, Marusic A (2016). Changes to registration elements and results in a cohort of ClinicalTrials.gov trials were not reflected in published articles. J Clin Epidemiol.

[17] Tang E, Ravaud P, Riveros C, Perrodeau E, Dechartres A (2015). Comparison of serious adverse events posted at ClinicalTrials.gov and published in corresponding journal articles. BMC Med.

[18] Chalmers I (1990). Underreporting research is scientific misconduct. JAMA.

[19] Chan AW, Song F, Vickers A, Jefferson T, Dickersin K, Gotzsche PC (2014). Increasing value and reducing waste: addressing inaccessible research. Lancet.

[20] Glasziou P, Altman DG, Bossuyt P, Boutron I, Clarke M, Julious S (2014). Reducing waste from incomplete or unusable reports of biomedical research. Lancet.

[21] Maund E, Tendal B, Hrobjartsson A, Jorgensen KJ, Lundh A, Schroll J (2014). Benefits and harms in clinical trials of duloxetine for treatment of major depressive disorder: comparison of clinical study reports, trial registries, and publications. BMJ.

[22] Simes R (1986). Publication bias: the case for an international registry of clinical trials. J Clin Oncol.

[23] Meinert CL (1988). Toward prospective registration of clinical trials. Control Clin Trials.

[24] Chalmers TC (1977). Randomize the first patient!. N Engl J Med.

[25] Levine J, Guy W, Cleary PA (1974). Therapeutic trials of psychopharmacologic agents: 1968–1972. In: McMahon FG, editor. Principles and techniques of human research and therapeutics. VIII.

[26] Dickersin K, Rennie D (2012). The evolution of trial registries and their use to assess the clinical trial enterprise. JAMA.

[27] Dickersin K, Rennie D (2003). Registering clinical trials. JAMA.

[28] Zarin DA, Tse T, Williams RJ, Califf RM, Ide NC (2011). The ClinicalTrials.gov results database—update and key issues. N Engl J Med.

[29] De Angelis CD, Drazen JM, Frizelle FA, Haug C, Hoey J, Horton R (2005). Is this clinical trial fully registered? A statement from the International Committee of Medical Journal Editors. JAMA.

[30] DeAngelis CD, Drazen JM, Frizelle FA, Haug C, Hoey J, Horton R (2004). Clinical trial registration: a statement from the International Committee of Medical Journal Editors. JAMA.

[31] 121 Stat. 823. Food and Drug Administration Amendments Act (FDAAA) of 2007. Public Law 110–85.

[32] 45 CFR 102.3. Adjustment of civil monetary penalties for inflation. Federal Register.

[33] Zarin DA, Tse T, Williams RJ, Carr S (2016). Trial reporting in ClinicalTrials.gov — The Final Rule. N Engl J Med.

[34] 42 CFR 11. Clinical trials registration and results information submission; Final rule. Federal Register. 27658315

[35] Hudson KL, Lauer MS, Collins FS (2016). Toward a new era of trust and transparency in clinical trials. JAMA.

[36] NOT-OD-16-149. National Institutes of Health. NIH policy on dissemination of NIH-funded clinical trial information. Federal Register.

[37] Zarin DA, Tse T, Williams RJ, Rajakannan T (2017). Update on trial registration 11 years after the ICMJE policy was established. N Engl J Med.

[38] Law MR, Kawasumi Y, Morgan SG (2011). Despite law, fewer than one in eight completed studies of drugs and biologics are reported on time on ClinicalTrials.gov. Health Aff (Millwood).

[39] Prayle AP, Hurley MN, Smyth AR (2012). Compliance with mandatory reporting of clinical trial results on ClinicalTrials.gov: cross sectional study. BMJ.

[40] Ross JS, Tse T, Zarin DA, Xu H, Zhou L, Krumholz HM (2012). Publication of NIH funded trials registered in ClinicalTrials.gov: cross sectional analysis. BMJ.

[41] Piller C. Law ignored, patients at risk. STAT December 15, 2015. https://www.statnews.com/2015/12/13/clinical-trials-investigation/. Accessed 24 Oct 2017.

[42] Chen R, Desai NR, Ross JS, Zhang W, Chau KH, Wayda B (2016). Publication and reporting of clinical trial results: cross sectional analysis across academic medical centers. BMJ.

[43] Anderson ML, Chiswell K, Peterson ED, Tasneem A, Topping J, Califf RM (2015). Compliance with results reporting at ClinicalTrials.gov. N Engl J Med.

[44] Zarin DA, Tse T, Ross JS (2015). Trial-results reporting and academic medical centers. N Engl J Med.

[45] TranspariMED (2017). Medical Research Ethics at Top UK Universities: Performance, Policies and Future Plans.

[46] Piller C, Bronshtein T. Faced with public pressure, research institutions step up reporting of clinical trial results. STAT January 9, 2018. https://www.statnews.com/2018/01/09/clinical-trials-reporting-nih/. Accessed 13 Feb 2018.

[47] Weber WE, Merino JG, Loder E (2015). Trial registration 10 years on. BMJ.

[48] Dal-Re R, Ross JS, Marusic A (2016). Compliance with prospective trial registration guidance remained low in high-impact journals and has implications for primary end point reporting. J Clin Epidemiol.

[49] Scott A, Rucklidge JJ, Mulder RT (2015). Is mandatory prospective trial registration working to prevent publication of unregistered trials and selective outcome reporting? An observational study of five psychiatry journals that mandate prospective clinical trial registration. PLoS One.

[50] Cybulski L, Mayo-Wilson E, Grant S. Improving transparency and reproducibility through registration: the status of intervention trials published in clinical psychology journals. J Consult Clin Psychol. 2016; 10.1037/ccp0000115. 10.1037/ccp0000115 27281372

[51] Viergever RF, Karam G, Reis A, Ghersi D (2014). The quality of registration of clinical trials: still a problem. PLoS One.

[52] Huic M, Marusic M, Marusic A (2011). Completeness and changes in registered data and reporting bias of randomized controlled trials in ICMJE journals after trial registration policy. PLoS One.

[53] Maruani A, Boutron I, Baron G, Ravaud P (2014). Impact of sending email reminders of the legal requirement for posting results on ClinicalTrials.gov: cohort embedded pragmatic randomized controlled trial. BMJ.

[54] Huang GD, Altemose JK, O'Leary TJ (2017). Public access to clinical trials: lessons from an organizational implementation of policy. Contemp Clin Trials.

[55] O'Reilly EK, Hassell NJ, Snyder DC, Natoli S, Liu I, Rimmler J (2015). ClinicalTrials.gov reporting: strategies for success at an academic health center. Clin Transl Sci.

[56] Evoniuk G, Mansi B, DeCastro B, Sykes J (2017). Impact of study outcome on submission and acceptance metrics for peer reviewed medical journals: six year retrospective review of all completed GlaxoSmithKline human drug research studies. BMJ.

[57] Taichman DB, Sahni P, Pinborg A, Peiperl L, Laine C, James A (2017). Data sharing statements for clinical trials: a requirement of the International Committee of Medical Journal Editors. JAMA.

[58] ICH (1996). Guideline for good clinical practice. International Conference on Harmonisation of Technical Requirements for Registration of Pharmaceuticals for Human Use (ICH).

[59] 46.102(g) C. Protection of Human Subjects. Code of Federal Regulations. 11660819

[60] 56.102 C. Institutional Review Boards. Code of Federal Regulations.

