# Supplementary material for: Clinical trial registration and reporting: a survey of academic organizations in the United States
Source: BMC Med. 2018 May 2;16:60. doi: 10.1186/s12916-018-1042-6 (PMC5930804; doi:10.1186/s12916-018-1042-6)
Supplement: Supplementary file 1 — Survey instrument. (DOCX 500 kb) [file 12916_2018_1042_MOESM1_ESM.docx]

**ONLINE SUPPLEMENTS**

**Additional file 1: Survey instrument**

*We exported the survey from Qualtrics as a Word document (below) on December 1, 2016.*

**Survey for PRS Administrators**

This survey contains questions about ClinicalTrials.gov registration and reporting policies and practices. All questions relate to a single PRS account. If you are the administrator for more than one PRS account, you may have received multiple invitation letters from us.  Please complete a separate survey for each account. We estimate it will take 20 minutes to complete the survey. If you cannot complete all of the questions now, you may return to them later. Depending on your answers, you may not see each question in the survey. By answering the questions in this survey, you provide your consent to use your responses for this study.

Q3 1a. What type of institutions use the PRS account "${m://ExternalDataReference}"? (Check all that apply.)

- Teaching hospital(s)
- NCI designated cancer center
- Other (not NCI designated) cancer center
- School of medicine
- School of public health
- School of social work
- School of arts and sciences
- School of nursing
- School of dentistry
- Other (please specify)
- Don't know

Display This Question:

If 1a. What type of institutions use the PRS account "${m://ExternalDataReference}"? (Check all that... Other (please specify) Is Selected

Q4 1b. Please describe what other type(s) of institutions use the PRS account "${m://ExternalDataReference}".

Display This Question:

If 1a. What type of institutions use the PRS account "${m://ExternalDataReference}"? (Check all that... NCI designated cancer center Is Selected

And 1a. What type of institutions use the PRS account "${m://ExternalDataReference}"? (Check all that... Teaching hospital(s) Is Selected

And 1a. What type of institutions use the PRS account "${m://ExternalDataReference}"? (Check all that... School of medicine Is Selected

Q5 Please select the number of each type of institution that uses the PRS account "${m://ExternalDataReference}".

|  | Select a number from the dropdown menu. | | | | | | | | | | | | | | | | | | | |
| --- | --- | --- | --- | --- | --- | --- | --- | --- | --- | --- | --- | --- | --- | --- | --- | --- | --- | --- | --- | --- |
|  | 1 | 2 | 3 | 4 | 5 | 6 | 7 | 8 | 9 | 10 | 11 | 12 | 13 | 14 | 15 | 16 | 17 | 18 | 19 | 20 |
| 1c. NCI designated cancer center |  |  |  |  |  |  |  |  |  |  |  |  |  |  |  |  |  |  |  |  |
| 1d. Teaching hospital(s) |  |  |  |  |  |  |  |  |  |  |  |  |  |  |  |  |  |  |  |  |
| 1e. School of medicine |  |  |  |  |  |  |  |  |  |  |  |  |  |  |  |  |  |  |  |  |

Display This Question:

If 1a. What type of institutions use the PRS account "${m://ExternalDataReference}"? (Check all that... NCI designated cancer center Is Selected

And 1a. What type of institutions use the PRS account "${m://ExternalDataReference}"? (Check all that... Teaching hospital(s) Is Selected

And 1a. What type of institutions use the PRS account "${m://ExternalDataReference}"? (Check all that... School of medicine Is Not Selected

Q6 Please select the number of each type of institution that uses the PRS account "${m://ExternalDataReference}".

|  | Select a number from the dropdown menu. | | | | | | | | | | | | | | | | | | | |
| --- | --- | --- | --- | --- | --- | --- | --- | --- | --- | --- | --- | --- | --- | --- | --- | --- | --- | --- | --- | --- |
|  | 1 | 2 | 3 | 4 | 5 | 6 | 7 | 8 | 9 | 10 | 11 | 12 | 13 | 14 | 15 | 16 | 17 | 18 | 19 | 20 |
| 1c. NCI designated cancer center |  |  |  |  |  |  |  |  |  |  |  |  |  |  |  |  |  |  |  |  |
| 1d. Teaching hospital(s) |  |  |  |  |  |  |  |  |  |  |  |  |  |  |  |  |  |  |  |  |

Display This Question:

If 1a. What type of institutions use the PRS account "${m://ExternalDataReference}"? (Check all that... NCI designated cancer center Is Selected

And 1a. What type of institutions use the PRS account "${m://ExternalDataReference}"? (Check all that... School of medicine Is Selected

And 1a. What type of institutions use the PRS account "${m://ExternalDataReference}"? (Check all that... Teaching hospital(s) Is Not Selected

Q7 Please select the number of each type of institution that uses the PRS account "${m://ExternalDataReference}".

|  | Select a number from the dropdown menu. | | | |
| --- | --- | --- | --- | --- |
|  | 1 | 2 | 3 | 4 |
| 1c. NCI designated cancer center |  |  |  |  |
| 1e. School of medicine |  |  |  |  |

Display This Question:

If 1a. What type of institutions use the PRS account "${m://ExternalDataReference}"? (Check all that... Teaching hospital(s) Is Selected

And 1a. What type of institutions use the PRS account "${m://ExternalDataReference}"? (Check all that... School of medicine Is Selected

And 1a. What type of institutions use the PRS account "${m://ExternalDataReference}"? (Check all that... NCI-designated cancer center Is Not Selected

Q8 Please select the number of each type of institution that uses the PRS account "${m://ExternalDataReference}".

|  | Select a number from the dropdown menu. | | | | | | | | | | | | | | | | | | | |
| --- | --- | --- | --- | --- | --- | --- | --- | --- | --- | --- | --- | --- | --- | --- | --- | --- | --- | --- | --- | --- |
|  | 1 | 2 | 3 | 4 | 5 | 6 | 7 | 8 | 9 | 10 | 11 | 12 | 13 | 14 | 15 | 16 | 17 | 18 | 19 | 20 |
| 1d.Teaching hospital(s) |  |  |  |  |  |  |  |  |  |  |  |  |  |  |  |  |  |  |  |  |
| 1e. School of medicine |  |  |  |  |  |  |  |  |  |  |  |  |  |  |  |  |  |  |  |  |

Display This Question:

If 1a. What type of institutions use the PRS account "${m://ExternalDataReference}"? (Check all that apply.) NCI-designated cancer center Is Selected

And 1a. What type of institutions use the PRS account "${m://ExternalDataReference}"? (Check all that apply.) Teaching hospital(s) Is Not Selected

And 1a. What type of institutions use the PRS account "${m://ExternalDataReference}"? (Check all that apply.) School of medicine Is Not Selected

Q9 Please select the number of NCI designated cancer centers that use the PRS account "${m://ExternalDataReference}".

|  | Select a number from the dropdown menu. | | | |
| --- | --- | --- | --- | --- |
|  | 1 | 2 | 3 | 4 |
| 1c. NCI designated cancer center |  |  |  |  |

Display This Question:

If 1a. What type of institutions use the PRS account "${m://ExternalDataReference}"? (Check all that apply.) Teaching hospital(s) Is Selected

And 1a. What type of institutions use the PRS account "${m://ExternalDataReference}"? (Check all that apply.) School of medicine Is Not Selected

And 1a. What type of institutions use the PRS account "${m://ExternalDataReference}"? (Check all that apply.) NCI-designated cancer center Is Not Selected

Q10 Please select the number of teaching hospitals that use the PRS account "${m://ExternalDataReference}".

|  | Select a number from the dropdown menu. | | | | | | | | | | | | | | | | | | | |
| --- | --- | --- | --- | --- | --- | --- | --- | --- | --- | --- | --- | --- | --- | --- | --- | --- | --- | --- | --- | --- |
|  | 1 | 2 | 3 | 4 | 5 | 6 | 7 | 8 | 9 | 10 | 11 | 12 | 13 | 14 | 15 | 16 | 17 | 18 | 19 | 20 |
| 1d. Teaching hospital(s) |  |  |  |  |  |  |  |  |  |  |  |  |  |  |  |  |  |  |  |  |

Display This Question:

If 1a. What type of institutions use the PRS account "${m://ExternalDataReference}"? (Check all that apply.) School of medicine Is Selected

And 1a. What type of institutions use the PRS account "${m://ExternalDataReference}"? (Check all that apply.) Teaching hospital(s) Is Not Selected

And 1a. What type of institutions use the PRS account "${m://ExternalDataReference}"? (Check all that apply.) NCI-designated cancer center Is Not Selected

Q11 Please select the number of schools of medicine that use the PRS account "${m://ExternalDataReference}".

|  | Select a number from the dropdown menu. | | | |
| --- | --- | --- | --- | --- |
|  | 1 | 2 | 3 | 4 |
| 1e. School of medicine |  |  |  |  |

Display This Question:

If 1c. Please select the number of each type of institution that registers its trials using this PRS... NCI-designated cancer center - Select a number from the dropdown menu. - 1 Is Selected

Or 1d. Please select the number of each type of institution that registers its trials using this PRS... NCI-designated cancer center - Select a number from the dropdown menu. - 1 Is Selected

Or 1e. Please select the number of each type of institution that registers its trials using this PRS... NCI-designated cancer center - Select a number from the dropdown menu. - 1 Is Selected

Or 1g. Please select the number of NCI-designated cancer centers that register their trials using th... NCI-designated cancer center - Select a number from the dropdown menu. - 1 Is Selected

Q12 1f. Please select the NCI designated cancer center that uses the PRS account "${m://ExternalDataReference}".

- Abramson Cancer Center
- Albert Einstein Cancer Center
- Alvin J. Siteman Cancer Center
- Arizona Cancer Center
- Barbara Ann Karmanos Cancer Institute
- Cancer Therapy & Research Center
- Case Comprehensive Cancer Center
- Chao Family Comprehensive Cancer Center
- City of Hope Comprehensive Cancer Center
- Cold Spring Harbor Laboratory Cancer Center
- Comprehensive Cancer Center James Cancer Hospital & Solove Research Institute
- Dan L Duncan Comprehensive Cancer Center
- Dana Farber / Harvard Cancer Center
- David H. Koch Institute for Integrative Cancer Research at MIT
- Duke Cancer Institute
- Fox Chase Cancer Center
- Fred & Pamela Buffett Cancer Center
- Fred Hutchinson / University of Washington Cancer Consortium
- Georgetown Lombardi Comprehensive Cancer Center
- Greenebaum Cancer Center
- Harold C. Simmons Comprehensive Cancer Center
- Herbert Irving Comprehensive Cancer Center
- Holden Comprehensive Cancer Center
- Hollings Cancer Center
- Huntsman Cancer Institute
- Indiana University Melvin & Bren Simon Cancer Center
- Jonsson Comprehensive Cancer Center
- Markey Cancer Center
- Masonic Cancer Center
- Massey Cancer Center
- Mayo Clinic Cancer Center
- MD Anderson Cancer Center
- Memorial Sloan Kettering Cancer Center
- Moffitt Cancer Center
- Moores Comprehensive Cancer Center
- Norris Cotton Cancer Center
- NYU Cancer Institute
- OHSU Knight Cancer Institute
- Purdue University Center for Cancer Research
- Robert H. Lurie Comprehensive Cancer Center
- Roswell Park Cancer Institute
- Rutgers Cancer Institute of New Jersey
- Salk Institute Cancer Center
- Sanford Burnham Prebys Medical Discovery Institute
- Sidney Kimmel Cancer Center at Thomas Jefferson University
- Sidney Kimmel Comprehensive Cancer Center
- St. Jude Children's Research Hospital
- Stanford Cancer Institute
- The Jackson Laboratory Cancer Center
- The Wistar Institute Cancer Center
- Tisch Cancer Institute
- UAB Comprehensive Cancer Center
- UC Davis Comprehensive Cancer Center
- UCSF Helen Diller Family Comprehensive Cancer Center
- UNC Lineberger Comprehensive Cancer Center
- University of Chicago Comprehensive Cancer Center
- University of Colorado Cancer Center
- University of Hawaii Cancer Center
- University of Kansas Cancer Center
- University of Michigan Comprehensive Cancer Center
- University of New Mexico Cancer Research & Treatment Center
- University of Pittsburgh Cancer Institute
- University of Wisconsin Carbone Cancer Center
- USC Norris Comprehensive Cancer Center
- UVA Cancer Center
- Vanderbilt Ingram Cancer Center
- Wake Forest Comprehensive Cancer Center
- Winship Cancer Institute of Emory University
- Yale Cancer Center

Display This Question:

If Please select the number of each type of institution that uses the PRS account "... 1c. NCI designated cancer center - Select a number from the dropdown menu. - 2 Is Selected

Or Please select the number of each type of institution that uses the PRS account "... 1c. NCI designated cancer center - Select a number from the dropdown menu. - 3 Is Selected

Or Please select the number of each type of institution that uses the PRS account "... 1c. NCI designated cancer center - Select a number from the dropdown menu. - 4 Is Selected

Or Please select the number of each type of institution that uses the PRS account "... 1c. NCI designated cancer center - Select a number from the dropdown menu. - 5 Is Selected

Or Please select the number of each type of institution that uses the PRS account "... 1c. NCI designated cancer center - Select a number from the dropdown menu. - 6 Is Selected

Or Please select the number of each type of institution that uses the PRS account "... 1c. NCI designated cancer center - Select a number from the dropdown menu. - 7 Is Selected

Or Please select the number of each type of institution that uses the PRS account "... 1c. NCI designated cancer center - Select a number from the dropdown menu. - 8 Is Selected

Or Please select the number of each type of institution that uses the PRS account "... 1c. NCI designated cancer center - Select a number from the dropdown menu. - 9 Is Selected

Or Please select the number of each type of institution that uses the PRS account "... 1c. NCI designated cancer center - Select a number from the dropdown menu. - 10 Is Selected

Or Please select the number of each type of institution that uses the PRS account "... 1c. NCI designated cancer center - Select a number from the dropdown menu. - 11 Is Selected

Or Please select the number of each type of institution that uses the PRS account "... 1c. NCI designated cancer center - Select a number from the dropdown menu. - 12 Is Selected

Or Please select the number of each type of institution that uses the PRS account "... 1c. NCI designated cancer center - Select a number from the dropdown menu. - 13 Is Selected

Or Please select the number of each type of institution that uses the PRS account "... 1c. NCI designated cancer center - Select a number from the dropdown menu. - 14 Is Selected

Or Please select the number of each type of institution that uses the PRS account "... 1c. NCI designated cancer center - Select a number from the dropdown menu. - 15 Is Selected

Or Please select the number of each type of institution that uses the PRS account "... 1c. NCI designated cancer center - Select a number from the dropdown menu. - 16 Is Selected

Or Please select the number of each type of institution that uses the PRS account "... 1c. NCI designated cancer center - Select a number from the dropdown menu. - 17 Is Selected

Or Please select the number of each type of institution that uses the PRS account "... 1c. NCI designated cancer center - Select a number from the dropdown menu. - 18 Is Selected

Or Please select the number of each type of institution that uses the PRS account "... 1c. NCI designated cancer center - Select a number from the dropdown menu. - 19 Is Selected

Or Please select the number of each type of institution that uses the PRS account "... 1c. NCI designated cancer center - Select a number from the dropdown menu. - 20 Is Selected

Or Please select the number of each type of institution that uses the PRS account "... 1c. NCI designated cancer center - Select a number from the dropdown menu. - 2 Is Selected

Or Please select the number of each type of institution that uses the PRS account "... 1c. NCI designated cancer center - Select a number from the dropdown menu. - 3 Is Selected

Or Please select the number of each type of institution that uses the PRS account "... 1c. NCI designated cancer center - Select a number from the dropdown menu. - 4 Is Selected

Or Please select the number of each type of institution that uses the PRS account "... 1c. NCI designated cancer center - Select a number from the dropdown menu. - 5 Is Selected

Or Please select the number of each type of institution that uses the PRS account "... 1c. NCI designated cancer center - Select a number from the dropdown menu. - 6 Is Selected

Or Please select the number of each type of institution that uses the PRS account "... 1c. NCI designated cancer center - Select a number from the dropdown menu. - 7 Is Selected

Or Please select the number of each type of institution that uses the PRS account "... 1c. NCI designated cancer center - Select a number from the dropdown menu. - 8 Is Selected

Or Please select the number of each type of institution that uses the PRS account "... 1c. NCI designated cancer center - Select a number from the dropdown menu. - 9 Is Selected

Or Please select the number of each type of institution that uses the PRS account "... 1c. NCI designated cancer center - Select a number from the dropdown menu. - 10 Is Selected

Or Please select the number of each type of institution that uses the PRS account "... 1c. NCI designated cancer center - Select a number from the dropdown menu. - 11 Is Selected

Or Please select the number of each type of institution that uses the PRS account "... 1c. NCI designated cancer center - Select a number from the dropdown menu. - 12 Is Selected

Or Please select the number of each type of institution that uses the PRS account "... 1c. NCI designated cancer center - Select a number from the dropdown menu. - 13 Is Selected

Or Please select the number of each type of institution that uses the PRS account "... 1c. NCI designated cancer center - Select a number from the dropdown menu. - 14 Is Selected

Or Please select the number of each type of institution that uses the PRS account "... 1c. NCI designated cancer center - Select a number from the dropdown menu. - 15 Is Selected

Or Please select the number of each type of institution that uses the PRS account "... 1c. NCI designated cancer center - Select a number from the dropdown menu. - 16 Is Selected

Or Please select the number of each type of institution that uses the PRS account "... 1c. NCI designated cancer center - Select a number from the dropdown menu. - 17 Is Selected

Or Please select the number of each type of institution that uses the PRS account "... 1c. NCI designated cancer center - Select a number from the dropdown menu. - 18 Is Selected

Or Please select the number of each type of institution that uses the PRS account "... 1c. NCI designated cancer center - Select a number from the dropdown menu. - 19 Is Selected

Or Please select the number of each type of institution that uses the PRS account "... 1c. NCI designated cancer center - Select a number from the dropdown menu. - 20 Is Selected

Or Please select the number of each type of institution that uses the PRS account "... 1c. NCI designated cancer center - Select a number from the dropdown menu. - 2 Is Selected

Or Please select the number of each type of institution that uses the PRS account "... 1c. NCI designated cancer center - Select a number from the dropdown menu. - 3 Is Selected

Or Please select the number of each type of institution that uses the PRS account "... 1c. NCI designated cancer center - Select a number from the dropdown menu. - 4 Is Selected

Or Please select the number of NCI designated cancer centers that use the PRS account "... 1c. NCI designated cancer center - Select a number from the dropdown menu. - 2 Is Selected

Or Please select the number of NCI designated cancer centers that use the PRS account "... 1c. NCI designated cancer center - Select a number from the dropdown menu. - 3 Is Selected

Or Please select the number of NCI designated cancer centers that use the PRS account "... 1c. NCI designated cancer center - Select a number from the dropdown menu. - 4 Is Selected

Q13 1g. Please select the NCI designated cancer centers that use the PRS account "${m://ExternalDataReference}". (Check all that apply.)

- Abramson Cancer Center
- Albert Einstein Cancer Center
- Alvin J. Siteman Cancer Center
- Arizona Cancer Center
- Barbara Ann Karmanos Cancer Institute
- Cancer Therapy & Research Center
- Case Comprehensive Cancer Center
- Chao Family Comprehensive Cancer Center
- City of Hope Comprehensive Cancer Center
- Cold Spring Harbor Laboratory Cancer Center
- Comprehensive Cancer Center James Cancer Hospital & Solove Research Institute
- Dan L Duncan Comprehensive Cancer Center
- Dana Farber / Harvard Cancer Center
- David H. Koch Institute for Integrative Cancer Research at MIT
- Duke Cancer Institute
- Fox Chase Cancer Center
- Fred & Pamela Buffett Cancer Center
- Fred Hutchinson / University of Washington Cancer Consortium
- Georgetown Lombardi Comprehensive Cancer Center
- Greenebaum Cancer Center
- Harold C. Simmons Comprehensive Cancer Center
- Herbert Irving Comprehensive Cancer Center
- Holden Comprehensive Cancer Center
- Hollings Cancer Center
- Huntsman Cancer Institute
- Indiana University Melvin & Bren Simon Cancer Center
- Jonsson Comprehensive Cancer Center
- Markey Cancer Center
- Masonic Cancer Center
- Massey Cancer Center
- Mayo Clinic Cancer Center
- MD Anderson Cancer Center
- Memorial Sloan Kettering Cancer Center
- Moffitt Cancer Center
- Moores Comprehensive Cancer Center
- Norris Cotton Cancer Center
- NYU Cancer Institute
- OHSU Knight Cancer Institute
- Purdue University Center for Cancer Research
- Robert H. Lurie Comprehensive Cancer Center
- Roswell Park Cancer Institute
- Rutgers Cancer Institute of New Jersey
- Salk Institute Cancer Center
- Sanford Burnham Prebys Medical Discovery Institute
- Sidney Kimmel Cancer Center at Thomas Jefferson University
- Sidney Kimmel Comprehensive Cancer Center
- St. Jude Children's Research Hospital
- Stanford Cancer Institute
- The Jackson Laboratory Cancer Center
- The Wistar Institute Cancer Center
- Tisch Cancer Institute
- UAB Comprehensive Cancer Center
- UC Davis Comprehensive Cancer Center
- UCSF Helen Diller Family Comprehensive Cancer Center
- UNC Lineberger Comprehensive Cancer Center
- University of Chicago Comprehensive Cancer Center
- University of Colorado Cancer Center
- University of Hawaii Cancer Center
- University of Kansas Cancer Center
- University of Michigan Comprehensive Cancer Center
- University of New Mexico Cancer Research & Treatment Center
- University of Pittsburgh Cancer Institute
- University of Wisconsin Carbone Cancer Center
- USC Norris Comprehensive Cancer Center
- UVA Cancer Center
- Vanderbilt Ingram Cancer Center
- Wake Forest Comprehensive Cancer Center
- Winship Cancer Institute of Emory University
- Yale Cancer Center

Display This Question:

If Please select the number of each type of institution that uses the PRS account "... 1d. Teaching hospital(s) - Select a number from the dropdown menu. - 1 Is Selected

Or Please select the number of each type of institution that uses the PRS account "... 1d. Teaching hospital(s) - Select a number from the dropdown menu. - 1 Is Selected

Or Please select the number of each type of institution that uses the PRS account "... 1d.Teaching hospital(s) - Select a number from the dropdown menu. - 1 Is Selected

Or Please select the number of teaching hospitals that use the PRS account "... 1d. Teaching hospital(s) - Select a number from the dropdown menu. - 1 Is Selected

Q14 1h. Please select the teaching hospital or network (e.g. Mayo Health Network) that uses the PRS account "${m://ExternalDataReference}".

- Advocate Christ Medical Center
- Advocate Health Care
- Advocate Illinois Masonic Medical Center
- Advocate Lutheran General Hospital
- Akron Children's Hospital
- Akron General Medical Center
- Albany Medical Center Hospital
- Albany Veterans Affairs Medical Center
- Albert Einstein Medical Center (Albert Einstein Healthcare Network)
- All Children's Hospital
- Allegheny General Hospital
- Anne Arundel Medical Center
- Arkansas Children's Hospital
- Atlantic Health
- Augusta University Medical Center
- Augusta Veterans Affairs Medical Center
- Aurora Health Care
- Banner University Medical Center Phoenix
- Banner University Medical Center Tucson Campus
- Bannerhealth
- Barnabas Health
- Barnes Jewish Hospital
- Bassett Healthcare
- Baton Rouge General Medical Center
- Baylor St. Luke’s Medical Center
- Baylor University Medical Center
- Baystate Health System
- Baystate Medical Center
- Beaumont Health
- Beaumont Dearborn
- Beaumont Royal Oak
- Berkshire Medical Center
- Beth Israel Deaconess Medical Center
- Birmingham Veterans Affairs Medical Center
- BJC HealthCare
- Boston Children's Hospital
- Boston Medical Center
- Bridgeport Hospital
- Brigham and Women's Hospital
- Cabell Huntington Hospital
- California Pacific Medical Center
- Cambridge Health Alliance
- Cambridge Health Alliance Cambridge Hospital Campus
- CAMC Health System
- Captain James A. Lovell Federal Health Care Center
- Care New England Health System
- CareGroup Inc.
- Carilion Medical Center
- Carolinas HealthCare System
- Carolinas Medical Center
- Cedars Sinai Medical Center
- Central Arkansas Veterans Healthcare System
- Central Texas Veterans Health Care Sys
- Charleston Area Medical Center
- CHI Health
- CHI Health Creighton University Medical Center Bergan Mercy
- CHI St. Luke’s Health
- Children's Healthcare of Atlanta
- Children's Healthcare of Atlanta (Includes Egleston and Scottish Rite)
- Children's Hospital Los Angeles
- Children's Hospital of Philadelphia
- Children's Medical Center of Dallas
- Children's Memorial Hospital
- Children's Mercy Hospital
- Children's National Medical Center
- Christiana Care Health System
- Cincinnati Children's Hospital Medical Center
- City of Hope National Medical Center
- Cleveland Clinic Foundation
- Cleveland Clinic Health System
- Community Health Network
- Community Regional Medical Center
- Cooper University Hospital
- Dana Farber Cancer Institute
- Danbury Hospital
- Dartmouth Hitchcock Alliance
- Dartmouth Hitchcock Medical Center
- Dayton Veterans Affairs Medical Center
- Denver Health and Hospital Authority
- Department of Veterans Affairs Veterans Integrated Service Network VISN 1
- Department of Veterans Affairs Veterans Integrated Service Network VISN 10
- Department of Veterans Affairs Veterans Integrated Service Network VISN 11
- Department of Veterans Affairs Veterans Integrated Service Network VISN 12
- Department of Veterans Affairs Veterans Integrated Service Network VISN 17
- Department of Veterans Affairs Veterans Integrated Service Network VISN 18
- Department of Veterans Affairs Veterans Integrated Service Network VISN 2
- Department of Veterans Affairs Veterans Integrated Service Network VISN 22
- Department of Veterans Affairs Veterans Integrated Service Network VISN 23
- Department of Veterans Affairs Veterans Integrated Service Network VISN 4
- Department of Veterans Affairs Veterans Integrated Service Network VISN 5
- Department of Veterans Affairs Veterans Integrated Service Network VISN 6
- Department of Veterans Affairs Veterans Integrated Service Network VISN 7
- Department of Veterans Affairs Veterans Integrated Service Network VISN 8
- Department of Veterans Affairs Veterans Integrated Service Network VISN 9
- Dignity Health
- Duke University Health System
- Duke University Hospital
- Durham Veterans Affairs Medical Center
- Edward W. Sparrow Hospital
- Emory Healthcare
- Emory University Hospital
- Emory University Hospital Midtown
- Erlanger Health System
- Erlanger Medical Center
- Eskenazi Health
- Fairview Health Services
- Florida Hospital Orlando
- Franklin Square Hospital
- Froedtert Hospital
- Geisinger Medical Center
- General Health System
- George Washington University Hospital
- Georgetown University Hospital
- Good Samaritan Hospital (TriHealth)
- Grady Memorial Hospital Atlanta GA
- Grant-Riverside Methodist Hospitals Grant Medical Center Campus
- Grant-Riverside Methodist Hospitals Riverside Campus
- Greenville Health System
- Greenwich Hospital
- Gundersen Health System
- Gundersen Lutheran Medical Center
- Gwinnett Medical Center Lawrenceville
- Hackensack University Medical Center
- Harbor UCLA Medical Center
- Harborview Medical Center
- HealthPartners Inc.
- Hennepin County Medical Center
- Henry Ford Health System
- Henry Ford Hospital
- HonorHealth Scottsdale Osborn Medical Center
- Hospital for Special Surgery
- Hospital of the University of Pennsylvania
- Houston Methodist
- Houston Methodist Hospital
- Howard University Hospital
- Hunter Holmes McGuire Veterans Affairs Medical Center
- Hurley Medical Center
- Indiana University Health Methodist Hospital
- Indiana University Health Inc
- INOVA Fairfax Hospital
- INOVA Health System
- Iowa City Veterans Affairs Medical Center
- Jackson Memorial Hospital
- James J. Peters VA Medical Center
- Jefferson Health System
- Jerry L. Pettis Memorial Veterans Affair Medical Center
- Jersey Shore University Medical Center
- Jesse Brown VA Medical Center
- John H. Stroger Jr. Hospital of Cook County
- John Peter Smith Hospital (Tarrant County Hospital District)
- Johns Hopkins Bayview Medical Center
- Johns Hopkins Health System
- Johns Hopkins Hospital
- Johnson City Medical Center
- Kaiser Foundation Hospital Los Angeles
- Kaiser Foundation Hospitals Northern California
- Kaiser Permanente Foundation Hospitals Southern California
- Keck Medical Center of USC
- KentuckyOne Health University of Louisville Hospital
- Kettering Medical Center
- Lahey Hospital and Medical Center
- Lankenau Hospital
- LeBonheur Children's Hospital
- Lehigh Valley Health Network
- Lehigh Valley Hospital Cedar Crest
- Lenox Hill Healthcare Network
- Lenox Hill Hospital
- Lifespan Inc.
- Loma Linda University Medical Center
- Long Beach Memorial Medical Center
- Long Island Jewish Medical Center
- Louis Stokes Veterans Affairs Medical Center
- Loyola University Health System
- Loyola University Medical Center
- MacNeal Hospital
- Maimonides Medical Center
- Main Line Health
- Maine Medical Center
- Maricopa Medical Center
- Mary Imogene Bassett Hospital
- Massachusetts General Hospital
- Mayo Clinic Hospital Phoenix AZ
- Mayo Clinic Hospital Jacksonville FL
- Mayo Clinic Hospital Rochester MN
- Mayo Health System
- MCG Health Inc dba Augusta University Health Inc.
- McLaren Healthcare Corporation
- McLaren Regional Medical Center
- Medical Center Hospital
- Medical Center Navicent Health
- Medical University of South Carolina Medical Center
- MedStar Health
- Memorial Health System
- Memorial Health University Medical Center
- Memorial Health Inc.
- Memorial Hermann Texas Medical Center
- Memorial Hospital of Rhode Island
- Memorial Medical Center
- Memorial Sloan Kettering Cancer Center
- Mercy Hospital St. Louis
- Meridian Health System
- Methodist Healthcare University Hospital
- MetroHealth Medical Center
- MetroHealth System
- Miami Children's Hospital
- Michael E. DeBakey Veterans Affairs Medical Center
- Minneapolis Veterans Affairs Medical Center
- Miriam Hospital
- Monmouth Medical Center
- Montefiore Medical Center
- Morristown Memorial Hospital
- Mount Auburn Hospital
- Mount Sinai Beth Israel
- Mount Sinai Health System
- Mount Sinai Hospital
- Mount Sinai Medical Center
- Mountain States Health Alliance
- Mt. Sinai St. Luke's/Roosevelt
- National Jewish Health
- Navicent Health
- New Mexico Veterans Affairs Medical Center
- New York Eye and Ear Infirmary of Mount Sinai
- New York Methodist Hospital
- New York Presbyterian Hospital
- New York Presbyterian Healthcare System
- Newark Beth Israel Medical Center
- North Shore University Hospital
- North Shore Long Island Jewish Health System
- NorthShore University Health System Evanston Hospital
- Northwestern Memorial Hospital
- Norwalk Hospital
- NYU Hospitals Center
- NYU Langone Medical Center
- Ochsner Clinic Foundation
- Ohio State University Health System
- OhioHealth
- Oklahoma City Veterans Affairs Medical Center
- Oregon Health & Science University
- Orlando VA Medical Center
- OSF HealthCare System
- OSF Saint Francis Medical Center
- OU Medical Center
- Our Lady of The Lake Regional Medical Center
- Overlook Medical Center
- Palmetto Health
- Palmetto Health Alliance
- Parkland Health & Hospital System
- Partners HealthCare System Inc.
- Penn State Hershey Medical Center
- Philadelphia Veterans Affairs Medical Center
- Phoenix VA Health Care System
- Portland Veterans Affairs Medical Center
- Ralph H. Johnson Veterans Affairs Medical Center
- Reading Hospital and Medical Center
- Regions Hospital
- Rehabilitation Institute of Chicago
- Rhode Island Hospital
- Richard L. Roudebush Veterans Affairs Medical Center
- Robert Wood Johnson Health System
- Robert Wood Johnson University Hospital
- Roger Williams Medical Center
- Ronald Reagan UCLA Medical Center
- Roswell Park Cancer Institute
- Rush System for Health
- Rush University Medical Center
- Saint Barnabas Medical Center
- Saint Francis Care
- Saint Francis Hospital and Medical Center
- Saint Louis University Hospital
- Saint Luke's Health System
- Saint Luke's Hospital of Kansas City
- Saint Peter's University Hospital
- Salem Veterans Affairs Medical Center
- San Francisco General Hospital and Medical Center
- Sanford Medical Center
- Sanford USD Medical Center
- Scott & White Hospital Temple
- Scripps Green Hospital
- Scripps Health
- Seattle Children's Hospital
- Sentara Norfolk General Hospital
- Sinai Health System
- Sinai Samaritan Medical Center
- Sioux Falls VA Health Care System
- Sioux Valley Hospitals and Health System
- Southeast Louisiana Veterans Health Care System
- Southern Arizona Veterans Affairs Health Care System
- Spectrum Health
- Spectrum Health Butterworth Hospital
- St. Christopher's Hospital for Children
- St. Elizabeth's Medical Center
- St. John Health System
- St. John Hospital and Medical Center
- St. Joseph Mercy Hospital
- St. Joseph's Hospital & Medical Center
- St. Louis Children's Hospital
- St. Luke's Hospital
- St. Luke's Medical Center
- St. Vincent Indianapolis Hospital
- Stanford Health Care
- Staten Island University Hospital
- Steward Health Care System
- Stony Brook University Hospital
- Strong Health System
- Strong Memorial Hospital
- Summa Akron City Hospital
- SUNY Downstate Medical Center/University Hospital of Brooklyn
- Syracuse Veterans Affairs Medical Center
- Tampa General Hospital
- Temple University Health System
- Temple University Hospital
- Tenet Health System Hahnemann University Hospital
- Tenet Healthcare Corporation
- Texas Children's Hospital
- The Mount Sinai Hospital
- The National Institutes of Health (NIH) Clinical Center
- The Nebraska Medical Center
- The University of Texas MD Anderson Cancer Center
- Thomas Jefferson University Hospital
- Trinity Health
- Truman Medical Center Hospital Hill
- Tufts Medical Center
- Tulane Medical Center
- UAB Health System University of Alabama at Birmingham
- UC Health
- UCLA Health
- UCSD Healthcare
- UCSF Medical Center
- UF Health Jacksonville
- UF Shands Hospital
- UMass Memorial Medical Center
- UNC Health Care System
- United Health Services Hospitals
- UnityPoint Health Des Moines
- Univ of Connecticut Health Center/John Dempsey Hospital
- Universal Health Services Inc. George Washington University Hospital
- University Health System
- University Hospital SUNY Upstate Medical University
- University Hospitals and Clinics/ University of Mississippi Medical Center
- University Hospitals Case Medical Center
- University Hospitals HealthSystem
- University Medical Center New Orleans
- University of Alabama Hospital
- University of Arkansas for Medical Sciences
- University of California Davis Health System
- University of California Irvine Medical Center
- University of California San Diego Medical Center
- University of Chicago Hospitals and Health System
- University of Chicago Medical Center
- University of Cincinnati Medical Center
- University of Colorado Health
- University of Colorado Hospital
- University of Illinois at Chicago Medical Center
- University of Iowa Hospitals and Clinics
- University of Kansas Hospital
- University of Kentucky Hospital
- University of Maryland Medical Center
- University of Maryland Medical System
- University of Miami Hospital
- University of Michigan Health System
- University of Michigan Medical Center
- University of Minnesota Medical Center Fairview
- University of Missouri Hospitals and Clinics
- University of New Mexico Hospital
- University of North Carolina Hospitals
- University of Pennsylvania Health System
- University of South Alabama Medical Center
- University of Tennessee Medical Center
- University of Texas Health Science Center at Tyler
- University of Toledo Medical Center
- University of Utah Health System
- University of Utah Hospital
- University of Vermont Medical Center
- University of Virginia Medical Center
- University of Washington Medical Center
- University of Wisconsin Hospital and Clinics
- UPMC
- UPMC Hamot
- UPMC Presbyterian Shadyside
- USC Norris Cancer Hospital
- UT Southwestern Medical Center
- UW Medicine
- VA Caribbean Healthcare System
- VA Connecticut Health Care System
- VA Nebraska Western Iowa Health Care System-Omaha Division
- VA Sierra Pacific Network (10N21) Department of Veterans Affairs VISN 21
- Valley Children's Healthcare
- Vanderbilt University Medical Center
- Vanguard Health System
- VCU Medical Center
- Veterans Affairs Ann Arbor Healthcare System
- Veterans Affairs Boston Healthcare System
- Veterans Affairs Greater Los Angeles Health Care System
- Veterans Affairs Long Beach Healthcare System
- Veterans Affairs Maryland Health Care System
- Veterans Affairs Medical and Regional Office Center
- Veterans Affairs Medical Center (Atlanta)
- Veterans Affairs Medical Center James A. Haley Veterans Hospital
- Veterans Affairs Medical Center Memphis Tennessee
- Veterans Affairs New York Harbor Health Care System New York Campus
- Veterans Affairs Pittsburgh Healthcare System
- Veterans Affairs Puget Sound Health Care System
- Veterans Affairs San Diego Healthcare System
- Veterans Affairs South Texas Health Care System
- Veterans Affairs Tennessee Valley Health Care System
- Veterans Affairs Western New York Healthcare System
- Vidant Health
- Vidant Medical Center
- Wake Forest Baptist Medical Center
- Washington Hospital Center
- Wellspan Health
- Wellspan York Hospital
- Wellstar Atlanta Medical Center
- West Kendall Baptist Hospital
- West Penn Allegheny Health System
- West Virginia United Health System
- West Virginia University Hospitals Inc.
- Westchester Medical Center
- Western Pennsylvania Hospital
- Wexner Medical Center at The Ohio State University
- White Memorial Medical Center
- White River Junction VA Medical Center
- William Jennings Bryan Dorn VA Medical Center
- Winthrop South Nassau University Health System Inc.
- Winthrop University Hospital
- Women and Infants Hospital of Rhode Island
- Yale New Haven Health System
- Yale New Haven Hospital
- Other (please specify)

Display This Question:

If Please select the number of each type of institution that uses the PRS account "... 1d. Teaching hospital(s) - Select a number from the dropdown menu. - 2 Is Selected

Or Please select the number of each type of institution that uses the PRS account "... 1d. Teaching hospital(s) - Select a number from the dropdown menu. - 3 Is Selected

Or Please select the number of each type of institution that uses the PRS account "... 1d. Teaching hospital(s) - Select a number from the dropdown menu. - 4 Is Selected

Or Please select the number of each type of institution that uses the PRS account "... 1d. Teaching hospital(s) - Select a number from the dropdown menu. - 5 Is Selected

Or Please select the number of each type of institution that uses the PRS account "... 1d. Teaching hospital(s) - Select a number from the dropdown menu. - 6 Is Selected

Or Please select the number of each type of institution that uses the PRS account "... 1d. Teaching hospital(s) - Select a number from the dropdown menu. - 7 Is Selected

Or Please select the number of each type of institution that uses the PRS account "... 1d. Teaching hospital(s) - Select a number from the dropdown menu. - 8 Is Selected

Or Please select the number of each type of institution that uses the PRS account "... 1d. Teaching hospital(s) - Select a number from the dropdown menu. - 9 Is Selected

Or Please select the number of each type of institution that uses the PRS account "... 1d. Teaching hospital(s) - Select a number from the dropdown menu. - 10 Is Selected

Or Please select the number of each type of institution that uses the PRS account "... 1d. Teaching hospital(s) - Select a number from the dropdown menu. - 11 Is Selected

Or Please select the number of each type of institution that uses the PRS account "... 1d. Teaching hospital(s) - Select a number from the dropdown menu. - 12 Is Selected

Or Please select the number of each type of institution that uses the PRS account "... 1d. Teaching hospital(s) - Select a number from the dropdown menu. - 13 Is Selected

Or Please select the number of each type of institution that uses the PRS account "... 1d. Teaching hospital(s) - Select a number from the dropdown menu. - 14 Is Selected

Or Please select the number of each type of institution that uses the PRS account "... 1d. Teaching hospital(s) - Select a number from the dropdown menu. - 15 Is Selected

Or Please select the number of each type of institution that uses the PRS account "... 1d. Teaching hospital(s) - Select a number from the dropdown menu. - 16 Is Selected

Or Please select the number of each type of institution that uses the PRS account "... 1d. Teaching hospital(s) - Select a number from the dropdown menu. - 17 Is Selected

Or Please select the number of each type of institution that uses the PRS account "... 1d. Teaching hospital(s) - Select a number from the dropdown menu. - 18 Is Selected

Or Please select the number of each type of institution that uses the PRS account "... 1d. Teaching hospital(s) - Select a number from the dropdown menu. - 19 Is Selected

Or Please select the number of each type of institution that uses the PRS account "... 1d. Teaching hospital(s) - Select a number from the dropdown menu. - 20 Is Selected

Or Please select the number of each type of institution that uses the PRS account "... 1d. Teaching hospital(s) - Select a number from the dropdown menu. - 2 Is Selected

Or Please select the number of each type of institution that uses the PRS account "... 1d. Teaching hospital(s) - Select a number from the dropdown menu. - 3 Is Selected

Or Please select the number of each type of institution that uses the PRS account "... 1d. Teaching hospital(s) - Select a number from the dropdown menu. - 4 Is Selected

Or Please select the number of each type of institution that uses the PRS account "... 1d. Teaching hospital(s) - Select a number from the dropdown menu. - 5 Is Selected

Or Please select the number of each type of institution that uses the PRS account "... 1d. Teaching hospital(s) - Select a number from the dropdown menu. - 6 Is Selected

Or Please select the number of each type of institution that uses the PRS account "... 1d. Teaching hospital(s) - Select a number from the dropdown menu. - 7 Is Selected

Or Please select the number of each type of institution that uses the PRS account "... 1d. Teaching hospital(s) - Select a number from the dropdown menu. - 8 Is Selected

Or Please select the number of each type of institution that uses the PRS account "... 1d. Teaching hospital(s) - Select a number from the dropdown menu. - 9 Is Selected

Or Please select the number of each type of institution that uses the PRS account "... 1d. Teaching hospital(s) - Select a number from the dropdown menu. - 10 Is Selected

Or Please select the number of each type of institution that uses the PRS account "... 1d. Teaching hospital(s) - Select a number from the dropdown menu. - 11 Is Selected

Or Please select the number of each type of institution that uses the PRS account "... 1d. Teaching hospital(s) - Select a number from the dropdown menu. - 12 Is Selected

Or Please select the number of each type of institution that uses the PRS account "... 1d. Teaching hospital(s) - Select a number from the dropdown menu. - 13 Is Selected

Or Please select the number of each type of institution that uses the PRS account "... 1d. Teaching hospital(s) - Select a number from the dropdown menu. - 14 Is Selected

Or Please select the number of each type of institution that uses the PRS account "... 1d. Teaching hospital(s) - Select a number from the dropdown menu. - 15 Is Selected

Or Please select the number of each type of institution that uses the PRS account "... 1d. Teaching hospital(s) - Select a number from the dropdown menu. - 16 Is Selected

Or Please select the number of each type of institution that uses the PRS account "... 1d. Teaching hospital(s) - Select a number from the dropdown menu. - 17 Is Selected

Or Please select the number of each type of institution that uses the PRS account "... 1d. Teaching hospital(s) - Select a number from the dropdown menu. - 18 Is Selected

Or Please select the number of each type of institution that uses the PRS account "... 1d. Teaching hospital(s) - Select a number from the dropdown menu. - 19 Is Selected

Or Please select the number of each type of institution that uses the PRS account "... 1d. Teaching hospital(s) - Select a number from the dropdown menu. - 20 Is Selected

Or Please select the number of each type of institution that uses the PRS account "... 1d.Teaching hospital(s) - Select a number from the dropdown menu. - 2 Is Selected

Or Please select the number of each type of institution that uses the PRS account "... 1d.Teaching hospital(s) - Select a number from the dropdown menu. - 3 Is Selected

Or Please select the number of each type of institution that uses the PRS account "... 1d.Teaching hospital(s) - Select a number from the dropdown menu. - 4 Is Selected

Or Please select the number of each type of institution that uses the PRS account "... 1d.Teaching hospital(s) - Select a number from the dropdown menu. - 5 Is Selected

Or Please select the number of each type of institution that uses the PRS account "... 1d.Teaching hospital(s) - Select a number from the dropdown menu. - 6 Is Selected

Or Please select the number of each type of institution that uses the PRS account "... 1d.Teaching hospital(s) - Select a number from the dropdown menu. - 7 Is Selected

Or Please select the number of each type of institution that uses the PRS account "... 1d.Teaching hospital(s) - Select a number from the dropdown menu. - 8 Is Selected

Or Please select the number of each type of institution that uses the PRS account "... 1d.Teaching hospital(s) - Select a number from the dropdown menu. - 9 Is Selected

Or Please select the number of each type of institution that uses the PRS account "... 1d.Teaching hospital(s) - Select a number from the dropdown menu. - 10 Is Selected

Or Please select the number of each type of institution that uses the PRS account "... 1d.Teaching hospital(s) - Select a number from the dropdown menu. - 11 Is Selected

Or Please select the number of each type of institution that uses the PRS account "... 1d.Teaching hospital(s) - Select a number from the dropdown menu. - 12 Is Selected

Or Please select the number of each type of institution that uses the PRS account "... 1d.Teaching hospital(s) - Select a number from the dropdown menu. - 13 Is Selected

Or Please select the number of each type of institution that uses the PRS account "... 1d.Teaching hospital(s) - Select a number from the dropdown menu. - 14 Is Selected

Or Please select the number of each type of institution that uses the PRS account "... 1d.Teaching hospital(s) - Select a number from the dropdown menu. - 15 Is Selected

Or Please select the number of each type of institution that uses the PRS account "... 1d.Teaching hospital(s) - Select a number from the dropdown menu. - 16 Is Selected

Or Please select the number of each type of institution that uses the PRS account "... 1d.Teaching hospital(s) - Select a number from the dropdown menu. - 17 Is Selected

Or Please select the number of each type of institution that uses the PRS account "... 1d.Teaching hospital(s) - Select a number from the dropdown menu. - 18 Is Selected

Or Please select the number of each type of institution that uses the PRS account "... 1d.Teaching hospital(s) - Select a number from the dropdown menu. - 19 Is Selected

Or Please select the number of each type of institution that uses the PRS account "... 1d.Teaching hospital(s) - Select a number from the dropdown menu. - 20 Is Selected

Or Please select the number of teaching hospitals that use the PRS account "... 1d. Teaching hospital(s) - Select a number from the dropdown menu. - 2 Is Selected

Or Please select the number of teaching hospitals that use the PRS account "... 1d. Teaching hospital(s) - Select a number from the dropdown menu. - 3 Is Selected

Or Please select the number of teaching hospitals that use the PRS account "... 1d. Teaching hospital(s) - Select a number from the dropdown menu. - 4 Is Selected

Or Please select the number of teaching hospitals that use the PRS account "... 1d. Teaching hospital(s) - Select a number from the dropdown menu. - 5 Is Selected

Or Please select the number of teaching hospitals that use the PRS account "... 1d. Teaching hospital(s) - Select a number from the dropdown menu. - 6 Is Selected

Or Please select the number of teaching hospitals that use the PRS account "... 1d. Teaching hospital(s) - Select a number from the dropdown menu. - 7 Is Selected

Or Please select the number of teaching hospitals that use the PRS account "... 1d. Teaching hospital(s) - Select a number from the dropdown menu. - 8 Is Selected

Or Please select the number of teaching hospitals that use the PRS account "... 1d. Teaching hospital(s) - Select a number from the dropdown menu. - 9 Is Selected

Or Please select the number of teaching hospitals that use the PRS account "... 1d. Teaching hospital(s) - Select a number from the dropdown menu. - 10 Is Selected

Or Please select the number of teaching hospitals that use the PRS account "... 1d. Teaching hospital(s) - Select a number from the dropdown menu. - 11 Is Selected

Or Please select the number of teaching hospitals that use the PRS account "... 1d. Teaching hospital(s) - Select a number from the dropdown menu. - 12 Is Selected

Or Please select the number of teaching hospitals that use the PRS account "... 1d. Teaching hospital(s) - Select a number from the dropdown menu. - 13 Is Selected

Or Please select the number of teaching hospitals that use the PRS account "... 1d. Teaching hospital(s) - Select a number from the dropdown menu. - 14 Is Selected

Or Please select the number of teaching hospitals that use the PRS account "... 1d. Teaching hospital(s) - Select a number from the dropdown menu. - 15 Is Selected

Or Please select the number of teaching hospitals that use the PRS account "... 1d. Teaching hospital(s) - Select a number from the dropdown menu. - 16 Is Selected

Or Please select the number of teaching hospitals that use the PRS account "... 1d. Teaching hospital(s) - Select a number from the dropdown menu. - 17 Is Selected

Or Please select the number of teaching hospitals that use the PRS account "... 1d. Teaching hospital(s) - Select a number from the dropdown menu. - 18 Is Selected

Or Please select the number of teaching hospitals that use the PRS account "... 1d. Teaching hospital(s) - Select a number from the dropdown menu. - 19 Is Selected

Or Please select the number of teaching hospitals that use the PRS account "... 1d. Teaching hospital(s) - Select a number from the dropdown menu. - 20 Is Selected

Q15 1i. Please select the teaching hospitals or networks (e.g. Mayo Health System) that use the PRS account "${m://ExternalDataReference}". (Check all that apply.)

- Advocate Christ Medical Center
- Advocate Health Care
- Advocate Illinois Masonic Medical Center
- Advocate Lutheran General Hospital
- Akron Children's Hospital
- Akron General Medical Center
- Albany Medical Center Hospital
- Albany Veterans Affairs Medical Center
- Albert Einstein Medical Center (Albert Einstein Healthcare Network)
- All Children's Hospital
- Allegheny General Hospital
- Anne Arundel Medical Center
- Arkansas Children's Hospital
- Atlantic Health
- Augusta University Medical Center
- Augusta Veterans Affairs Medical Center
- Aurora Health Care
- Banner University Medical Center Phoenix
- Banner University Medical Center Tucson Campus
- Bannerhealth
- Barnabas Health
- Barnes Jewish Hospital
- Bassett Healthcare
- Baton Rouge General Medical Center
- Baylor St. Luke’s Medical Center
- Baylor University Medical Center
- Baystate Health System
- Baystate Medical Center
- Beaumont Health
- Beaumont Dearborn
- Beaumont Royal Oak
- Berkshire Medical Center
- Beth Israel Deaconess Medical Center
- Birmingham Veterans Affairs Medical Center
- BJC HealthCare
- Boston Children's Hospital
- Boston Medical Center
- Bridgeport Hospital
- Brigham and Women's Hospital
- Cabell Huntington Hospital
- California Pacific Medical Center
- Cambridge Health Alliance
- Cambridge Health Alliance Cambridge Hospital Campus
- CAMC Health System
- Captain James A. Lovell Federal Health Care Center
- Care New England Health System
- CareGroup Inc.
- Carilion Medical Center
- Carolinas HealthCare System
- Carolinas Medical Center
- Cedars Sinai Medical Center
- Central Arkansas Veterans Healthcare System
- Central Texas Veterans Health Care Sys
- Charleston Area Medical Center
- CHI Health
- CHI Health Creighton University Medical Center Bergan Mercy
- CHI St. Luke’s Health
- Children's Healthcare of Atlanta
- Children's Healthcare of Atlanta (Includes Egleston and Scottish Rite)
- Children's Hospital Los Angeles
- Children's Hospital of Philadelphia
- Children's Medical Center of Dallas
- Children's Memorial Hospital
- Children's Mercy Hospital
- Children's National Medical Center
- Christiana Care Health System
- Cincinnati Children's Hospital Medical Center
- City of Hope National Medical Center
- Cleveland Clinic Foundation
- Cleveland Clinic Health System
- Community Health Network
- Community Regional Medical Center
- Cooper University Hospital
- Dana Farber Cancer Institute
- Danbury Hospital
- Dartmouth Hitchcock Alliance
- Dartmouth Hitchcock Medical Center
- Dayton Veterans Affairs Medical Center
- Denver Health and Hospital Authority
- Department of Veterans Affairs Veterans Integrated Service Network VISN 1
- Department of Veterans Affairs Veterans Integrated Service Network VISN 10
- Department of Veterans Affairs Veterans Integrated Service Network VISN 11
- Department of Veterans Affairs Veterans Integrated Service Network VISN 12
- Department of Veterans Affairs Veterans Integrated Service Network VISN 17
- Department of Veterans Affairs Veterans Integrated Service Network VISN 18
- Department of Veterans Affairs Veterans Integrated Service Network VISN 2
- Department of Veterans Affairs Veterans Integrated Service Network VISN 22
- Department of Veterans Affairs Veterans Integrated Service Network VISN 23
- Department of Veterans Affairs Veterans Integrated Service Network VISN 4
- Department of Veterans Affairs Veterans Integrated Service Network VISN 5
- Department of Veterans Affairs Veterans Integrated Service Network VISN 6
- Department of Veterans Affairs Veterans Integrated Service Network VISN 7
- Department of Veterans Affairs Veterans Integrated Service Network VISN 8
- Department of Veterans Affairs Veterans Integrated Service Network VISN 9
- Dignity Health
- Duke University Health System
- Duke University Hospital
- Durham Veterans Affairs Medical Center
- Edward W. Sparrow Hospital
- Emory Healthcare
- Emory University Hospital
- Emory University Hospital Midtown
- Erlanger Health System
- Erlanger Medical Center
- Eskenazi Health
- Fairview Health Services
- Florida Hospital Orlando
- Franklin Square Hospital
- Froedtert Hospital
- Geisinger Medical Center
- General Health System
- George Washington University Hospital
- Georgetown University Hospital
- Good Samaritan Hospital (TriHealth)
- Grady Memorial Hospital Atlanta GA
- Grant Riverside Methodist Hospitals Grant Medical Center Campus
- Grant Riverside Methodist Hospitals Riverside Campus
- Greenville Health System
- Greenwich Hospital
- Gundersen Health System
- Gundersen Lutheran Medical Center
- Gwinnett Medical Center Lawrenceville
- Hackensack University Medical Center
- Harbor UCLA Medical Center
- Harborview Medical Center
- HealthPartners Inc.
- Hennepin County Medical Center
- Henry Ford Health System
- Henry Ford Hospital
- HonorHealth Scottsdale Osborn Medical Center
- Hospital for Special Surgery
- Hospital of the University of Pennsylvania
- Houston Methodist
- Houston Methodist Hospital
- Howard University Hospital
- Hunter Holmes McGuire Veterans Affairs Medical Center
- Hurley Medical Center
- Indiana University Health Methodist Hospital
- Indiana University Health Inc
- INOVA Fairfax Hospital
- INOVA Health System
- Iowa City Veterans Affairs Medical Center
- Jackson Memorial Hospital
- James J. Peters VA Medical Center
- Jefferson Health System
- Jerry L. Pettis Memorial Veterans Affair Medical Center
- Jersey Shore University Medical Center
- Jesse Brown VA Medical Center
- John H. Stroger Jr. Hospital of Cook County
- John Peter Smith Hospital (Tarrant County Hospital District)
- Johns Hopkins Bayview Medical Center
- Johns Hopkins Health System
- Johns Hopkins Hospital
- Johnson City Medical Center
- Kaiser Foundation Hospital Los Angeles
- Kaiser Foundation Hospitals Northern California
- Kaiser Permanente Foundation Hospitals Southern California
- Keck Medical Center of USC
- KentuckyOne Health University of Louisville Hospital
- Kettering Medical Center
- Lahey Hospital and Medical Center
- Lankenau Hospital
- LeBonheur Children's Hospital
- Lehigh Valley Health Network
- Lehigh Valley Hospital Cedar Crest
- Lenox Hill Healthcare Network
- Lenox Hill Hospital
- Lifespan Inc.
- Loma Linda University Medical Center
- Long Beach Memorial Medical Center
- Long Island Jewish Medical Center
- Louis Stokes Veterans Affairs Medical Center
- Loyola University Health System
- Loyola University Medical Center
- MacNeal Hospital
- Maimonides Medical Center
- Main Line Health
- Maine Medical Center
- Maricopa Medical Center
- Mary Imogene Bassett Hospital
- Massachusetts General Hospital
- Mayo Clinic Hospital Phoenix AZ
- Mayo Clinic Hospital Jacksonville FL
- Mayo Clinic Hospital Rochester MN
- Mayo Health System
- MCG Health Inc dba Augusta University Health Inc.
- McLaren Healthcare Corporation
- McLaren Regional Medical Center
- Medical Center Hospital
- Medical Center Navicent Health
- Medical University of South Carolina Medical Center
- MedStar Health
- Memorial Health System
- Memorial Health University Medical Center
- Memorial Health Inc.
- Memorial Hermann Texas Medical Center
- Memorial Hospital of Rhode Island
- Memorial Medical Center
- Memorial Sloan Kettering Cancer Center
- Mercy Hospital St. Louis
- Meridian Health System
- Methodist Healthcare University Hospital
- MetroHealth Medical Center
- MetroHealth System
- Miami Children's Hospital
- Michael E. DeBakey Veterans Affairs Medical Center
- Minneapolis Veterans Affairs Medical Center
- Miriam Hospital
- Monmouth Medical Center
- Montefiore Medical Center
- Morristown Memorial Hospital
- Mount Auburn Hospital
- Mount Sinai Beth Israel
- Mount Sinai Health System
- Mount Sinai Hospital
- Mount Sinai Medical Center
- Mountain States Health Alliance
- Mt. Sinai St. Luke's/Roosevelt
- National Jewish Health
- Navicent Health
- New Mexico Veterans Affairs Medical Center
- New York Eye and Ear Infirmary of Mount Sinai
- New York Methodist Hospital
- New York Presbyterian Hospital
- New York Presbyterian Healthcare System
- Newark Beth Israel Medical Center
- North Shore University Hospital
- North Shore Long Island Jewish Health System
- NorthShore University Health System Evanston Hospital
- Northwestern Memorial Hospital
- Norwalk Hospital
- NYU Hospitals Center
- NYU Langone Medical Center
- Ochsner Clinic Foundation
- Ohio State University Health System
- OhioHealth
- Oklahoma City Veterans Affairs Medical Center
- Oregon Health & Science University
- Orlando VA Medical Center
- OSF HealthCare System
- OSF Saint Francis Medical Center
- OU Medical Center
- Our Lady of The Lake Regional Medical Center
- Overlook Medical Center
- Palmetto Health
- Palmetto Health Alliance
- Parkland Health & Hospital System
- Partners HealthCare System Inc.
- Penn State Hershey Medical Center
- Philadelphia Veterans Affairs Medical Center
- Phoenix VA Health Care System
- Portland Veterans Affairs Medical Center
- Ralph H. Johnson Veterans Affairs Medical Center
- Reading Hospital and Medical Center
- Regions Hospital
- Rehabilitation Institute of Chicago
- Rhode Island Hospital
- Richard L. Roudebush Veterans Affairs Medical Center
- Robert Wood Johnson Health System
- Robert Wood Johnson University Hospital
- Roger Williams Medical Center
- Ronald Reagan UCLA Medical Center
- Roswell Park Cancer Institute
- Rush System for Health
- Rush University Medical Center
- Saint Barnabas Medical Center
- Saint Francis Care
- Saint Francis Hospital and Medical Center
- Saint Louis University Hospital
- Saint Luke's Health System
- Saint Luke's Hospital of Kansas City
- Saint Peter's University Hospital
- Salem Veterans Affairs Medical Center
- San Francisco General Hospital and Medical Center
- Sanford Medical Center
- Sanford USD Medical Center
- Scott & White Hospital Temple
- Scripps Green Hospital
- Scripps Health
- Seattle Children's Hospital
- Sentara Norfolk General Hospital
- Sinai Health System
- Sinai Samaritan Medical Center
- Sioux Falls VA Health Care System
- Sioux Valley Hospitals and Health System
- Southeast Louisiana Veterans Health Care System
- Southern Arizona Veterans Affairs Health Care System
- Spectrum Health
- Spectrum Health Butterworth Hospital
- St. Christopher's Hospital for Children
- St. Elizabeth's Medical Center
- St. John Health System
- St. John Hospital and Medical Center
- St. Joseph Mercy Hospital
- St. Joseph's Hospital & Medical Center
- St. Louis Children's Hospital
- St. Luke's Hospital
- St. Luke's Medical Center
- St. Vincent Indianapolis Hospital
- Stanford Health Care
- Staten Island University Hospital
- Steward Health Care System
- Stony Brook University Hospital
- Strong Health System
- Strong Memorial Hospital
- Summa Akron City Hospital
- SUNY Downstate Medical Center/University Hospital of Brooklyn
- Syracuse Veterans Affairs Medical Center
- Tampa General Hospital
- Temple University Health System
- Temple University Hospital
- Tenet Health System Hahnemann University Hospital
- Tenet Healthcare Corporation
- Texas Children's Hospital
- The Mount Sinai Hospital
- The National Institutes of Health (NIH) Clinical Center
- The Nebraska Medical Center
- The University of Texas MD Anderson Cancer Center
- Thomas Jefferson University Hospital
- Trinity Health
- Truman Medical Center Hospital Hill
- Tufts Medical Center
- Tulane Medical Center
- UAB Health System University of Alabama at Birmingham
- UC Health
- UCLA Health
- UCSD Healthcare
- UCSF Medical Center
- UF Health Jacksonville
- UF Shands Hospital
- UMass Memorial Medical Center
- UNC Health Care System
- United Health Services Hospitals
- UnityPoint Health Des Moines
- Univ of Connecticut Health Center/John Dempsey Hospital
- Universal Health Services Inc. George Washington University Hospital
- University Health System
- University Hospital SUNY Upstate Medical University
- University Hospitals and Clinics/ University of Mississippi Medical Center
- University Hospitals Case Medical Center
- University Hospitals HealthSystem
- University Medical Center New Orleans
- University of Alabama Hospital
- University of Arkansas for Medical Sciences
- University of California Davis Health System
- University of California Irvine Medical Center
- University of California San Diego Medical Center
- University of Chicago Hospitals and Health System
- University of Chicago Medical Center
- University of Cincinnati Medical Center
- University of Colorado Health
- University of Colorado Hospital
- University of Illinois at Chicago Medical Center
- University of Iowa Hospitals and Clinics
- University of Kansas Hospital
- University of Kentucky Hospital
- University of Maryland Medical Center
- University of Maryland Medical System
- University of Miami Hospital
- University of Michigan Health System
- University of Michigan Medical Center
- University of Minnesota Medical Center Fairview
- University of Missouri Hospitals and Clinics
- University of New Mexico Hospital
- University of North Carolina Hospitals
- University of Pennsylvania Health System
- University of South Alabama Medical Center
- University of Tennessee Medical Center
- University of Texas Health Science Center at Tyler
- University of Toledo Medical Center
- University of Utah Health System
- University of Utah Hospital
- University of Vermont Medical Center
- University of Virginia Medical Center
- University of Washington Medical Center
- University of Wisconsin Hospital and Clinics
- UPMC
- UPMC Hamot
- UPMC Presbyterian Shadyside
- USC Norris Cancer Hospital
- UT Southwestern Medical Center
- UW Medicine
- VA Caribbean Healthcare System
- VA Connecticut Health Care System
- VA Nebraska Western Iowa Health Care System Omaha Division
- VA Sierra Pacific Network (10N21) Department of Veterans Affairs VISN 21
- Valley Children's Healthcare
- Vanderbilt University Medical Center
- Vanguard Health System
- VCU Medical Center
- Veterans Affairs Ann Arbor Healthcare System
- Veterans Affairs Boston Healthcare System
- Veterans Affairs Greater Los Angeles Health Care System
- Veterans Affairs Long Beach Healthcare System
- Veterans Affairs Maryland Health Care System
- Veterans Affairs Medical and Regional Office Center
- Veterans Affairs Medical Center (Atlanta)
- Veterans Affairs Medical Center James A. Haley Veterans Hospital
- Veterans Affairs Medical Center Memphis Tennessee
- Veterans Affairs New York Harbor Health Care System New York Campus
- Veterans Affairs Pittsburgh Healthcare System
- Veterans Affairs Puget Sound Health Care System
- Veterans Affairs San Diego Healthcare System
- Veterans Affairs South Texas Health Care System
- Veterans Affairs Tennessee Valley Health Care System
- Veterans Affairs Western New York Healthcare System
- Vidant Health
- Vidant Medical Center
- Wake Forest Baptist Medical Center
- Washington Hospital Center
- Wellspan Health
- Wellspan York Hospital
- Wellstar Atlanta Medical Center
- West Kendall Baptist Hospital
- West Penn Allegheny Health System
- West Virginia United Health System
- West Virginia University Hospitals Inc.
- Westchester Medical Center
- Western Pennsylvania Hospital
- Wexner Medical Center at The Ohio State University
- White Memorial Medical Center
- White River Junction VA Medical Center
- William Jennings Bryan Dorn VA Medical Center
- Winthrop South Nassau University Health System Inc.
- Winthrop University Hospital
- Women and Infants Hospital of Rhode Island
- Yale New Haven Health System
- Yale New Haven Hospital
- Other (please specify)

Display This Question:

If 1h. Please select the teaching hospital or network (e.g. Mayo Health Network) that uses the PRS a... Other (please specify) Is Selected

Or 1i. Please select the teaching hospitals or networks (e.g. Mayo Health System) that use the PRS account "${m://ExternalDataReference}". (Check all that apply.) Other Is Selected

Q143 1j. Please specify which other teaching hospital or network uses the PRS account "${m://ExternalDataReference}".

Display This Question:

If Please select the number of each type of institution that uses the PRS account "... 1e. School of medicine - Select a number from the dropdown menu. - 1 Is Selected

Or Please select the number of each type of institution that uses the PRS account "... 1e. School of medicine - Select a number from the dropdown menu. - 1 Is Selected

Or Please select the number of each type of institution that uses the PRS account "... 1e. School of medicine - Select a number from the dropdown menu. - 1 Is Selected

Or Please select the number of schools of medicine that use the PRS account "... 1e. School of medicine - Select a number from the dropdown menu. - 1 Is Selected

Q16 1k. Please select the medical school that uses the PRS account "${m://ExternalDataReference}".

- Albany Medical College
- Albert Einstein College of Medicine
- Baylor College of Medicine
- Boston University School of Medicine
- California Northstate University College of Medicine
- Case Western Reserve University School of Medicine
- Central Michigan University College of Medicine
- Charles E. Schmidt College of Medicine at Florida Atlantic University
- Chicago Medical School at Rosalind Franklin University of Medicine & Science
- Columbia University College of Physicians and Surgeons
- Cooper Medical School of Rowan University
- Creighton University School of Medicine
- CUNY School of Medicine
- Drexel University College of Medicine
- Duke University School of Medicine
- East Tennessee State University James H. Quillen College of Medicine
- Eastern Virginia Medical School
- Emory University School of Medicine
- Florida International University Herbert Wertheim College of Medicine
- Florida State University College of Medicine
- Frank H. Netter MD School of Medicine at Quinnipiac University
- Geisel School of Medicine at Dartmouth
- George Washington University School of Medicine and Health Sciences
- Georgetown University School of Medicine
- Harvard Medical School
- Hofstra Northwell School of Medicine at Hofstra University
- Howard University College of Medicine
- Icahn School of Medicine at Mount Sinai
- Indiana University School of Medicine
- Jacobs School of Medicine and Biomedical Sciences at the University at Buffalo
- Johns Hopkins University School of Medicine
- Keck School of Medicine of the University of Southern California
- Lewis Katz School of Medicine at Temple University
- Loma Linda University School of Medicine
- Louisiana State University School of Medicine in New Orleans
- Louisiana State University School of Medicine in Shreveport
- Loyola University Chicago Stritch School of Medicine
- Marshall University Joan C. Edwards School of Medicine
- Mayo Medical School
- McGovern Medical School at the University of Texas Health Science Center at Houston
- Medical College of Georgia at Augusta University
- Medical College of Wisconsin
- Medical University of South Carolina College of Medicine
- Meharry Medical College
- Mercer University School of Medicine
- Michigan State University College of Human Medicine
- Morehouse School of Medicine
- New York Medical College
- New York University School of Medicine
- Northeast Ohio Medical University
- Northwestern University Feinberg School of Medicine
- Oakland University William Beaumont School of Medicine
- Ohio State University College of Medicine
- Oregon Health & Science University School of Medicine
- Pennsylvania State University College of Medicine
- Perelman School of Medicine at the University of Pennsylvania
- Ponce Health Sciences University School of Medicine
- Rush Medical College of Rush University Medical Center
- Rutgers New Jersey Medical School
- Rutgers Robert Wood Johnson Medical School
- Saint Louis University School of Medicine
- San Juan Bautista School of Medicine
- Sidney Kimmel Medical College at Thomas Jefferson University
- Southern Illinois University School of Medicine
- Stanford University School of Medicine
- State University of New York Downstate Medical Center College of Medicine
- State University of New York Upstate Medical University
- Stony Brook University School of Medicine
- Texas A&M Health Science Center College of Medicine
- Texas Tech University Health Sciences Center School of Medicine
- The Brody School of Medicine at East Carolina University
- The Commonwealth Medical College
- The University of Toledo College of Medicine
- The Warren Alpert Medical School of Brown University
- Tufts University School of Medicine
- Tulane University School of Medicine
- Uniformed Services University of the Health Sciences F. Edward Hebert School of Medicine
- Universidad Central del Caribe School of Medicine
- University of Alabama School of Medicine
- University of Arizona College of Medicine
- University of Arizona College of Medicine Phoenix
- University of Arkansas for Medical Sciences College of Medicine
- University of California Davis School of Medicine
- University of California Irvine School of Medicine
- University of California Los Angeles David Geffen School of Medicine
- University of California Riverside School of Medicine
- University of California Riverside School of Medicine
- University of California San Diego School of Medicine
- University of California San Francisco School of Medicine
- University of Central Florida College of Medicine
- University of Chicago Division of the Biological Sciences The Pritzker School of Medicine
- University of Cincinnati College of Medicine
- University of Colorado School of Medicine
- University of Connecticut School of Medicine
- University of Florida College of Medicine
- University of Hawaii John A. Burns School of Medicine
- University of Illinois College of Medicine
- University of Iowa Roy J. and Lucille A. Carver College of Medicine
- University of Kansas School of Medicine
- University of Kentucky College of Medicine
- University of Louisville School of Medicine
- University of Maryland School of Medicine
- University of Massachusetts Medical School
- University of Miami Leonard M. Miller School of Medicine
- University of Michigan Medical School
- University of Minnesota Medical School
- University of Mississippi School of Medicine
- University of Missouri Columbia School of Medicine
- University of Missouri Kansas City School of Medicine
- University of Nebraska College of Medicine
- University of Nevada School of Medicine
- University of New Mexico School of Medicine
- University of North Carolina at Chapel Hill School of Medicine
- University of North Dakota School of Medicine and Health Sciences
- University of Oklahoma College of Medicine
- University of Pittsburgh School of Medicine
- University of Puerto Rico School of Medicine
- University of Rochester School of Medicine and Dentistry
- University of South Alabama College of Medicine
- University of South Carolina School of Medicine
- University of South Carolina School of Medicine Greenville
- University of South Dakota Sanford School of Medicine
- University of Tennessee Health Science Center College of Medicine
- University of Texas at Austin Dell Medical School
- University of Texas Medical Branch School of Medicine
- University of Texas Rio Grande Valley School of Medicine
- University of Texas School of Medicine at San Antonio
- University of Texas Southwestern Medical Center Southwestern Medical School
- University of Utah School of Medicine
- University of Vermont College of Medicine
- University of Virginia School of Medicine
- University of Washington School of Medicine
- University of Wisconsin School of Medicine and Public Health
- USF Health Morsani College of Medicine
- Vanderbilt University School of Medicine
- Virginia Commonwealth University School of Medicine
- Virginia Tech Carilion School of Medicine
- Wake Forest School of Medicine of Wake Forest Baptist Medical Center
- Washington University in St. Louis School of Medicine
- Wayne State University School of Medicine
- Weill Cornell Medicine
- West Virginia University School of Medicine
- Western Michigan University Homer Stryker M.D. School of Medicine
- Wright State University Boonshoft School of Medicine
- Yale School of Medicine

Display This Question:

If Please select the number of each type of institution that uses the PRS account "... 1e. School of medicine - Select a number from the dropdown menu. - 2 Is Selected

Or Please select the number of each type of institution that uses the PRS account "... 1e. School of medicine - Select a number from the dropdown menu. - 3 Is Selected

Or Please select the number of each type of institution that uses the PRS account "... 1e. School of medicine - Select a number from the dropdown menu. - 4 Is Selected

Or Please select the number of each type of institution that uses the PRS account "... 1e. School of medicine - Select a number from the dropdown menu. - 5 Is Selected

Or Please select the number of each type of institution that uses the PRS account "... 1e. School of medicine - Select a number from the dropdown menu. - 6 Is Selected

Or Please select the number of each type of institution that uses the PRS account "... 1e. School of medicine - Select a number from the dropdown menu. - 7 Is Selected

Or Please select the number of each type of institution that uses the PRS account "... 1e. School of medicine - Select a number from the dropdown menu. - 8 Is Selected

Or Please select the number of each type of institution that uses the PRS account "... 1e. School of medicine - Select a number from the dropdown menu. - 9 Is Selected

Or Please select the number of each type of institution that uses the PRS account "... 1e. School of medicine - Select a number from the dropdown menu. - 10 Is Selected

Or Please select the number of each type of institution that uses the PRS account "... 1e. School of medicine - Select a number from the dropdown menu. - 11 Is Selected

Or Please select the number of each type of institution that uses the PRS account "... 1e. School of medicine - Select a number from the dropdown menu. - 12 Is Selected

Or Please select the number of each type of institution that uses the PRS account "... 1e. School of medicine - Select a number from the dropdown menu. - 13 Is Selected

Or Please select the number of each type of institution that uses the PRS account "... 1e. School of medicine - Select a number from the dropdown menu. - 14 Is Selected

Or Please select the number of each type of institution that uses the PRS account "... 1e. School of medicine - Select a number from the dropdown menu. - 15 Is Selected

Or Please select the number of each type of institution that uses the PRS account "... 1e. School of medicine - Select a number from the dropdown menu. - 16 Is Selected

Or Please select the number of each type of institution that uses the PRS account "... 1e. School of medicine - Select a number from the dropdown menu. - 17 Is Selected

Or Please select the number of each type of institution that uses the PRS account "... 1e. School of medicine - Select a number from the dropdown menu. - 18 Is Selected

Or Please select the number of each type of institution that uses the PRS account "... 1e. School of medicine - Select a number from the dropdown menu. - 19 Is Selected

Or Please select the number of each type of institution that uses the PRS account "... 1e. School of medicine - Select a number from the dropdown menu. - 20 Is Selected

Or Please select the number of each type of institution that uses the PRS account "... 1e. School of medicine - Select a number from the dropdown menu. - 2 Is Selected

Or Please select the number of each type of institution that uses the PRS account "... 1e. School of medicine - Select a number from the dropdown menu. - 3 Is Selected

Or Please select the number of each type of institution that uses the PRS account "... 1e. School of medicine - Select a number from the dropdown menu. - 4 Is Selected

Or Please select the number of each type of institution that uses the PRS account "... 1e. School of medicine - Select a number from the dropdown menu. - 2 Is Selected

Or Please select the number of each type of institution that uses the PRS account "... 1e. School of medicine - Select a number from the dropdown menu. - 3 Is Selected

Or Please select the number of each type of institution that uses the PRS account "... 1e. School of medicine - Select a number from the dropdown menu. - 4 Is Selected

Or Please select the number of each type of institution that uses the PRS account "... 1e. School of medicine - Select a number from the dropdown menu. - 5 Is Selected

Or Please select the number of each type of institution that uses the PRS account "... 1e. School of medicine - Select a number from the dropdown menu. - 6 Is Selected

Or Please select the number of each type of institution that uses the PRS account "... 1e. School of medicine - Select a number from the dropdown menu. - 7 Is Selected

Or Please select the number of each type of institution that uses the PRS account "... 1e. School of medicine - Select a number from the dropdown menu. - 8 Is Selected

Or Please select the number of each type of institution that uses the PRS account "... 1e. School of medicine - Select a number from the dropdown menu. - 9 Is Selected

Or Please select the number of each type of institution that uses the PRS account "... 1e. School of medicine - Select a number from the dropdown menu. - 10 Is Selected

Or Please select the number of each type of institution that uses the PRS account "... 1e. School of medicine - Select a number from the dropdown menu. - 11 Is Selected

Or Please select the number of each type of institution that uses the PRS account "... 1e. School of medicine - Select a number from the dropdown menu. - 12 Is Selected

Or Please select the number of each type of institution that uses the PRS account "... 1e. School of medicine - Select a number from the dropdown menu. - 13 Is Selected

Or Please select the number of each type of institution that uses the PRS account "... 1e. School of medicine - Select a number from the dropdown menu. - 14 Is Selected

Or Please select the number of each type of institution that uses the PRS account "... 1e. School of medicine - Select a number from the dropdown menu. - 15 Is Selected

Or Please select the number of each type of institution that uses the PRS account "... 1e. School of medicine - Select a number from the dropdown menu. - 16 Is Selected

Or Please select the number of each type of institution that uses the PRS account "... 1e. School of medicine - Select a number from the dropdown menu. - 17 Is Selected

Or Please select the number of each type of institution that uses the PRS account "... 1e. School of medicine - Select a number from the dropdown menu. - 18 Is Selected

Or Please select the number of each type of institution that uses the PRS account "... 1e. School of medicine - Select a number from the dropdown menu. - 19 Is Selected

Or Please select the number of each type of institution that uses the PRS account "... 1e. School of medicine - Select a number from the dropdown menu. - 20 Is Selected

Or Please select the number of schools of medicine that use the PRS account "... 1e. School of medicine - Select a number from the dropdown menu. - 2 Is Selected

Or Please select the number of schools of medicine that use the PRS account "... 1e. School of medicine - Select a number from the dropdown menu. - 3 Is Selected

Or Please select the number of schools of medicine that use the PRS account "... 1e. School of medicine - Select a number from the dropdown menu. - 4 Is Selected

Q17 1l. Please select the medical schools that use the PRS account "${m://ExternalDataReference}". (Check all that apply.)

- Albany Medical College
- Albert Einstein College of Medicine
- Baylor College of Medicine
- Boston University School of Medicine
- California Northstate University College of Medicine
- Case Western Reserve University School of Medicine
- Central Michigan University College of Medicine
- Charles E. Schmidt College of Medicine at Florida Atlantic University
- Chicago Medical School at Rosalind Franklin University of Medicine & Science
- Columbia University College of Physicians and Surgeons
- Cooper Medical School of Rowan University
- Creighton University School of Medicine
- CUNY School of Medicine
- Drexel University College of Medicine
- Duke University School of Medicine
- East Tennessee State University James H. Quillen College of Medicine
- Eastern Virginia Medical School
- Emory University School of Medicine
- Florida International University Herbert Wertheim College of Medicine
- Florida State University College of Medicine
- Frank H. Netter MD School of Medicine at Quinnipiac University
- Geisel School of Medicine at Dartmouth
- George Washington University School of Medicine and Health Sciences
- Georgetown University School of Medicine
- Harvard Medical School
- Hofstra Northwell School of Medicine at Hofstra University
- Howard University College of Medicine
- Icahn School of Medicine at Mount Sinai
- Indiana University School of Medicine
- Jacobs School of Medicine and Biomedical Sciences at the University at Buffalo
- Johns Hopkins University School of Medicine
- Keck School of Medicine of the University of Southern California
- Lewis Katz School of Medicine at Temple University
- Loma Linda University School of Medicine
- Louisiana State University School of Medicine in New Orleans
- Louisiana State University School of Medicine in Shreveport
- Loyola University Chicago Stritch School of Medicine
- Marshall University Joan C. Edwards School of Medicine
- Mayo Medical School
- McGovern Medical School at the University of Texas Health Science Center at Houston
- Medical College of Georgia at Augusta University
- Medical College of Wisconsin
- Medical University of South Carolina College of Medicine
- Meharry Medical College
- Mercer University School of Medicine
- Michigan State University College of Human Medicine
- Morehouse School of Medicine
- New York Medical College
- New York University School of Medicine
- Northeast Ohio Medical University
- Northwestern University Feinberg School of Medicine
- Oakland University William Beaumont School of Medicine
- Ohio State University College of Medicine
- Oregon Health & Science University School of Medicine
- Pennsylvania State University College of Medicine
- Perelman School of Medicine at the University of Pennsylvania
- Ponce Health Sciences University School of Medicine
- Rush Medical College of Rush University Medical Center
- Rutgers New Jersey Medical School
- Rutgers Robert Wood Johnson Medical School
- Saint Louis University School of Medicine
- San Juan Bautista School of Medicine
- Sidney Kimmel Medical College at Thomas Jefferson University
- Southern Illinois University School of Medicine
- Stanford University School of Medicine
- State University of New York Downstate Medical Center College of Medicine
- State University of New York Upstate Medical University
- Stony Brook University School of Medicine
- Texas A&M Health Science Center College of Medicine
- Texas Tech University Health Sciences Center School of Medicine
- The Brody School of Medicine at East Carolina University
- The Commonwealth Medical College
- The University of Toledo College of Medicine
- The Warren Alpert Medical School of Brown University
- Tufts University School of Medicine
- Tulane University School of Medicine
- Uniformed Services University of the Health Sciences F. Edward Hebert School of Medicine
- Universidad Central del Caribe School of Medicine
- University of Alabama School of Medicine
- University of Arizona College of Medicine
- University of Arizona College of Medicine Phoenix
- University of Arkansas for Medical Sciences College of Medicine
- University of California Davis School of Medicine
- University of California Irvine School of Medicine
- University of California Los Angeles David Geffen School of Medicine
- University of California Riverside School of Medicine
- University of California Riverside School of Medicine
- University of California San Diego School of Medicine
- University of California San Francisco School of Medicine
- University of Central Florida College of Medicine
- University of Chicago Division of the Biological Sciences The Pritzker School of Medicine
- University of Cincinnati College of Medicine
- University of Colorado School of Medicine
- University of Connecticut School of Medicine
- University of Florida College of Medicine
- University of Hawaii John A. Burns School of Medicine
- University of Illinois College of Medicine
- University of Iowa Roy J. and Lucille A. Carver College of Medicine
- University of Kansas School of Medicine
- University of Kentucky College of Medicine
- University of Louisville School of Medicine
- University of Maryland School of Medicine
- University of Massachusetts Medical School
- University of Miami Leonard M. Miller School of Medicine
- University of Michigan Medical School
- University of Minnesota Medical School
- University of Mississippi School of Medicine
- University of Missouri Columbia School of Medicine
- University of Missouri Kansas City School of Medicine
- University of Nebraska College of Medicine
- University of Nevada School of Medicine
- University of New Mexico School of Medicine
- University of North Carolina at Chapel Hill School of Medicine
- University of North Dakota School of Medicine and Health Sciences
- University of Oklahoma College of Medicine
- University of Pittsburgh School of Medicine
- University of Puerto Rico School of Medicine
- University of Rochester School of Medicine and Dentistry
- University of South Alabama College of Medicine
- University of South Carolina School of Medicine
- University of South Carolina School of Medicine Greenville
- University of South Dakota Sanford School of Medicine
- University of Tennessee Health Science Center College of Medicine
- University of Texas at Austin Dell Medical School
- University of Texas Medical Branch School of Medicine
- University of Texas Rio Grande Valley School of Medicine
- University of Texas School of Medicine at San Antonio
- University of Texas Southwestern Medical Center Southwestern Medical School
- University of Utah School of Medicine
- University of Vermont College of Medicine
- University of Virginia School of Medicine
- University of Washington School of Medicine
- University of Wisconsin School of Medicine and Public Health
- USF Health Morsani College of Medicine
- Vanderbilt University School of Medicine
- Virginia Commonwealth University School of Medicine
- Virginia Tech Carilion School of Medicine
- Wake Forest School of Medicine of Wake Forest Baptist Medical Center
- Washington University in St. Louis School of Medicine
- Wayne State University School of Medicine
- Weill Cornell Medicine
- West Virginia University School of Medicine
- Western Michigan University Homer Stryker M.D. School of Medicine
- Wright State University Boonshoft School of Medicine
- Yale School of Medicine

Q18 1m. If the PRS account "${m://ExternalDataReference}" includes records associated with a Clinical and Translational Science Award (CTSA) institution, please select the relevant institution

- Not applicable
- Albert Einstein College of Medicine
- Boston University
- Case Western Reserve University
- Children's National Medical Center
- Columbia University
- Dartmouth College
- Duke University
- Emory University
- Georgetown University with Howard University
- Harvard University
- Indiana University School of Medicine
- Johns Hopkins University
- Mayo Clinic
- Medical College of Wisconsin
- Medical University of South Carolina
- Mount Sinai School of Medicine
- New York University School of Medicine
- Northwestern University
- Ohio State University
- Oregon Health & Science University
- Penn State Milton S. Hershey Medical Center
- Rockefeller University
- Scripps Research Institute
- Stanford University
- Tufts University
- University at Buffalo
- University of Alabama at Birmingham
- University of Arkansas for Medical Sciences
- University of California Los Angeles
- University of California, Davis
- University of California, Irvine
- University of California, San Diego
- University of California, San Francisco
- University of Chicago
- University of Cincinnati
- University of Colorado Denver
- University of Florida
- University of Illinois at Chicago
- University of Iowa
- University of Kansas Medical Center
- University of Kentucky Research Foundations
- University of Massachusetts Medical School Worcester
- University of Miami
- University of Michigan at Ann Arbor
- University of Minnesota Twin Cities
- University of New Mexico Health Sciences Center
- University of North Carolina at Chapel Hill
- University of Pennsylvania
- University of Pittsburgh
- University of Rochester School of Medicine and Dentistry
- University of Southern California
- University of Texas Health Science Center at Houston
- University of Texas Health Science Center at San Antonio
- University of Texas Medical Branch
- University of Texas Southwestern Medical Center at Dallas
- University of Utah
- University of Washington
- University of Wisconsin Madison
- Vanderbilt University
- Virginia Commonwealth University
- Wake Forest Baptist Medical Center
- Washington University
- Weill Cornell Medical College
- Yale University

Q19 Timing

First Click

Last Click

Page Submit

Click Count

Q20 2. For the PRS account "${m://ExternalDataReference}", is there a database or electronic management system for monitoring ClinicalTrials.gov compliance? (Answer "Yes" if you use Access, REDCap, Click IRB, or some other database system to monitor problem records or to notify people about problem records.)

- Yes
- No
- Don't know

Q21 For clinical trials related to the PRS account "${m://ExternalDataReference}", is there a written policy (or policies) regarding:

Q22 3. registration on ClinicalTrials.gov?

- Yes
- No
- Don't know

Q23 4. results reporting on ClinicalTrials.gov?

- Yes
- No
- Don't know

Q24 5. ClinicalTrials.gov records for investigators joining (e.g. hired by) the entity or entities that use the PRS account "${m://ExternalDataReference}"?

- Yes
- No
- Don't know

Q25 6. ClinicalTrials.gov records for investigators leaving the entity or entities that use the PRS account "${m://ExternalDataReference}"?

- Yes
- No
- Don't know

Q26 Timing

First Click

Last Click

Page Submit

Click Count

Q27 7. Including you, how many people are employed to support ClinicalTrials.gov compliance for the PRS account "${m://ExternalDataReference}"?  (Include full-time and part-time employees.)

- 1 (only me)
- 2
- 3
- 4
- 5
- 6
- 7
- 8
- 9
- 10
- Don't know

Q28 When did staff begin to be assigned to support investigators with ClinicalTrials.gov registration and reporting requirements for trials related to the PRS account "${m://ExternalDataReference}" (either part- or full-time)?

| **8a. Year** | **8b. Month** |
| --- | --- |

Q29 9a. Including you, which of the following functions are undertaken by staff who support the PRS account "${m://ExternalDataReference}" with respect to ClinicalTrials.gov? (Check all that apply.)

- Conduct group (e.g. classroom style) training about RESULTS entry
- Conduct group (e.g. classroom style) training about clinical trial REGISTRATION
- Conduct individual (one on one) training about RESULTS entry
- Conduct individual (one on one) training about clinical trial REGISTRATION
- Enter data into ClinicalTrials.gov on behalf of PIs
- Maintain an educational website
- Notify research teams about institutional sanctions for noncompliance
- Notify researchers (e.g. PIs) about FDA sanctions for noncompliance
- Notify researchers (e.g. PIs) about problem records (e.g. "Late Results per FDAAA")
- Provide assistance with statistical analysis (i.e. doctoral or masters level analysis)
- Respond to questions from researchers (e.g. PIs) regarding ClinicalTrials.gov compliance (e.g. by phone or email)
- Review problem records (e.g. "Late Results per FDAAA")
- Other (please specify)
- Don't know

Display This Question:

If 9a. Including you, which of the following functions are undertaken by staff who support the PRS a... Other (please specify) Is Selected

Q30 9b. Please describe the other functions undertaken by staff who support the PRS account "${m://ExternalDataReference}" with respect to ClinicalTrials.gov.

Display This Question:

If 7. Including you, how many people are employed to support ClinicalTrials.gov compliance for the P... 1 (only me) Is Selected

Q31 Including you, describe the staff who support ClinicalTrials.gov compliance for the PRS account "${m://ExternalDataReference}".  Please list their highest degree obtained and percent effort dedicated to ClinicalTrials.gov compliance.  (Enter time as percentage of a full-time employee working 100%.  For example, a half-time worker who spends half of their time on ClinicalTrials.gov compliance dedicates 25% FTE to these tasks.)

|  | Education level (highest degree obtained) | | | | | | Percentage effort spent on ClinicalTrials.gov compliance (please enter an integer value only) |
| --- | --- | --- | --- | --- | --- | --- | --- |
|  | PhD | MD | JD | Masters degree (e.g. MA or MPA) | Bachelor's degree (e.g. BA or AB) | High school diploma | % FTE |
| 10a. Employee 1 (me) |  |  |  |  |  |  |  |

Display This Question:

If 7. Including you, how many people are employed to support ClinicalTrials.gov compliance for the P... 2 Is Selected

Q32 Including you, describe the staff who support ClinicalTrials.gov compliance for the PRS account "${m://ExternalDataReference}".  Please list their highest degree obtained and percent effort dedicated to ClinicalTrials.gov compliance.  (Enter time as percentage of a full-time employee working 100%.  For example, a half-time worker who spends half of their time on ClinicalTrials.gov compliance dedicates 25% FTE to these tasks.)

|  | Education level (highest degree obtained) | | | | | | Percentage effort spent on ClinicalTrials.gov compliance (please enter an integer value only) |
| --- | --- | --- | --- | --- | --- | --- | --- |
|  | PhD | MD | JD | Masters degree (e.g. MA or MPA) | Bachelor's degree (e.g. BA or AB) | High school diploma | % FTE |
| 10a. Employee 1 (me) |  |  |  |  |  |  |  |
| 10b. Employee 2 |  |  |  |  |  |  |  |

Display This Question:

If 7. Including you, how many people are employed to support ClinicalTrials.gov compliance for the P... 3 Is Selected

Q33 Including you, describe the staff who support ClinicalTrials.gov compliance for the PRS account "${m://ExternalDataReference}".  Please list their highest degree obtained and percent effort dedicated to ClinicalTrials.gov compliance.  (Enter time as percentage of a full-time employee working 100%.  For example, a half-time worker who spends half of their time on ClinicalTrials.gov compliance dedicates 25% FTE to these tasks.)

|  | Education level (highest degree obtained) | | | | | | Percentage effort spent on ClinicalTrials.gov compliance (please enter an integer value only) |
| --- | --- | --- | --- | --- | --- | --- | --- |
|  | PhD | MD | JD | Masters degree (e.g. MA or MPA) | Bachelor's degree (e.g. BA or AB) | High school diploma | % FTE |
| 10a. Employee 1 (me) |  |  |  |  |  |  |  |
| 10b. Employee 2 |  |  |  |  |  |  |  |
| 10c. Employee 3 |  |  |  |  |  |  |  |

Display This Question:

If 7. Including you, how many people are employed to support ClinicalTrials.gov compliance for the P... 4 Is Selected

Q34 Including you, describe the staff who support ClinicalTrials.gov compliance for the PRS account "${m://ExternalDataReference}".  Please list their highest degree obtained and percent effort dedicated to ClinicalTrials.gov compliance.  (Enter time as percentage of a full-time employee working 100%.  For example, a half-time worker who spends half of their time on ClinicalTrials.gov compliance dedicates 25% FTE to these tasks.)

|  | Education level (highest degree obtained) | | | | | | Percentage effort spent on ClinicalTrials.gov compliance (please enter an integer value only) |
| --- | --- | --- | --- | --- | --- | --- | --- |
|  | PhD | MD | JD | Masters degree (e.g. MA or MPA) | Bachelor's degree (e.g. BA or AB) | High school diploma | % FTE |
| 10a. Employee 1 (me) |  |  |  |  |  |  |  |
| 10b. Employee 2 |  |  |  |  |  |  |  |
| 10c. Employee 3 |  |  |  |  |  |  |  |
| 10d. Employee 4 |  |  |  |  |  |  |  |

Display This Question:

If 7. Including you, how many people are employed to support ClinicalTrials.gov compliance for the P... 5 Is Selected

Q35 Including you, describe the staff who support ClinicalTrials.gov compliance for the PRS account "${m://ExternalDataReference}".  Please list their highest degree obtained and percent effort dedicated to ClinicalTrials.gov compliance.  (Enter time as percentage of a full-time employee working 100%.  For example, a half-time worker who spends half of their time on ClinicalTrials.gov compliance dedicates 25% FTE to these tasks.)

|  | Education level (highest degree obtained) | | | | | | Percentage effort spent on ClinicalTrials.gov compliance (please enter an integer value only) |
| --- | --- | --- | --- | --- | --- | --- | --- |
|  | PhD | MD | JD | Masters degree (e.g. MA or MPA) | Bachelor's degree (e.g. BA or AB) | High school diploma | % FTE |
| 10a. Employee 1 (me) |  |  |  |  |  |  |  |
| 10b. Employee 2 |  |  |  |  |  |  |  |
| 10c. Employee 3 |  |  |  |  |  |  |  |
| 10d. Employee 4 |  |  |  |  |  |  |  |
| 10e. Employee 5 |  |  |  |  |  |  |  |

Display This Question:

If 7. Including you, how many people are employed to support ClinicalTrials.gov compliance for the P... 6 Is Selected

Q36 Including you, describe the staff who support ClinicalTrials.gov compliance for the PRS account "${m://ExternalDataReference}".  Please list their highest degree obtained and percent effort dedicated to ClinicalTrials.gov compliance.  (Enter time as percentage of a full-time employee working 100%.  For example, a half-time worker who spends half of their time on ClinicalTrials.gov compliance dedicates 25% FTE to these tasks.)

|  | Education level (highest degree obtained) | | | | | | Percentage effort spent on ClinicalTrials.gov compliance (please enter an integer value only) |
| --- | --- | --- | --- | --- | --- | --- | --- |
|  | PhD | MD | JD | Masters degree (e.g. MA or MPA) | Bachelor's degree (e.g. BA or AB) | High school diploma | % FTE |
| 10a. Employee 1 (me) |  |  |  |  |  |  |  |
| 10b. Employee 2 |  |  |  |  |  |  |  |
| 10c. Employee 3 |  |  |  |  |  |  |  |
| 10d. Employee 4 |  |  |  |  |  |  |  |
| 10e. Employee 5 |  |  |  |  |  |  |  |
| 10f. Employee 6 |  |  |  |  |  |  |  |

Display This Question:

If 7. Including you, how many people are employed to support ClinicalTrials.gov compliance for the P... 7 Is Selected

Q37 Including you, describe the staff who support ClinicalTrials.gov compliance for the PRS account "${m://ExternalDataReference}".  Please list their highest degree obtained and percent effort dedicated to ClinicalTrials.gov compliance.  (Enter time as percentage of a full-time employee working 100%.  For example, a half-time worker who spends half of their time on ClinicalTrials.gov compliance dedicates 25% FTE to these tasks.)

|  | Education level (highest degree obtained) | | | | | | Percentage effort spent on ClinicalTrials.gov compliance (please enter an integer value only) |
| --- | --- | --- | --- | --- | --- | --- | --- |
|  | PhD | MD | JD | Masters degree (e.g. MA or MPA) | Bachelor's degree (e.g. BA or AB) | High school diploma | % FTE |
| 10a. Employee 1 (me) |  |  |  |  |  |  |  |
| 10b. Employee 2 |  |  |  |  |  |  |  |
| 10c. Employee 3 |  |  |  |  |  |  |  |
| 10d. Employee 4 |  |  |  |  |  |  |  |
| 10e. Employee 5 |  |  |  |  |  |  |  |
| 10f. Employee 6 |  |  |  |  |  |  |  |
| 10g. Employee 7 |  |  |  |  |  |  |  |

Display This Question:

If 7. Including you, how many people are employed to support ClinicalTrials.gov compliance for the P... 8 Is Selected

Q38 Including you, describe the staff who support ClinicalTrials.gov compliance for the PRS account "${m://ExternalDataReference}".  Please list their highest degree obtained and percent effort dedicated to ClinicalTrials.gov compliance.  (Enter time as percentage of a full-time employee working 100%.  For example, a half-time worker who spends half of their time on ClinicalTrials.gov compliance dedicates 25% FTE to these tasks.)

|  | Education level (highest degree obtained) | | | | | | Percentage effort spent on ClinicalTrials.gov compliance (please enter an integer value only) |
| --- | --- | --- | --- | --- | --- | --- | --- |
|  | PhD | MD | JD | Masters degree (e.g. MA or MPA) | Bachelor's degree (e.g. BA or AB) | High school diploma | % FTE |
| 10a. Employee 1 (me) |  |  |  |  |  |  |  |
| 10b. Employee 2 |  |  |  |  |  |  |  |
| 10c. Employee 3 |  |  |  |  |  |  |  |
| 10d. Employee 4 |  |  |  |  |  |  |  |
| 10e. Employee 5 |  |  |  |  |  |  |  |
| 10f. Employee 6 |  |  |  |  |  |  |  |
| 10g. Employee 7 |  |  |  |  |  |  |  |
| 10h. Employee 8 |  |  |  |  |  |  |  |

Display This Question:

If 7. Including you, how many people are employed to support ClinicalTrials.gov compliance?  9 Is Selected

Q39 Including you, describe the staff who support ClinicalTrials.gov compliance for the PRS account "${m://ExternalDataReference}".  Please list their highest degree obtained and percent effort dedicated to ClinicalTrials.gov compliance.  (Enter time as percentage of a full-time employee working 100%.  For example, a half-time worker who spends half of their time on ClinicalTrials.gov compliance dedicates 25% FTE to these tasks.)

|  | Education level (highest degree obtained) | | | | | | Percentage effort spent on ClinicalTrials.gov compliance (please enter an integer value only) |
| --- | --- | --- | --- | --- | --- | --- | --- |
|  | PhD | MD | JD | Masters degree (e.g. MA or MPA) | Bachelor's degree (e.g. BA or AB) | High school diploma | % FTE |
| 10a. Employee 1 (me) |  |  |  |  |  |  |  |
| 10b. Employee 2 |  |  |  |  |  |  |  |
| 10c. Employee 3 |  |  |  |  |  |  |  |
| 10d. Employee 4 |  |  |  |  |  |  |  |
| 10e. Employee 5 |  |  |  |  |  |  |  |
| 10f. Employee 6 |  |  |  |  |  |  |  |
| 10g. Employee 7 |  |  |  |  |  |  |  |
| 10h. Employee 8 |  |  |  |  |  |  |  |
| 10i. Employee 9 |  |  |  |  |  |  |  |

Display This Question:

If 7. Including you, how many people are employed to support ClinicalTrials.gov compliance?  10 Is Selected

Q40 Including you, describe the staff who support ClinicalTrials.gov compliance for the PRS account "${m://ExternalDataReference}".  Please list their highest degree obtained and percent effort dedicated to ClinicalTrials.gov compliance.  (Enter time as percentage of a full-time employee working 100%.  For example, a half-time worker who spends half of their time on ClinicalTrials.gov compliance dedicates 25% FTE to these tasks.)

|  | Education level (highest degree obtained) | | | | | | Percentage effort spent on ClinicalTrials.gov compliance (please enter an integer value only) |
| --- | --- | --- | --- | --- | --- | --- | --- |
|  | PhD | MD | JD | Masters degree (e.g. MA or MPA) | Bachelor's degree (e.g. BA or AB) | High school diploma | % FTE |
| 10a. Employee 1 (me) |  |  |  |  |  |  |  |
| 10b. Employee 2 |  |  |  |  |  |  |  |
| 10c. Employee 3 |  |  |  |  |  |  |  |
| 10d. Employee 4 |  |  |  |  |  |  |  |
| 10e. Employee 5 |  |  |  |  |  |  |  |
| 10f. Employee 6 |  |  |  |  |  |  |  |
| 10g. Employee 7 |  |  |  |  |  |  |  |
| 10h. Employee 8 |  |  |  |  |  |  |  |
| 10i. Employee 9 |  |  |  |  |  |  |  |
| 10j. Employee 10 |  |  |  |  |  |  |  |

Q41 11a. Does the entity or entities that use the PRS account "${m://ExternalDataReference}" plan to hire additional staff to support ClinicalTrials.gov compliance within the next year?

- Yes
- No
- Don't know

Display This Question:

If 11a. Does the entity or entities that use the PRS account "${m://ExternalDataReference}" plan to... Yes Is Selected

Q42 11b. Do you know how many new full time equivalent staff (FTEs) will be hired (1 FTE = approximately 40 hours per week, which could include a single person or multiple people)?

- Yes
- No

Display This Question:

If 11b. Do you know how many new full time equivalent staff (FTEs) will be hired (1 FTE = approximat... Yes Is Selected

Q43 11c. How many new full time equivalents (FTEs) will be hired?

Q44 12a. Where are staff who support ClinicalTrials.gov compliance for the PRS account "${m://ExternalDataReference}" employed? (For example, specify the office or department.) (Check all that apply.)

- Dedicated ClinicalTrials.gov office
- Clinical Research office
- Clinical Trials office
- Departments (e.g. Psychiatry or Epidemiology)
- Institutional Review Board (IRB)
- Quality Improvement office
- Regulatory Affairs office
- Research Administration office
- Research Compliance office
- Other (please specify)
- Don't know

Display This Question:

If 12a. Where are staff who support ClinicalTrials.gov compliance for the PRS account "... Other (please specify) Is Selected

Q130 12b. Please specify the other office(s) or department(s) that employ staff who support ClinicalTrials.gov compliance for the PRS account "${m://ExternalDataReference}".

Display This Question:

If 1m. If the PRS account "${m://ExternalDataReference}" includes records associated with a Clinical and Translational Science Award (CTSA) institution, please select the relevant institution Not applicable Is Not Selected

Q45 13. Does the Clinical and Translational Science Award (CTSA) provide support for ClinicalTrials.gov registration and reporting?

- Yes
- No
- Don't know

Q46 Timing

First Click

Last Click

Page Submit

Click Count

Display This Question:

If 3. registration on ClinicalTrials.gov? Yes Is Selected

And 1a. What type of institutions use the PRS account "${m://ExternalDataReference}"? (Check all that... q://QID3/SelectedChoicesCount Is Greater Than 1

Or If

If 3. registration on ClinicalTrials.gov? Yes Is Selected

And Please select the number of NCI designated cancer centers that use the PRS account "... 1c. NCI designated cancer center - Select a number from the dropdown menu. - 2 Is Selected

Or Please select the number of NCI designated cancer centers that use the PRS account "... 1c. NCI designated cancer center - Select a number from the dropdown menu. - 3 Is Selected

Or Please select the number of NCI designated cancer centers that use the PRS account "... 1c. NCI designated cancer center - Select a number from the dropdown menu. - 4 Is Selected

Or If

If 3. registration on ClinicalTrials.gov? Yes Is Selected

And Please select the number of teaching hospitals that use the PRS account "... 1d. Teaching hospital(s) - Select a number from the dropdown menu. - 2 Is Selected

Or Please select the number of teaching hospitals that use the PRS account "... 1d. Teaching hospital(s) - Select a number from the dropdown menu. - 3 Is Selected

Or Please select the number of teaching hospitals that use the PRS account "... 1d. Teaching hospital(s) - Select a number from the dropdown menu. - 4 Is Selected

Or Please select the number of teaching hospitals that use the PRS account "... 1d. Teaching hospital(s) - Select a number from the dropdown menu. - 5 Is Selected

Or Please select the number of teaching hospitals that use the PRS account "... 1d. Teaching hospital(s) - Select a number from the dropdown menu. - 6 Is Selected

Or Please select the number of teaching hospitals that use the PRS account "... 1d. Teaching hospital(s) - Select a number from the dropdown menu. - 7 Is Selected

Or Please select the number of teaching hospitals that use the PRS account "... 1d. Teaching hospital(s) - Select a number from the dropdown menu. - 8 Is Selected

Or Please select the number of teaching hospitals that use the PRS account "... 1d. Teaching hospital(s) - Select a number from the dropdown menu. - 9 Is Selected

Or Please select the number of teaching hospitals that use the PRS account "... 1d. Teaching hospital(s) - Select a number from the dropdown menu. - 10 Is Selected

Or Please select the number of teaching hospitals that use the PRS account "... 1d. Teaching hospital(s) - Select a number from the dropdown menu. - 11 Is Selected

Or Please select the number of teaching hospitals that use the PRS account "... 1d. Teaching hospital(s) - Select a number from the dropdown menu. - 12 Is Selected

Or Please select the number of teaching hospitals that use the PRS account "... 1d. Teaching hospital(s) - Select a number from the dropdown menu. - 13 Is Selected

Or Please select the number of teaching hospitals that use the PRS account "... 1d. Teaching hospital(s) - Select a number from the dropdown menu. - 14 Is Selected

Or Please select the number of teaching hospitals that use the PRS account "... 1d. Teaching hospital(s) - Select a number from the dropdown menu. - 15 Is Selected

Or Please select the number of teaching hospitals that use the PRS account "... 1d. Teaching hospital(s) - Select a number from the dropdown menu. - 16 Is Selected

Or Please select the number of teaching hospitals that use the PRS account "... 1d. Teaching hospital(s) - Select a number from the dropdown menu. - 17 Is Selected

Or Please select the number of teaching hospitals that use the PRS account "... 1d. Teaching hospital(s) - Select a number from the dropdown menu. - 18 Is Selected

Or Please select the number of teaching hospitals that use the PRS account "... 1d. Teaching hospital(s) - Select a number from the dropdown menu. - 19 Is Selected

Or Please select the number of teaching hospitals that use the PRS account "... 1d. Teaching hospital(s) - Select a number from the dropdown menu. - 20 Is Selected

Or If

If 3. registration on ClinicalTrials.gov? Yes Is Selected

And Please select the number of schools of medicine that use the PRS account "... 1e. School of medicine - Select a number from the dropdown menu. - 2 Is Selected

Or Please select the number of schools of medicine that use the PRS account "... 1e. School of medicine - Select a number from the dropdown menu. - 3 Is Selected

Or Please select the number of schools of medicine that use the PRS account "... 1e. School of medicine - Select a number from the dropdown menu. - 4 Is Selected

Or If

If 4. results reporting on ClinicalTrials.gov? Yes Is Selected

And 1a. What type of institutions use the PRS account "${m://ExternalDataReference}"? (Check all that... q://QID3/SelectedChoicesCount Is Greater Than 1

Or If

If 4. results reporting on ClinicalTrials.gov? Yes Is Selected

And Please select the number of NCI designated cancer centers that use the PRS account "... 1c. NCI designated cancer center - Select a number from the dropdown menu. - 2 Is Selected

Or Please select the number of NCI designated cancer centers that use the PRS account "... 1c. NCI designated cancer center - Select a number from the dropdown menu. - 3 Is Selected

Or Please select the number of NCI designated cancer centers that use the PRS account "... 1c. NCI designated cancer center - Select a number from the dropdown menu. - 4 Is Selected

Or If

If 4. results reporting on ClinicalTrials.gov? Yes Is Selected

And Please select the number of teaching hospitals that use the PRS account "... 1d. Teaching hospital(s) - Select a number from the dropdown menu. - 2 Is Selected

Or Please select the number of teaching hospitals that use the PRS account "... 1d. Teaching hospital(s) - Select a number from the dropdown menu. - 3 Is Selected

Or Please select the number of teaching hospitals that use the PRS account "... 1d. Teaching hospital(s) - Select a number from the dropdown menu. - 4 Is Selected

Or Please select the number of teaching hospitals that use the PRS account "... 1d. Teaching hospital(s) - Select a number from the dropdown menu. - 5 Is Selected

Or Please select the number of teaching hospitals that use the PRS account "... 1d. Teaching hospital(s) - Select a number from the dropdown menu. - 6 Is Selected

Or Please select the number of teaching hospitals that use the PRS account "... 1d. Teaching hospital(s) - Select a number from the dropdown menu. - 7 Is Selected

Or Please select the number of teaching hospitals that use the PRS account "... 1d. Teaching hospital(s) - Select a number from the dropdown menu. - 8 Is Selected

Or Please select the number of teaching hospitals that use the PRS account "... 1d. Teaching hospital(s) - Select a number from the dropdown menu. - 9 Is Selected

Or Please select the number of teaching hospitals that use the PRS account "... 1d. Teaching hospital(s) - Select a number from the dropdown menu. - 10 Is Selected

Or Please select the number of teaching hospitals that use the PRS account "... 1d. Teaching hospital(s) - Select a number from the dropdown menu. - 11 Is Selected

Or Please select the number of teaching hospitals that use the PRS account "... 1d. Teaching hospital(s) - Select a number from the dropdown menu. - 12 Is Selected

Or Please select the number of teaching hospitals that use the PRS account "... 1d. Teaching hospital(s) - Select a number from the dropdown menu. - 13 Is Selected

Or Please select the number of teaching hospitals that use the PRS account "... 1d. Teaching hospital(s) - Select a number from the dropdown menu. - 14 Is Selected

Or Please select the number of teaching hospitals that use the PRS account "... 1d. Teaching hospital(s) - Select a number from the dropdown menu. - 15 Is Selected

Or Please select the number of teaching hospitals that use the PRS account "... 1d. Teaching hospital(s) - Select a number from the dropdown menu. - 16 Is Selected

Or Please select the number of teaching hospitals that use the PRS account "... 1d. Teaching hospital(s) - Select a number from the dropdown menu. - 17 Is Selected

Or Please select the number of teaching hospitals that use the PRS account "... 1d. Teaching hospital(s) - Select a number from the dropdown menu. - 18 Is Selected

Or Please select the number of teaching hospitals that use the PRS account "... 1d. Teaching hospital(s) - Select a number from the dropdown menu. - 19 Is Selected

Or Please select the number of teaching hospitals that use the PRS account "... 1d. Teaching hospital(s) - Select a number from the dropdown menu. - 20 Is Selected

Or If

If 4. results reporting on ClinicalTrials.gov? Yes Is Selected

And Please select the number of schools of medicine that use the PRS account "... 1e. School of medicine - Select a number from the dropdown menu. - 2 Is Selected

Or Please select the number of schools of medicine that use the PRS account "... 1e. School of medicine - Select a number from the dropdown menu. - 3 Is Selected

Or Please select the number of schools of medicine that use the PRS account "... 1e. School of medicine - Select a number from the dropdown menu. - 4 Is Selected

Q48 14. Does a single written policy apply to all of the entities that use the PRS account "${m://ExternalDataReference}"? (For example, you would answer "Yes" for an account that includes all records for a University with a written policy that applies to all the schools and divisions. You would answer "No" for a university where the medical school and the school of public health use one PRS account and each school has their own registration policy.)

- Yes
- No
- Don't know

Display This Question:

If 14. Does a single written policy apply to all of the entities that use the PRS account "... No Is Selected

Q49 15. How many separate policies exist for the various entities that use the PRS account "${m://ExternalDataReference}"? (An entity might be a department, a school, or some other part of your institution.)

- 2
- 3
- 4
- 5
- Don't know

Display This Question:

If 14. Does a single written policy apply to all of the entities that use the PRS account "... No Is Selected

Q50 16. Please describe which different entities have registration policies and to whom they apply.

Display This Question:

If 16. Please describe which different entities have registration policies and to whom they apply. Text Response Is Displayed

Q55 17a. Our survey is designed for institutions with a single policy.  Your account sounds more complex.  May we contact you to discuss your institution's policies and procedures?

- Yes
- No

Display This Question:

If 17a. Our survey is designed for institutions with a single policy. Your account sounds more compl... Yes Is Selected

Q56 17b. How would you prefer we contact you?

Display This Question:

If 17b. How would you prefer we contact you? Text Response Is Displayed

Or 17a. Our survey is designed for institutions with a single policy. Your account sounds more compl... No Is Selected

Q57 We appreciate very much your taking the time to complete our survey. We will circulate a summary of results once we have analyzed the data.

If We appreciate very much you... Is Displayed, Then Skip To End of Survey

Display This Question:

If 3. registration on ClinicalTrials.gov? Yes Is Selected

Q47 The following section (Questions 18-30) contains questions about the written registration policy for trials related to the PRS account "${m://ExternalDataReference}".

Display This Question:

If 3. registration on ClinicalTrials.gov? Yes Is Selected

Q51 When did the policy about clinical trials registration come into effect?

| **18a. Year** | **18b. Month** |
| --- | --- |

Display This Question:

If 3. registration on ClinicalTrials.gov? Yes Is Selected

Q53 What types of clinical trials must register or are encouraged to register according to the policy for the PRS account "${m://ExternalDataReference}"?

|  | Required to register | Encouraged to register, not required | Not mentioned in the policy |
| --- | --- | --- | --- |
| 19a. All clinical trials |  |  |  |
| 19b. Clinical trials that include services reimbursed through Medicare or Medicaid (CMS) |  |  |  |
| 19c. Clinical trials meeting ICMJE definition |  |  |  |
| 19d. Clinical trials considered "applicable" under the Food and Drugs Administration Amendments Act (FDAAA) |  |  |  |
| 19e. NIH funded clinical trials |  |  |  |
| 19f. NCI funded clinical trials |  |  |  |
| 19g. Other (please specify) |  |  |  |

Display This Question:

If What types of clinical trials must register or are encouraged to register according to the policy... 19g. Other (please specify) - Required to register Is Selected

Or What types of clinical trials must register or are encouraged to register according to the policy... 19g. Other (please specify) - Encouraged to register, not required Is Selected

Q54 19h. Please describe which other clinical trials either must register or are encouraged to register according to the policy for the PRS account "${m://ExternalDataReference}".

Q58 Timing

First Click

Last Click

Page Submit

Click Count

Display This Question:

If 3. registration on ClinicalTrials.gov? Yes Is Selected

Q59 20a. According to the policy for the PRS account "${m://ExternalDataReference}", who is responsible for determining if a clinical trial must be registered? (Check all that apply.)

- The principal investigator (PI)
- Institutional review board (IRB) staff
- PRS administrator
- Other (please specify)
- Not applicable because this responsibility is not assigned in the policy
- Don't know

Display This Question:

If 20a. According to the policy for the PRS account "${m://ExternalDataReference}", who is responsib... Other (please specify) Is Selected

Q60 20b. Please describe the other entity responsible for determining if a clinical trial must be registered.

Display This Question:

If 20a. According to the policy for the PRS account "${m://ExternalDataReference}", who is responsib... Not applicable because this responsibility is not assigned in the policy Is Selected

Q61 20c. In PRACTICE, who is responsible for determining if a clinical trial must be registered? (Check all that apply.)

- The principal investigator (PI)
- Institutional review board (IRB) staff
- PRS administrator
- Other
- Don't know

Display This Question:

If 3. registration on ClinicalTrials.gov? Yes Is Selected

Q62 21a. According to the policy for the PRS account "${m://ExternalDataReference}", when do clinical trials have to be registered?

- Before the study can be APPROVED by the IRB
- Before the study BEGINS ENROLLMENT
- Within 21 days of starting enrollment
- The requirements differ depending on the type of trial (please explain)
- Not applicable because this is not addressed in the policy
- Don't know

Display This Question:

If 21a. According to the policy for the PRS account "${m://ExternalDataReference}", when do clinical... The requirements differ depending on the type of trial (please explain) Is Selected

Q63 21b. Please explain the different requirements for when clinical trials have to be registered (what each requirement is and to what type of trials it applies).

Display This Question:

If 21a. According to the policy for the PRS account "${m://ExternalDataReference}", when do clinical... Is Selected

Q127 21c. IN PRACTICE, when do trials have to be registered?

- Before the study can be APPROVED by the IRB
- Before the study BEGINS ENROLLMENT
- Within 21 days of starting enrollment
- The requirements differ depending on the type of trial
- Don't know

Display This Question:

If 20a. According to the policy for the PRS account "${m://ExternalDataReference}", who is responsib... Institutional review board (IRB) staff Is Selected

Q64 22. Does the institutional review board (IRB) require registration of a clinical trial prior to initial approval?

- Yes. The IRB requires registration prior to approval for ALL clinical trials
- Yes. The IRB requires registration prior to approval for SOME clinical trials
- No. The IRB does not require registration prior to initial approval for any clinical trials
- Don't know

Display This Question:

If 3. registration on ClinicalTrials.gov? Yes Is Selected

Q65 23a. According to the policy for the PRS account "${m://ExternalDataReference}", who may register a clinical trial on ClinicalTrials.gov? (Check all that apply.)

- The principal investigator (PI)
- Designee of the PI (e.g. member of research team or departmental staff)
- Institutional review board (IRB) staff
- PRS administrator
- Other (please specify)
- Not applicable because this responsibility is not assigned in the policy
- Don't know

Display This Question:

If 23a. According to the policy for the PRS account "${m://ExternalDataReference}", who may register... Other (please specify) Is Selected

Q66 23b. Please describe which other entity may register a clinical trial on ClinicalTrials.gov.

Display This Question:

If 23a. According to the policy for the PRS account "${m://ExternalDataReference}", who may register... Not applicable because this responsibility is not assigned in the policy Is Selected

Q67 23c. In PRACTICE, who may register a clinical trial on ClinicalTrials.gov? (Check all that apply.)

- The principal investigator (PI)
- Designee of the PI (e.g. member of research team or departmental staff)
- Institutional review board (IRB) staff
- PRS administrator
- Other (please specify)
- Don't know

Display This Question:

If 23c. In PRACTICE, who may register a clinical trial on ClinicalTrials.gov? (Check all that apply.) Other (please specify) Is Selected

Q140 23d. Please describe who else may register a trial on ClinicalTrials.gov.

Display This Question:

If 3. registration on ClinicalTrials.gov? Yes Is Selected

Q68 24. Does the registration policy for the PRS account "${m://ExternalDataReference}" indicate that a certain entity is ALWAYS designated the responsible party for all trials?

- Yes
- No
- Don't know

Display This Question:

If 24. Does the registration policy for the PRS account "${m://ExternalDataReference}" indicate that... No Is Selected

Q69 25a. According to the policy for the PRS account "${m://ExternalDataReference}", who MAY BE designated the responsible party? (Check all that apply.)

- The principal investigator (PI) (i.e. an individual "Investigator" is named)
- The sponsor (i.e. the institution is named)
- Not applicable because the policy does not address this issue
- Don't know

Display This Question:

If 25a. According to the policy for the PRS account "${m://ExternalDataReference}", who MAY BE desig... Not applicable because the policy does not address this issue Is Selected

Q128 25b. In PRACTICE, who is designated the responsible party? (Check all that apply.)

- The principal investigator (PI) (i.e. an individual "Investigator" is named)
- The sponsor (i.e. the institution is named)
- Don't know

Display This Question:

If 24. Does the registration policy for the PRS account "${m://ExternalDataReference}" indicate that... Yes Is Selected

Q70 26. Whom does the policy for the PRS account "${m://ExternalDataReference}" indicate is ALWAYS designated the responsible party?

- The principal investigator (PI) (i.e. an individual "Investigator" is named)
- The sponsor (i.e. the institution is named)
- Don't know

Display This Question:

If 3. registration on ClinicalTrials.gov? Yes Is Selected

Q71 27. Does the policy for the PRS account "${m://ExternalDataReference}" indicate that a principal investigator (PI) may be penalized by their institution for failing to register a clinical trial?

- Yes
- No
- Don't know

Display This Question:

If 27. Does the policy for the PRS account "${m://ExternalDataReference}" indicate that a principal... Yes Is Selected

Q72 28a. According to the policy for the PRS account "${m://ExternalDataReference}", which of the following penalties may be applied? (Check all that apply.)

- The principal investigator (PI) may not begin any new research projects
- The principal investigator (PI) may not continue with enrollment on current study
- The principal investigator (PI) may be suspended from work
- The principal investigator (PI) or department may be assessed a monetary penalty
- Other (please specify)
- Don't know

Display This Question:

If 28a. According to the policy for the PRS account "${m://ExternalDataReference}", which of the fol... Other (please specify) Is Selected

Q73 28b. Please describe what other penalties may be applied.

Display This Question:

If 27. Does the policy for the PRS account "${m://ExternalDataReference}" indicate that a principal... Yes Is Selected

Q74 29. Have these penalties EVER been applied?

- Yes
- No
- Don't know

Display This Question:

If 29. Have these penalties EVER been applied? Yes Is Selected

Q75 30. Please describe the circumstances under which these penalties have been applied.

Q76 Timing

First Click

Last Click

Page Submit

Click Count

Display This Question:

If 3. registration on ClinicalTrials.gov? No Is Selected

Q77 The following section (Questions 31-32) contains questions about clinical trials registration practices for the PRS account "${m://ExternalDataReference}" in the absence of an official policy.

Display This Question:

If 3. registration on ClinicalTrials.gov? No Is Selected

Q79 What types of clinical trials are registered in PRACTICE?

|  | Always registered | Sometimes registered | Not registered |
| --- | --- | --- | --- |
| 31a. All clinical trials |  |  |  |
| 31b. Clinical trials that include services reimbursed through Medicare or Medicaid (CMS) |  |  |  |
| 31c. Clinical trials meeting ICMJE definition |  |  |  |
| 31d. Clinical trials considered "applicable" under the Food and Drugs Administration Amendments Act (FDAAA) |  |  |  |
| 31e. NIH funded clinical trials |  |  |  |
| 31f. NCI funded clinical trials |  |  |  |

Display This Question:

If 3. registration on ClinicalTrials.gov? No Is Selected

Q81 32. In PRACTICE, who is usually designated the responsible party for trials registered using this account?

- The principal investigator (PI) (i.e. an individual "Investigator" is named)
- The sponsor (i.e. the institution is named)
- Don't know

Display This Question:

If 4. results reporting on ClinicalTrials.gov? Yes Is Selected

Q82 The following section (Questions 33-42) contains questions about the clinical trials results reporting policy for the PRS account "${m://ExternalDataReference}".

Display This Question:

If 4. results reporting on ClinicalTrials.gov? Yes Is Selected

Q83 33. Is compliance with ClinicalTrials.gov results reporting for the PRS account "${m://ExternalDataReference}" monitored by someone other than investigators themselves?

- Yes
- No
- Don't know

Display This Question:

If 33. Is compliance with ClinicalTrials.gov results reporting for the PRS account "${m://ExternalDataReference}" monitored by someone other than investigators themselves? Yes Is Selected

Q84 34a. Who monitors compliance with results reporting requirements? (Check all that apply.)

- PRS administrator
- Institutional review board (IRB) staff
- Other (please specify)
- Don't know

Display This Question:

If 37a. Who monitors compliance with results reporting requirements? (Check all that apply.) Other (please specify) Is Selected

Q85 34b. Please specify other parties responsible for monitoring compliance with results reporting requirements.

Q86 35a. To your knowledge, has anyone affiliated with the PRS account "${m://ExternalDataReference}" received a warning letter ("Notice") from FDA indicating that their institution could be liable for civil monetary penalties for failing to register or report a clinical trial?

- Yes. A principal investigator (PI) received a warning letter
- Yes. Somebody other than a principal investigator (PI) received a warning letter (e.g. a Dean)
- Yes. Multiple people have received warning letters
- No. To my knowledge nobody has received a warning letter
- Don't know

Display This Question:

If 35a. To your knowledge, has anyone affiliated with the PRS account "${m://ExternalDataReference}" received a warning letter ("Notice") from FDA indicating that their institution could be liable for... Yes. Somebody other than a principal investigator (PI) received a warning letter (e.g. a Dean) Is Selected

Or 35a. To your knowledge, has anyone affiliated with the PRS account "${m://ExternalDataReference}" received a warning letter ("Notice") from FDA indicating that their institution could be liable for... Yes. Multiple people have received warning letters Is Selected

Q87 35b. Please describe who has received warning letter ("Notice") from FDA. (That is, indicate their position / role, NOT their name.)

Q88 Timing

First Click

Last Click

Page Submit

Click Count

Display This Question:

If 4. results reporting on ClinicalTrials.gov? Yes Is Selected

Q134 36a. According to the policy for the PRS account "${m://ExternalDataReference}", who is responsible for ENTERING results for completed clinical trials? (Check all that apply.)

- Principal investigator (PI) or their designee (e.g. member of research team or department staff)
- PRS administrator
- Other (please specify)
- Not applicable because this responsibility is not assigned in the policy
- Don't know

Display This Question:

If 36a. According to the policy for the PRS account "${m://ExternalDataReference}", who is responsib... Other (please specify) Is Selected

Q90 36b. Please describe which other parties are responsible for ENTERING results for completed clinical trials.

Display This Question:

If 36a. According to the policy for the PRS account "${m://ExternalDataReference}", who is responsib... Not applicable because this responsibility is not assigned in the policy Is Selected

Q91 37a. In PRACTICE, who ENTERS results from completed clinical trials? (Check all that apply.)

- The principal investigator (PI) or their designee (e.g. member of research team or departmental staff)
- PRS administrator
- Other (please specify)
- Don't know

Display This Question:

If 37a. In PRACTICE, who ENTERS results from completed clinical trials? (Check all that apply.) Other (please specify) Is Selected

Q92 37b. Please describe who else may ENTER results from a clinical trial on ClinicalTrials.gov.

Display This Question:

If 4. results reporting on ClinicalTrials.gov? Yes Is Selected

Q135 38a. According to the policy for the PRS account "${m://ExternalDataReference}", who is responsible for MONITORING if clinical trial results have been entered on time (e.g. by reviewing "problem records" in the PRS account)? (Check all that apply.)

- The principal investigator (PI)
- Institutional review board (IRB) staff
- PRS administrator
- Other (please specify)
- Not applicable because this responsibility is not assigned in the policy
- Don't know

Display This Question:

If 38a. According to the policy for the PRS account "${m://ExternalDataReference}", who is responsible for MONITORING if clinical trial results have been entered on time (e.g. by reviewing "problem re... Other (please specify) Is Selected

Q94 38b. Please describe what other entity is responsible for MONITORING if clinical trial results have been entered on time.

Display This Question:

If 38a. According to the policy for the PRS account "${m://ExternalDataReference}", who is responsible for MONITORING if clinical trial results have been entered on time (e.g. by reviewing "problem re... Not applicable because this responsibility is not assigned in the policy Is Selected

Q129 38c. In PRACTICE, who is responsible for MONITORING if clinical trial results have been entered on time (e.g. checking problem records or the planning report)? (Check all that apply.)

- The principal investigator (PI)
- Institutional review board (IRB) staff
- PRS administrator
- Other (please specify)
- Don't know

Display This Question:

If 38c. In PRACTICE, who is responsible for MONITORING if clinical trial results have been entered o... Other (please specify) Is Selected

Q136 38d. Please describe who else is responsible for MONITORING if clinical trial results have been entered on time.

Display This Question:

If 4. results reporting on ClinicalTrials.gov? Yes Is Selected

Q95 39. Does the policy for the PRS account "${m://ExternalDataReference}" indicate that a principal investigator (PI) may be penalized by their institution for failing to post results for completed clinical trials?

- Yes
- No
- Don't know

Display This Question:

If 39. Does the policy for the PRS account "${m://ExternalDataReference}" indicate that a principal... Yes Is Selected

Q96 40a. According to the policy for the PRS account "${m://ExternalDataReference}", which of the following penalties may be applied for failing to post results? (Check all that apply.)

- The principal investigator (PI) may not begin any new research projects
- The principal investigator (PI) may be suspended from work
- The principal investigator (PI) or department may be assessed a monetary penalty
- Other (please specify)
- Don't know

Display This Question:

If 40a. According to the policy for the PRS account "${m://ExternalDataReference}", which of the following penalties may be applied for failing to post results? (Check all that apply.) Other (please specify) Is Selected

Q97 40b. Please describe other penalties that may be applied to PIs for failing to post results of completed clinical trials.

Display This Question:

If 39. Does the policy for the PRS account "${m://ExternalDataReference}" indicate that a principal... Yes Is Selected

Q98 41. Have the penalties for failing to post results EVER been applied?

- Yes
- No
- Don't know

Display This Question:

If 41. Have the penalties for failing to post results EVER been applied? Yes Is Selected

Q99 42. Please describe the circumstances under which the penalties for failing to post results have been applied.

Q100 Timing

First Click

Last Click

Page Submit

Click Count

Display This Question:

If 4. results reporting on ClinicalTrials.gov? No Is Selected

Q101 The following question pertains to your institution's clinical trials results reporting practice in the absence of an official policy.

Display This Question:

If 4. results reporting on ClinicalTrials.gov? No Is Selected

Q102 43a. In PRACTICE, who ENTERS results from completed clinical trials?

- The principal investigator (PI) or their designee (e.g. member of research team or department staff)
- PRS administrator
- Other (please specify)
- Don't know

Display This Question:

If 43a. In PRACTICE, who ENTERS results from completed clinical trials? Other (please specify) Is Selected

Q103 43b. Please describe who usually ENTERS results from completed clinical trials on ClinicalTrials.gov.

Display This Question:

If 2. For the PRS account "${m://ExternalDataReference}", is there a database or electronic management system for monitoring ClinicalTrials.gov compliance? (Answer "Yes" if you use Acce... Yes Is Selected

Q104 The following section (Questions 44-49) contains questions about the electronic management system for the PRS account "${m://ExternalDataReference}" for monitoring problem records or notifying people about problem records.

Display This Question:

If 2. For the PRS account "${m://ExternalDataReference}", is there a database or electronic management system for monitoring ClinicalTrials.gov compliance? (Answer "Yes" if you use Acce... Yes Is Selected

Q105 44a. Which of the following systems do you use for monitoring ClinicalTrials.gov compliance? (Check all that apply.)

- REDCap
- Huron ClickIRB
- Forte OnCore
- mdlogix
- System developed in house (e.g. an online system or an Access database). (Please specify.)
- Other (please specify)

Display This Question:

If 44a. Which of the following systems do you use for monitoring ClinicalTrials.gov compliance? (Che... System developed in house (e.g. an online system or an Access database). (Please specify.) Is Selected

Q132 44b. Please describe what system developed in-house (e.g., an online system, an Access database) is used for monitoring ClinicalTrials.gov compliance.

Display This Question:

If 44a. Which of the following systems do you use for monitoring ClinicalTrials.gov compliance? (Che... Other (please specify) Is Selected

Q106 44c. Please describe what other system is used for monitoring ClinicalTrials.gov compliance.

Display This Question:

If 2. For the PRS account "${m://ExternalDataReference}", is there a database or electronic management system for monitoring ClinicalTrials.gov compliance? (Answer "Yes" if you use Access, R... Yes Is Selected

Q107 When was an electronic system first used to monitor compliance for the PRS account "${m://ExternalDataReference}" with ClinicalTrials.gov registration or reporting requirements?

| **45a. Year** | **45b. Month** |
| --- | --- |

Display This Question:

If 2. For the PRS account "${m://ExternalDataReference}", is there a database or electronic manageme... Yes Is Selected

Q108 46. Is this system used to send notifications about problem records (e.g. "Late Results - per FDAAA") or to share comments about a record from ClinicalTrials.gov staff (e.g., "PRS Review Comments")?

- Yes
- No
- Don't know

Display This Question:

If 46. Is this system used to send notifications about problem records (e.g. "Late Results - per FDA... Yes Is Selected

Q109 47. For what problem records does the system send notifications? (Check all that apply.)

- "PRS review comments"
- "Entry not completed"
- "Not recently updated"
- "Record has errors"
- "Missing FDAAA information"
- "Late results per FDAAA"
- "Never released"
- "Update not released"
- "Ready for review and approval"
- Don't know

Display This Question:

If 2. For the PRS account "${m://ExternalDataReference}", is there a database or electronic management system for monitoring ClinicalTrials.gov compliance? (Answer "Yes" if you use Acce... Yes Is Selected

Q142 48. Does the database or electronic management system for monitoring ClinicalTrials.gov compliance for the PRS account "${m://ExternalDataReference}" use an application programming interface (API) to communicate with ClinicalTrials.gov?

- Yes
- No
- Don't know

Q111 Timing

First Click

Last Click

Page Submit

Click Count

Display This Question:

If 2. For the PRS account "${m://ExternalDataReference}", is there a database or electronic management system for monitoring ClinicalTrials.gov compliance? (Answer "Yes" if you use Acce... Yes Is Selected

Q112 49a. ClinicalTrials.gov generates a "Planning Report" for PRS Administrators. Do you use the planning report to identify clinical trials whose results will become due in the near future?

- Yes
- No
- Don't know

Display This Question:

If 49a. ClinicalTrials.gov generates a "Planning Report" for PRS Administrators. Do you use the plan... Yes Is Selected

Q113 49b. Do you use this system to notify people about results deadlines BEFORE they are due?

- Yes
- No
- Don't know

Q114 50a. Does the entity or entities that use the PRS account "${m://ExternalDataReference}" have a program or office specifically dedicated to increasing compliance with ClinicalTrials.gov requirements?

- Yes
- No
- Don't know

Display This Question:

If 50a. Does the entity or entities that use the PRS account "${m://ExternalDataReference}" have a p... Yes Is Selected

Q115 50b. Please describe the program or office (name of the program or office, job title and roles of personnel, number of FTE employees) below.

Q126 51. Please use the space below to describe any aspects of compliance efforts related to the PRS account "${m://ExternalDataReference}" that our questions did not cover, or to provide general feedback about the survey.

Q116 Thank you for completing this survey!  We appreciate your time and will share a summary of results with participants after we complete our analyses.
