# Supplementary material for: Clinical trial registration and reporting: a survey of academic organizations in the United States
Source: BMC Med. 2018 May 2;16:60. doi: 10.1186/s12916-018-1042-6 (PMC5930804; doi:10.1186/s12916-018-1042-6)
Supplement: Supplementary file 2 — Eligible accounts. (DOCX 452 kb) [file 12916_2018_1042_MOESM2_ESM.docx]

**ONLINE SUPPLEMENTS**

**Additional file 2: Eligible accounts**

21st Century Oncology

A.T. Still University of Health Sciences

AAADRS Clinical Research Center

Abdul Latif Jameel Poverty Action Lab

Abramson Cancer Center of the University of Pennsylvania

Academic and Community Cancer Research United

Accelerated Community Oncology Research Network

Accelerated Cure Project for Multiple Sclerosis

Advocate Center for Pediatric Research

Advocate Health Care

Akron Children's Hospital

Akron General Medical Center

Alabama Physical Therapy & Acupuncture

Albany College of Pharmacy and Health Sciences

Albany Medical College

Albert B. Sabin Vaccine Institute

Albert Einstein College of Medicine of Yeshiva University

Albert Einstein Healthcare Network

Allegheny Singer Research Institute

Alliance for Clinical Trials in Oncology

Allina Health System

Alzheimer's Disease Cooperative Study (ADCS)

American Academy of Family Physicians

American Academy of Pediatrics

American Association of Homes and Services for the Aging

American Burn Association

American College of Cardiology

American College of Rheumatology

American Heart Association

American Lung Association Asthma Clinical Research Centers

American Medical Association

American Pharmacists Association Foundation

American Scitech International

American SIDS Institute

American Society Of Thermalism And Climatology Inc

AMyC

Anderson Orthopaedic Research Institute

Ann & Robert H Lurie Children's Hospital of Chicago

Anne Arundel Health System Research Institute

Appalachian State University

Arbor Research Collaborative for Health

Arizona State University

Arkansas Children's Hospital Research Institute

Arkansas Heart Hospital

Arthritis & Rheumatic Disease Specialties Research

ArthroCare Corporation

Aspire Foundation

Aspirus Heart and Vascular Institute-Research and Education

Associated Scientists to Help Minimize Allergies

Atlanta Research and Education Foundation

Atlantic Health System

Auerbach Hematology Oncology Associates P C

Aultman Health Foundation

Aurora BayCare Medical Center

Aurora Health Care

Austin Speech Labs

Avera McKennan Hospital & University Health Center

Azusa Pacific University

Banner Health

Baptist Health South Florida

Barbara Ann Karmanos Cancer Institute

Barnes Retina Institute

Barrow Neurological Institute

Bastyr University

Bay Area Retina Associates

Baylor Breast Care Center

Baylor College of Medicine

Baylor Research Institute

Baylor University

Baystate Medical Center

Benaroya Research Institute

Benedictine University

Beth Israel Deaconess Medical Center

Beth Israel Medical Center

Binghamton University

Bio Products Laboratory

Biomedical Research Foundation

Biomedical Research Institute of New Mexico

Biosite

Blood Center of Wisconsin

Blood Systems Research Institute

Bloodworks (Puget Sound Blood Center)

Blythedale Children's Hospital

Boston Children's Hospital

Boston IVF

Boston Medical Center

Boston University

Bradley Hospital

Brain Sentinel

Brandeis University

Brentwood Biomedical Research Institute

Brigham and Women's Hospital

Broward Health

Brown University

BTG International Inc.

Bucci Laser Vision Institute

Butler Hospital

California Collaborative Treatment Group

California Medical Clinic for Headache

California Pacific Medical Center Research Institute

California Retina Consultants

Callender Center for Clinical Research

Cambridge Health Alliance

CAMC Health System

Cancer Center of Irvine

Cancer Research Network

Cardiopulmonary Research Science and Technology Institute

Cardiovascular Institute of the South Clinical Research Corporation

Care Management Plus

Carilion Clinic

Carnegie Mellon University

Carolinas Healthcare System

Carrick Institute for Graduate Studies

Case Comprehensive Cancer Center

Case Western Reserve University

Catholic Health Initiatives

Cedars Sinai Medical Center

Center for Human Reproduction

Center for Innovative Public Health Research

Center for Medicinal Cannabis Research

Center for Psychological Consultation

Central DuPage Hospital

Charles Drew University of Medicine and Science

Chattanooga-Hamilton County Hospital Authority

Chestnut Health Systems

Chicago Anesthesia Pain Specialists

Child Psychopharmacology Institute

Children's Healthcare of Atlanta

Children's Hospital & Research Center Oakland

Children's Hospital Los Angeles

Children's Hospital Medical Center, Cincinnati

Children's Hospital of Philadelphia

Children's Hospital of Pittsburgh

Children's Hospitals and Clinics of Minnesota

Children's Mercy Hospitals and Clinics

Children's Research Institute

Christiana Care Health Services

Christine M. Kleinert Institute for Hand and Microsurgery

Cincinnati Sportsmedicine Research and Education Foundation

City of Hope Medical Center

Claremont Graduate University

Clemson University

Cleveland Chiropractic College

Cleveland Clinic Florida

Cleveland Clinic Foundation

Clinvest

Coastal Carolina Radiation Oncology

Coastal Orthopedics & Sports Medicine

Colorado Joint Replacement

Colorado Prevention Center

Colorado State University

Columbia University

Community Research Initiative of New England

Comprehensive Cancer Center of Wake Forest University

Connecticut Children's Medical Center

CONRAD

Cook Children's Health Care System

COPD Foundation

Coram Clinical Trials

Cornea and Laser Eye Institute

Cornea Research Foundation of America

Cornell University

CPL Associates

Craig Hospital

Creighton University

Cure CMD

CyberKnife Centers of San Diego

Dana-Farber Cancer Institute

Danbury Hospital

Dartmouth-Hitchcock Medical Center

Dayton Children's Hospital

Defense and Veterans Center for Integrative Pain Management

DeNova Research

Dent Neuroscience Research Center

Denver Health and Hospital Authority

Des Moines University

Diamond Headache Clinic

Digestive & Liver Disease Specialists

Discover Vision Centers

Drexel University

Duke University Medical Center

Duquesne University

Durrie Vision

East Bay Institute for Research and Education

East Carolina University

East Tennessee State University

Eastern Maine Medical Center

Eastern Regional Medical Center

Eastern Virginia Medical School

Edward Hospital

Elman Retina Group

Elusys Therapeutics

Emory University

Englewood Hospital and Medical Center

Essentia Health

EvergreenHealth

Facet Technologies

Fenway Community Health

Fetal Medicine Foundation

FHI 360

Find A Cure Panel

First Affiliated Hospital of Harbin Medical University

Florida Academic Dermatology Centers

Florida Atlantic University

Florida Hospital

Florida Hospital Tampa Bay Division

Florida International University

Florida State University

Fondren Orthopedic Group L.L.P.

Fordham University

Foundation Fighting Blindness Clinical Research Institute

Foundation for Maternal Infant and Lactation Knowledge

Foundation for Southwest Orthopedic Research

Foundation for the National Institutes of Health

Fox Chase Cancer Center

Fred Hutchinson Cancer Research Center

Garden State Cancer Center at the Center for Molecular Medicine and Immunology

Geisinger Clinic

Genetic Disease Investigators

George Mason University

George Washington University

Georgetown University Medical Center

Georgia Institute of Technology

Georgia State University

Geriatric Oncology Consortium

Gillette Children's Specialty Healthcare

Glaucoma Associates of New York

Glaucoma Research & Education Group

Global Alliance for TB Drug Development

GRADE Study Group

Gramercy Research Group

Greater Houston Retina Research

Greenville Health System

Greenwich Hospital

Greenwood Genetic Center

Griffin Hospital

Group Health Cooperative

Gynecologic Oncology Associates

Gynuity Health Projects

H. Lee Moffitt Cancer Center & Research Institute

Hackensack University Medical Center

Hartford Hospital

Harvard Clinical Research Institute

Harvard Medical School

Harvard Pilgrim Health Care

Harvard School of Public Health

Harvard University

Hawaii Pacific Health

Health Decisions

Health Outcomes Solutions

HealthEast Care System

HealthPartners Institute

Heekin Orthopedic Research Institute

Henry Ford Health System

Henry M. Jackson Foundation for the Advancement of Military Medicine

Hepatitis Resource Network

High Point University

Hoag Memorial Hospital Presbyterian

Hoosier Cancer Research Network

HopeLab Foundation

Horizons International Peripheral Group

Hospital for Special Surgery, New York

Howard University

Hugo W. Moser Research Institute at Kennedy Krieger, Inc.

Huntington Medical Research Institutes

Huntington Memorial Hospital

i4Health

Icahn School of Medicine at Mount Sinai

IDRI

Illinois Retina Associates

INC Research

Indiana Hand to Shoulder Center

Indiana Kidney Stone Institute

Indiana University

Innovation Research & Training

Inova Health Care Services

Institute for Neurodegenerative Disorders

Integrative Medicine Institute

Intermountain Health Care, Inc.

International Clinical Research Institute

International Food Policy Research Institute

International Spine Study Group Foundation

iX Biopharma Ltd.

Jaeb Center for Health Research

James Graham Brown Cancer Center

James Madison University

JHSPH Center for Clinical Trials

John H. Stroger Hospital

John Wayne Cancer Institute

Johns Hopkins All Children's Hospital

Johns Hopkins Bloomberg School of Public Health

Johns Hopkins School of Medicine

Jonsson Comprehensive Cancer Center

Joslin Diabetes Center

Justice Resource Institute

Justin Parker Neurological Institute

Kaiser Permanente

Kentuckiana Cancer Institute

Kessler Foundation

Kettering Health Network

Kronos Longevity Research Institute

Lahey Clinic

Lancaster General Hospital

Landon Pediatric Foundation

Larkin Community Hospital

Laser Surgery Care

Legacy Biomechanics Laboratory

Legacy Health System

Lehigh University

Lehigh Valley Hospital

Leo W. Jenkins Cancer Center

Life Extension Foundation Inc.

Life Recovery Systems

Life University

LifeBridge Health

Lindner Center of HOPE

Logan College of Chiropractic

Loma Linda University

Long Island Vitreoretinal Consultants

Los Angeles Biomedical Research Institute

Louisiana State University Health Sciences Center in New Orleans

Louisiana State University Health Sciences Center Shreveport

Louisville Metabolic and Atherosclerosis Research Center

Loyola University

Ludwig Institute for Cancer Research

Maimonides Medical Center

Main Line Fertility Center

Maine Medical Center

Major Extremity Trauma Research Consortium

Manhattan Psychiatric Center

Maricopa Integrated Health System

Marquette University

Mary Crowley Medical Research Center

Marywood University

Masonic Cancer Center, University of Minnesota

Massachusetts Eye & Ear Infirmary

Massachusetts General Hospital

MassBiologics

Mayo Clinic

McGuire Research Institute

McLaren Health Care

McLean Hospital

McMaster University

MDRC

Medical College of Wisconsin

Medical University of South Carolina

Medicines for Malaria Venture

Mednax Center for Research, Education and Quality

Medstar Health Research Institute

Meharry Medical College

Memorial Health University Medical Center

Memorial Hermann Health System

Memorial Hospital of Rhode Island

Memorial Medical Center

Memorial Sloan Kettering Cancer Center

MemorialCare Health System

Men's Health Boston

Mercy Medical Center

MetroHealth Medical Center

mHealth UCSD

Michael J. Fox Foundation for Parkinson's Research

Michigan State University

Mid Atlantic Retina

Midwest Biomedical Research Foundation

Midwest Cardiovascular Research Foundation

Midwest Heart Foundation

Midwestern Regional Medical Center

Miller Orthopedic Specialists

Minneapolis Heart Institute Foundation

Minneapolis Medical Research Foundation

Molecular NeuroImaging

Monarch Medical Research

Monell Chemical Senses Center

Montefiore Medical Center

Mosaic Life Care

Mount Sinai Rehabilitation Hospital

Mountain Diabetes and Endocrine Center

Mountain States Tumor and Medical Research Institute

Moy-Fincher Medical Group

MultiCare Health System Research Institute

Multidisciplinary Association for Psychedelic Studies

Multiple Sclerosis Center of Northeastern New York

Musculoskeletal Transplant Foundation

Nambudripad's Allergy Research Foundation

Nathan Kline Institute for Psychiatric Research

National College of Natural Medicine

National Foundation for Fertility Research

National Jewish Health

National Pediatric Myoclonus Center

National University of Health Sciences

Nationwide Children's Hospital

Natural Immune Systems Inc

Nemours Children's Clinic

Neuronetics

Nevada Cancer Institute

New Approaches to Neuroblastoma Therapy Consortium

New England Research Institutes

New England Retina Associates

New Lexington Clinic

New Mexico Cancer Care Alliance

New York Blood Center

New York CFS Association

New York City Health and Hospitals Corporation

New York Glaucoma Research Institute

New York Institute of Technology

New York Medical College

New York Methodist Hospital

New York State Psychiatric Institute

New York University

New York University School of Medicine

Newton-Wellesley Hospital

Nicklaus Children's Hospital f/k/a Miami Children's Hospital

NJ Retina

North Dakota State University

North Florida Foundation for Research and Education

Northeast Regional Epilepsy Group

Northeastern University

Northern California Melanoma Center

Northern California Retina Vitreous Associates

NorthShore University HealthSystem

NorthShore University HealthSystem Research Institute

Northwell Health

Northwestern Health Sciences University

Northwestern University

Norton Healthcare

Norton Leatherman Spine Center

Nova Southeastern University

NRG Oncology

NxStage Medical

O & O Alpan LLC

OAD Orthopaedics

Oakland University

Ochsner Health System

Ohio State University Comprehensive Cancer Center

Ohio University

OhioHealth

OHSU Knight Cancer Institute

Oklahoma Medical Research Foundation

Oklahoma State University

Oklahoma State University Center for Health Sciences

Olive View-UCLA Education & Research Institute

Olmsted Medical Center

Ophthalmic Consultants of Boston

Ophthalmic Consultants of Long Island

Oregon Health & Science University

Oregon Research Institute

Oregon Social Learning Center

Oregon State University

OrthoCarolina Research Institute, Inc.

OrthoGeorgia

Orthopaedic Research Foundation

OSF Healthcare System

Ovation Fertility

Pachyonychia Congenita Project

Pacific Eye Associates

Pacific Health Foundation

Pacific Institute for Research and Evaluation

Pacific University

Pain Management Center of Paducah

Palmer College of Chiropractic

Palo Alto Medical Foundation

Palo Alto Veterans Institute for Research

Pancreatic Cancer Research Team

PATH

Pediatric Nephrology of Alabama

Pelvic and Sexual Health Institute

Penn State Milton S. Hershey Medical Center

Penn State University

Pennington Biomedical Research Center

Pepperdine University

Pharmaceutical Research Network

Pharmacology Research Institute

Philadelphia Eye Associates

Phoenix Children's Hospital

Physicians Committee for Responsible Medicine

Piedmont Healthcare

Planned Parenthood Federation of America

Planned Parenthood League of Massachusetts

PMRC

Population Council

Poudre Valley Health System

Practitioners Alliance Network

Pro-Change Behavior Systems

Prolong Pharmaceuticals

ProMedica Health System

Prostate Cancer Foundation of Chicago

Providence Health & Services

Provident Clinical Research

Public Health Management Corporation

Purdue University

Queen's Medical Centre

Quietmind Foundation

Radiant Research

RAND

Regional Obstetrical Consultants

Regis University

Rehabilitation Institute of Chicago

Renal Research Institute

Reproductive Medicine Associates of New Jersey

Retina Associates of Kentucky

Retina Foundation of the Southwest

Retina Institute of Hawaii

Retina Macula Institute

Retina Research Foundation

Retina Vitreous Associates of Florida

Retinal Consultants of Arizona

Rhode Island Hospital

Riverside County Regional Medical Center

RML Specialty Hospital

Robert Wood Johnson Foundation

Rochester Center for Behavioral Medicine

Rochester General Hospital

Rockefeller University

Rocky Mountain Cancer Centers

Roger Williams Medical Center

Roswell Park Cancer Institute

Rothman Institute Orthopaedics

Rowan University

RTI International

Rush University Medical Center

Rutgers, The State University of New Jersey

Sadick Research Group

Saint Elizabeth Regional Medical Center

Saint Francis Care

Saint Joseph Mercy Health System

Saint Louis University

Saint Luke's Health System

Saint Thomas Health Services

Samueli Institute for Information Biology

San Diego State University

Sanford Health

Sanford Research

Santa Barbara Cottage Hospital

Santa Clara Valley Health & Hospital System

Sarcoma Alliance for Research through Collaboration

Scott and White Hospital & Clinic

SCRI Development Innovations, LLC

Scripps Center for Integrative Medicine

Scripps Health

Scripps Translational Science Institute

Scripps Whittier Diabetes Institute

Seattle Biomedical Research Institute

Seattle Children's Hospital

Seattle Institute for Biomedical and Clinical Research

Seton Healthcare Family

Shady Grove Fertility Reproductive Science Center

Shafran Gastroenterology Center

Sharp HealthCare

Shriners Hospitals for Children

Sidney Kimmel Comprehensive Cancer Center

Skidmore College

Skin Care and Laser Physicians of Beverly Hills

Soul Medicine Institute

South Dakota State University

Southeast Renal Research Institute

Southeastern Gynecologic Oncology

Southern California College of Optometry

Southern California Institute for Research and Education

Southern California University of Health Sciences

Southern Illinois University

Southern Illinois University Carbondale

Southern Methodist University

Southwest Regional Wound Care Center

Southwestern Regional Medical Center

Spaulding Rehabilitation Hospital

SpectraScience

Spectrum Health Hospitals

SpinalMotion

Spokane Joint Replacement Center

Sport and Spine Rehab Clinical Research Foundation

Sprim Advanced Life Sciences

St. Barnabas Medical Center

St. Joseph Hospital of Orange

St. Joseph's Hospital and Medical Center, Phoenix

St. Jude Children's Research Hospital

St. Louis Children's Hospital

St. Luke's-Roosevelt Hospital Center

St. Vincent Carmel Hospital

St. Vincent's Medical Center

Stamford Hospital

Stanford University

State University of New York - Downstate Medical Center

State University of New York at Buffalo

State University of New York College of Optometry

Stony Brook University

Summa Health System

Sunnybrook Health Sciences Centre

SUNY Upstate Medical University

Swanson Center

Swedish Medical Center

Synergy Health Solutions

Syracuse University

Tampa General Hospital

Temple University

Tennessee Retina

Texas A&M University

Texas Back Institute

Texas Cardiac Arrhythmia Research Foundation

Texas Christian University

Texas Health Resources

Texas Heart Institute

Texas Retina Associates

Texas Scottish Rite Hospital for Children

Texas Tech

Texas Tech University Health Sciences Center

Texas Vascular Associates

Texas Woman's University

The Advanced Gynecologic Surgery Institute

The Alfred E. Mann Foundation for Scientific Research

The Brooklyn Hospital Center

The Center for Autism and Related Disorders

The Children's Health Council

The Cooper Health System

The Cooper Institute

The Epilepsy Study Consortium

The Forsyth Institute

The George Washington University Biostatistics Center

The Guthrie Clinic

The Hawkins Foundation

The Maas Clinic

The Medical Research Network

The Methodist Hospital System

The Mind Research Network

The Miriam Hospital

The National Center on Addiction and Substance Abuse at Columbia University

The New England Baptist Hospital

The New York Academy of Medicine

The New York Eye & Ear Infirmary

The New York Eye Cancer Center

The Ohio State University

The Reading Hospital and Medical Center

The Rogosin Institute

The Scripps Research Institute

The University of Akron

The University of Chicago

The University of Texas at Arlington

The University of Texas at Dallas

The University of Texas Health Science Center at San Antonio

The University of Texas Health Science Center at Tyler

The University of Texas Health Science Center, Houston

The University of Texas M. D. Anderson Cancer Center

The University of Texas, Galveston

The Western Pennsylvania Hospital

Therapeutic Advances in Childhood Leukemia Consortium

Therapeutic Concepts

Thomas Jefferson University

Tower Outpatient Surgical Center

Towson University

Translational Drug Development

Translational Oncology Research International

Translational Research in Oncology

Treatment Research Institute

TriHealth Inc.

Truman Medical Center

Truth Initiative

Tufts Medical Center

Tufts University

Tufts University School of Dental Medicine

Tulane University

Tulane University Health Sciences Center

Tulane University School of Medicine

Tuscaloosa Research & Education Advancement Corporation

U.S. Wound Registry

UNC Lineberger Comprehensive Cancer Center

UNC Nutrition Research Institute

University at Albany

University at Buffalo

University of Alabama at Birmingham

University of Arizona

University of Arkansas

University of Bridgeport

University of California, Berkeley

University of California, Davis

University of California, Irvine

University of California, Los Angeles

University of California, San Diego

University of California, San Francisco

University of Central Florida

University of Cincinnati

University of Colorado at Denver and Health Sciences Center

University of Connecticut

University of Connecticut Health Center

University of Delaware

University of Florida

University of Georgia

University of Hawaii

University of Houston

University of Illinois at Chicago

University of Illinois at Urbana-Champaign

University of Indianapolis

University of Iowa

University of Kansas Medical Center

University of Kentucky

University of Louisville

University of Maryland

University of Massachusetts

University of Memphis

University of Miami

University of Miami Sylvester Comprehensive Cancer Center

University of Michigan

University of Michigan Cancer Center

University of Minnesota - Clinical and Translational Science Institute

University of Mississippi Medical Center

University of Missouri-Columbia

University of Missouri-Kansas City

University of Montana

University of Nebraska Lincoln

University of Nebraska Medical Center

University of New Mexico

University of North Carolina, Chapel Hill

University of North Texas Health Science Center

University of Oklahoma

University of Oregon

University of Pennsylvania

University of Pittsburgh

University of Rhode Island

University of Rochester

University of South Alabama

University of South Carolina

University of South Florida

University of Southern California

University of Tennessee

University of Tennessee Cancer Institute

University of Texas

University of Texas at Austin

University of Texas Southwestern Medical Center

University of the Pacific

University of the Sciences in Philadelphia

University of Toledo Health Science Campus

University of Tulsa

University of Utah

University of Vermont

University of Virginia

University of Washington

University of Wisconsin, Madison

University Reproductive Associates

Urology of Virginia

Valley Anesthesiology Consultants

Valley Health System

Valley Retina Institute

Vanderbilt University

Vanderbilt-Ingram Cancer Center

Varian Medical

Vector Oncology

Veeda Oncology

Veterans Medical Research Foundation

Virginia Center for Reproductive Medicine

Virginia Commonwealth University

Virginia Polytechnic Institute and State University

Vision Specialists of Birmingham

Visiting Nurse Service of New York

Vitreous -Retina- Macula Consultants of New York

VIVA Physicians

Wake Forest Baptist Health

Wake Forest NCORP Research Base

Wake Forest School of Medicine

Washington Hospital Center

Washington State University

Washington University

Washington University Early Recognition Center

Wayne State University

Weill Medical College of Cornell University

WellSpan Health

West Coast Fertility Centers

West Penn Allegheny Health System

West Virginia University

Westat

Western Michigan University

Western Oregon University

Western Regional Medical Center

Western University of Health Sciences

William Beaumont Hospitals

William Marsh Rice University

William Sansum Diabetes Center

Wills Eye

Windber Research Institute

Winthrop University Hospital

Woman's

Women & Infants Hospital of Rhode Island

Wyss Institute at Harvard University

Yale University

Youngstown State University

Zipper Urogynecology Associates
