# Supplementary material for: Clinical trial registration and reporting: a survey of academic organizations in the United States
Source: BMC Med. 2018 May 2;16:60. doi: 10.1186/s12916-018-1042-6 (PMC5930804; doi:10.1186/s12916-018-1042-6)
Supplement: Supplementary file 3 — Participating accounts. (DOCX 476 kb) [file 12916_2018_1042_MOESM3_ESM.docx]

**ONLINE SUPPLEMENTS**

**Additional file 3: Participating accounts (N=366)**

| **Account name** | **RECORDS** | **CTSA** | **CANCER CENTER** | **MEDICAL SCHOOL** |
| --- | --- | --- | --- | --- |
| A.T. Still University of Health Sciences | 10 | NO | NO | YES |
| Abdul Latif Jameel Poverty Action Lab | 2 | NO | NO | NO |
| Abramson Cancer Center of the University of Pennsylvania | 226 | YES | YES | NO |
| Accelerated Community Oncology Research Network | 16 | NO | NO | NO |
| Accelerated Cure Project for Multiple Sclerosis | 2 | NO | NO | NO |
| Advocate Health Care | 17 | NO | NO | NO |
| Akron Children's Hospital | 24 | NO | NO | NO |
| Akron General Medical Center | 2 | NO | YES | NO |
| Albany College of Pharmacy and Health Sciences | 8 | NO | NO | NO |
| Alliance for Clinical Trials in Oncology | 299 | NO | NO | NO |
| Allina Health System | 14 | NO | NO | NO |
| American Academy of Pediatrics | 3 | NO | NO | NO |
| American Lung Association Asthma Clinical Research Centers | 3 | YES | NO | NO |
| Ann & Robert H Lurie Children's Hospital of Chicago | 89 | YES | NO | NO |
| Anne Arundel Health System Research Institute | 5 | NO | NO | NO |
| Appalachian State University | 10 | YES | NO | NO |
| Arbor Research Collaborative for Health | 3 | NO | NO | NO |
| Arkansas Children's Hospital Research Institute | 56 | YES | NO | NO |
| Atlantic Health System | 27 | NO | NO | NO |
| Auerbach Hematology Oncology Associates P C | 3 | NO | NO | NO |
| Avera McKennan Hospital & University Health Center | 22 | NO | NO | NO |
| Banner Health | 9 | NO | NO | NO |
| Baptist Health South Florida | 11 | NO | NO | NO |
| Barrow Neurological Institute | 3 | NO | NO | NO |
| Baylor Breast Care Center | 21 | NO | NO | YES |
| Baylor College of Medicine | 391 | NO | NO | YES |
| Baylor Research Institute | 76 | NO | YES | NO |
| Baylor University | 6 | NO | NO | NO |
| Baystate Medical Center | 26 | YES | YES | YES |
| Beth Israel Deaconess Medical Center | 221 | YES | NO | NO |
| Bio Products Laboratory | 2 | NO | NO | NO |
| Biosite | 15 | NO | NO | NO |
| Bloodworks (Puget Sound Blood Center) | 4 | YES | NO | NO |
| Blythedale Children's Hospital | 3 | NO | NO | NO |
| Boston Children's Hospital | 257 | NO | NO | NO |
| Boston IVF | 9 | NO | NO | NO |
| Boston Medical Center | 147 | YES | NO | NO |
| Boston University | 125 | YES | NO | YES |
| Brigham and Women's Hospital | 435 | YES | NO | NO |
| Brown University | 97 | NO | NO | YES |
| BTG International Inc. | 16 | NO | NO | NO |
| Butler Hospital | 60 | NO | NO | NO |
| California Retina Consultants | 8 | NO | NO | NO |
| Cambridge Health Alliance | 15 | NO | NO | NO |
| CAMC Health System | 27 | NO | YES | YES |
| Care Management Plus | 2 | YES | NO | YES |
| Carilion Clinic | 6 | NO | NO | YES |
| Case Comprehensive Cancer Center | 310 | YES | YES | YES |
| Cedars Sinai Medical Center | 186 | YES | YES | NO |
| Charles Drew University of Medicine and Science | 19 | YES | NO | NO |
| Chestnut Health Systems | 6 | NO | NO | NO |
| Chicago Anesthesia Pain Specialists | 5 | NO | NO | NO |
| Children's Healthcare of Atlanta | 79 | YES | NO | NO |
| Children's Hospital and Research Center Oakland | 39 | YES | NO | NO |
| Children's Hospital Los Angeles | 48 | NO | YES | YES |
| Children's Hospital Medical Center, Cincinnati | 256 | NO | NO | YES |
| Children's Hospital of Philadelphia | 216 | NO | YES | NO |
| Children's Research Institute | 79 | YES | NO | NO |
| Christiana Care Health Services | 59 | NO | NO | NO |
| Christine M. Kleinert Institute for Hand and Microsurgery | 7 | NO | NO | NO |
| Clemson University | 2 | NO | NO | NO |
| Cleveland Clinic Foundation | 329 | YES | NO | NO |
| Colorado State University | 14 | YES | NO | NO |
| Columbia University | 454 | YES | YES | YES |
| Community Research Initiative of New England | 6 | NO | NO | NO |
| Comprehensive Cancer Center of Wake Forest University | 160 | YES | YES | NO |
| Connecticut Children's Medical Center | 22 | NO | NO | NO |
| COPD Foundation | 3 | NO | NO | NO |
| Cornea Research Foundation of America | 4 | NO | NO | NO |
| CPL Associates | 9 | NO | NO | NO |
| Craig Hospital | 12 | NO | NO | NO |
| Creighton University | 94 | NO | NO | YES |
| Dana-Farber Cancer Institute | 481 | NO | YES | NO |
| Dartmouth-Hitchcock Medical Center | 223 | YES | YES | YES |
| Defense and Veterans Center for Integrative Pain Management | 10 | NO | NO | YES |
| Dent Neuroscience Research Center | 3 | NO | NO | NO |
| Des Moines University | 4 | NO | NO | YES |
| Duke University Medical Center | 1007 | YES | NO | YES |
| Duquesne University | 9 | NO | NO | NO |
| East Tennessee State University | 10 | NO | NO | YES |
| Englewood Hospital and Medical Center | 11 | NO | YES | NO |
| Essentia Health | 14 | NO | YES | NO |
| EvergreenHealth | 3 | NO | NO | NO |
| Facet Technologies | 2 | NO | NO | NO |
| First Affiliated Hospital of Harbin Medical University | 2 | NO | NO | NO |
| Florida Atlantic University | 18 | NO | NO | YES |
| Florida International University | 15 | NO | NO | YES |
| Fordham University | 2 | YES | NO | NO |
| Fox Chase Cancer Center | 112 | NO | YES | NO |
| Fred Hutchinson Cancer Research Center | 298 | YES | YES | NO |
| George Mason University | 13 | NO | NO | NO |
| Georgetown University Medical Center | 145 | YES | YES | YES |
| Georgia Institute of Technology | 2 | YES | NO | NO |
| Georgia State University | 6 | NO | NO | NO |
| Gillette Children's Specialty Healthcare | 6 | NO | NO | NO |
| Global Alliance for TB Drug Development | 5 | NO | NO | NO |
| GRADE Study Group | 2 | NO | NO | NO |
| Group Health Cooperative | 40 | NO | NO | NO |
| H. Lee Moffitt Cancer Center & Research Institute | 237 | NO | YES | NO |
| Hackensack University Medical Center | 34 | NO | YES | YES |
| Hartford Hospital | 49 | NO | NO | NO |
| Harvard Medical School | 7 | YES | NO | YES |
| Harvard Pilgrim Health Care | 12 | YES | NO | NO |
| Harvard School of Public Health | 13 | YES | NO | NO |
| Harvard University | 10 | NO | NO | NO |
| Hawaii Pacific Health | 8 | NO | NO | NO |
| HealthEast Care System | 5 | NO | NO | NO |
| Heekin Orthopedic Research Institute | 5 | NO | NO | NO |
| Henry M. Jackson Foundation for the Advancement of Military Medicine | 19 | NO | YES | YES |
| Hoag Memorial Hospital Presbyterian | 13 | NO | NO | NO |
| Hoosier Cancer Research Network | 52 | NO | YES | YES |
| HopeLab Foundation | 2 | NO | NO | NO |
| Hospital for Special Surgery, New York | 95 | YES | NO | NO |
| Hugo W. Moser Research Institute at Kennedy Krieger, Inc. | 39 | YES | NO | NO |
| i4Health | 5 | NO | NO | NO |
| Icahn School of Medicine at Mount Sinai | 341 | YES | YES | YES |
| IDRI | 5 | NO | NO | NO |
| Indiana University | 425 | YES | YES | YES |
| Inova Health Care Services | 19 | NO | NO | NO |
| Institute for Neurodegenerative Disorders | 38 | NO | NO | NO |
| Integrative Medicine Institute | 2 | NO | NO | NO |
| International Food Policy Research Institute | 3 | NO | NO | NO |
| International Spine Study Group Foundation | 2 | NO | NO | NO |
| iX Biopharma Ltd. | 2 | NO | NO | NO |
| Jaeb Center for Health Research | 32 | NO | NO | NO |
| John Wayne Cancer Institute | 7 | NO | YES | NO |
| Johns Hopkins All Children's Hospital | 6 | NO | NO | NO |
| Johns Hopkins Bloomberg School of Public Health | 67 | NO | NO | NO |
| Johns Hopkins School of Medicine | 678 | YES | NO | YES |
| Jonsson Comprehensive Cancer Center | 154 | NO | YES | NO |
| Joslin Diabetes Center | 38 | YES | NO | YES |
| Justin Parker Neurological Institute | 4 | NO | NO | NO |
| Kaiser Permanente | 118 | YES | YES | NO |
| Kettering Health Network | 9 | NO | NO | NO |
| Lahey Clinic | 41 | NO | NO | NO |
| Lancaster General Hospital | 8 | NO | NO | NO |
| Landon Pediatric Foundation | 6 | NO | NO | NO |
| Life Recovery Systems | 2 | YES | NO | NO |
| Life University | 3 | NO | NO | NO |
| LifeBridge Health | 13 | NO | NO | NO |
| Lindner Center of HOPE | 23 | NO | NO | YES |
| Loma Linda University | 106 | NO | YES | YES |
| Ludwig Institute for Cancer Research | 19 | NO | NO | NO |
| Maimonides Medical Center | 48 | NO | NO | NO |
| Maine Medical Center | 14 | NO | YES | NO |
| Major Extremity Trauma Research Consortium | 15 | NO | NO | NO |
| Mary Crowley Medical Research Center | 2 | NO | NO | NO |
| Marywood University | 3 | NO | NO | NO |
| Masonic Cancer Center, University of Minnesota | 172 | NO | YES | YES |
| Massachusetts Eye & Ear Infirmary | 50 | NO | NO | NO |
| Massachusetts General Hospital | 1176 | YES | NO | NO |
| MassBiologics | 3 | NO | NO | NO |
| Mayo Clinic | 1307 | YES | YES | YES |
| McLean Hospital | 95 | YES | NO | NO |
| MDRC | 7 | NO | NO | NO |
| Medical College of Wisconsin | 181 | YES | NO | YES |
| Medical University of South Carolina | 314 | YES | YES | YES |
| Medstar Health Research Institute | 48 | NO | YES | NO |
| Memorial Sloan Kettering Cancer Center | 1093 | NO | YES | NO |
| MetroHealth Medical Center | 31 | NO | NO | YES |
| Midwestern Regional Medical Center | 4 | NO | YES | NO |
| Minneapolis Heart Institute Foundation | 10 | NO | NO | NO |
| Minneapolis Medical Research Foundation | 36 | NO | NO | NO |
| Molecular NeuroImaging | 8 | NO | NO | NO |
| Monell Chemical Senses Center | 11 | NO | NO | NO |
| Mountain States Tumor and Medical Research Institute | 2 | NO | NO | NO |
| Multidisciplinary Association for Psychedelic Studies | 10 | NO | NO | NO |
| National Foundation for Fertility Research | 3 | NO | NO | NO |
| National Jewish Health | 51 | YES | YES | NO |
| Nationwide Children's Hospital | 187 | YES | NO | NO |
| Nemours Children's Clinic | 50 | YES | NO | NO |
| New England Retina Associates | 4 | NO | NO | NO |
| New Mexico Cancer Care Alliance | 59 | NO | YES | NO |
| New York City Health and Hospitals Corporation | 7 | NO | YES | NO |
| New York Medical College | 28 | YES | NO | YES |
| New York Methodist Hospital | 9 | NO | NO | NO |
| New York University School of Medicine | 439 | YES | YES | YES |
| Newton-Wellesley Hospital | 3 | YES | NO | NO |
| Nicklaus Children's Hospital f/k/a Miami Children's Hospital | 4 | NO | NO | NO |
| North Dakota State University | 5 | NO | NO | NO |
| North Florida Foundation for Research and Education | 6 | YES | NO | NO |
| NorthShore University HealthSystem | 13 | NO | NO | NO |
| Northwestern University | 644 | YES | YES | YES |
| Nova Southeastern University | 31 | NO | NO | YES |
| NRG Oncology | 3 | NO | NO | NO |
| Ohio State University Comprehensive Cancer Center | 187 | NO | YES | NO |
| Ohio University | 13 | NO | NO | YES |
| OhioHealth | 11 | NO | NO | NO |
| OHSU Knight Cancer Institute | 117 | YES | YES | NO |
| Oklahoma Medical Research Foundation | 10 | NO | NO | NO |
| Oklahoma State University | 5 | NO | NO | NO |
| Oklahoma State University Center for Health Sciences | 19 | NO | NO | YES |
| Olive View-UCLA Education & Research Institute | 5 | NO | NO | NO |
| Oregon Health & Science University | 335 | NO | NO | NO |
| Oregon Research Institute | 45 | NO | NO | NO |
| Oregon State University | 7 | NO | NO | NO |
| OrthoCarolina Research Institute, Inc. | 24 | NO | NO | NO |
| OSF Healthcare System | 11 | NO | NO | NO |
| Pacific Institute for Research and Evaluation | 4 | NO | NO | NO |
| Pacific University | 2 | NO | NO | NO |
| Palmer College of Chiropractic | 11 | NO | NO | NO |
| Palo Alto Veterans Institute for Research | 8 | YES | NO | NO |
| PATH | 7 | NO | NO | NO |
| Penn State Milton S. Hershey Medical Center | 156 | YES | YES | YES |
| Penn State University | 117 | NO | NO | NO |
| Pennington Biomedical Research Center | 134 | NO | NO | NO |
| Pepperdine University | 5 | NO | NO | NO |
| Planned Parenthood League of Massachusetts | 12 | NO | NO | NO |
| PMRC | 4 | NO | NO | NO |
| Population Council | 4 | NO | NO | NO |
| Poudre Valley Health System | 5 | NO | YES | NO |
| Practitioners Alliance Network | 2 | NO | NO | NO |
| Pro-Change Behavior Systems | 6 | NO | NO | NO |
| Providence Health & Services | 44 | NO | YES | NO |
| Provident Clinical Research | 4 | NO | NO | NO |
| Quietmind Foundation | 2 | NO | NO | NO |
| RAND | 37 | NO | NO | NO |
| Regis University | 3 | NO | NO | NO |
| Rehabilitation Institute of Chicago | 44 | NO | NO | NO |
| Retinal Consultants of Arizona | 4 | YES | NO | NO |
| Rockefeller University | 88 | YES | NO | NO |
| Roswell Park Cancer Institute | 212 | NO | YES | NO |
| Rutgers, The State University of New Jersey | 247 | NO | YES | YES |
| Sadick Research Group | 5 | NO | NO | NO |
| Saint Elizabeth Regional Medical Center | 5 | NO | NO | NO |
| Saint Louis University | 49 | NO | YES | YES |
| Saint Thomas Health Services | 2 | NO | YES | NO |
| Sarcoma Alliance for Research through Collaboration | 12 | NO | NO | NO |
| SCRI Development Innovations, LLC | 137 | NO | YES | NO |
| Scripps Translational Science Institute | 30 | YES | NO | NO |
| Seattle Children's Hospital | 84 | NO | NO | NO |
| Seattle Institute for Biomedical and Clinical Research | 26 | NO | NO | NO |
| Sharp HealthCare | 17 | NO | YES | NO |
| Sidney Kimmel Comprehensive Cancer Center | 322 | NO | YES | NO |
| Skidmore College | 4 | NO | NO | NO |
| Southern Illinois University | 23 | NO | NO | YES |
| Southern Illinois University Carbondale | 5 | NO | NO | NO |
| Southern Methodist University | 8 | NO | NO | NO |
| Spaulding Rehabilitation Hospital | 47 | YES | NO | YES |
| Spectrum Health Hospitals | 42 | NO | NO | NO |
| Sport and Spine Rehab Clinical Research Foundation | 7 | NO | NO | NO |
| St. Barnabas Medical Center | 4 | NO | NO | NO |
| St. Joseph Hospital of Orange | 5 | NO | NO | NO |
| St. Jude Children's Research Hospital | 228 | NO | YES | NO |
| St. Louis Children's Hospital | 2 | NO | NO | NO |
| St. Vincent Carmel Hospital | 3 | NO | NO | NO |
| Stanford University | 969 | YES | YES | YES |
| State University of New York - Downstate Medical Center | 12 | NO | NO | YES |
| State University of New York at Buffalo | 52 | YES | NO | YES |
| Stony Brook University | 68 | NO | YES | YES |
| Sunnybrook Health Sciences Centre | 2 | NO | NO | NO |
| SUNY Upstate Medical University | 102 | NO | YES | YES |
| Swedish Medical Center | 28 | NO | YES | NO |
| Temple University | 99 | NO | YES | YES |
| Texas A&M University | 33 | NO | NO | NO |
| Texas Back Institute | 3 | NO | NO | NO |
| Texas Heart Institute | 3 | NO | NO | NO |
| Texas Scottish Rite Hospital for Children | 4 | YES | NO | NO |
| Texas Tech | 8 | NO | NO | NO |
| Texas Tech University Health Sciences Center | 52 | NO | NO | NO |
| Texas Vascular Associates | 2 | NO | NO | NO |
| Texas Woman's University | 29 | NO | NO | NO |
| The Advanced Gynecologic Surgery Institute | 2 | NO | NO | NO |
| The Children's Health Council | 2 | NO | NO | NO |
| The Guthrie Clinic | 2 | NO | NO | NO |
| The Medical Research Network | 4 | NO | NO | NO |
| The Methodist Hospital System | 50 | NO | YES | NO |
| The Mind Research Network | 5 | NO | NO | NO |
| The National Center on Addiction and Substance Abuse at Columbia University | 4 | NO | NO | NO |
| The New York Eye & Ear Infirmary | 14 | YES | NO | NO |
| The Ohio State University | 279 | YES | YES | YES |
| The Rogosin Institute | 16 | YES | NO | NO |
| The University of Chicago | 445 | YES | YES | YES |
| The University of Texas at Arlington | 2 | YES | NO | NO |
| The University of Texas at Dallas | 6 | NO | NO | NO |
| The University of Texas Health Science Center at San Antonio | 184 | YES | YES | YES |
| The University of Texas Health Science Center, Houston | 305 | YES | NO | YES |
| The University of Texas M. D. Anderson Cancer Center | 1563 | YES | YES | NO |
| The University of Texas, Galveston | 84 | YES | YES | YES |
| The Western Pennsylvania Hospital | 2 | NO | NO | NO |
| Therapeutic Concepts | 4 | NO | NO | NO |
| Thomas Jefferson University | 208 | NO | YES | YES |
| Towson University | 2 | NO | NO | NO |
| Translational Drug Development | 4 | NO | NO | NO |
| Translational Oncology Research International | 10 | NO | YES | NO |
| Treatment Research Institute | 19 | NO | NO | NO |
| TriHealth Inc. | 43 | NO | NO | NO |
| Truth Initiative | 5 | NO | NO | NO |
| Tufts Medical Center | 83 | NO | YES | NO |
| Tufts University | 72 | YES | NO | YES |
| Tufts University School of Dental Medicine | 9 | NO | NO | NO |
| Tuscaloosa Research & Education Advancement Corporation | 16 | NO | NO | NO |
| U.S. Wound Registry | 5 | NO | NO | NO |
| University at Albany | 3 | NO | NO | NO |
| University of Alabama at Birmingham | 410 | YES | YES | YES |
| University of Arizona | 159 | NO | NO | YES |
| University of Arkansas | 205 | YES | NO | YES |
| University of California, Berkeley | 18 | NO | NO | NO |
| University of California, Davis | 315 | YES | NO | NO |
| University of California, Irvine | 263 | YES | YES | YES |
| University of California, Los Angeles | 434 | YES | NO | YES |
| University of California, San Francisco | 960 | YES | YES | YES |
| University of Cincinnati | 183 | YES | YES | YES |
| University of Colorado at Denver and Health Sciences Center | 491 | YES | YES | YES |
| University of Connecticut | 23 | NO | NO | NO |
| University of Connecticut Health Center | 128 | NO | NO | NO |
| University of Florida | 562 | YES | YES | YES |
| University of Georgia | 20 | NO | NO | NO |
| University of Hawaii | 43 | NO | YES | YES |
| University of Illinois at Chicago | 126 | YES | NO | YES |
| University of Illinois at Urbana-Champaign | 47 | NO | [Skipped] | NO |
| University of Indianapolis | 7 | NO | NO | NO |
| University of Iowa | 242 | YES | YES | YES |
| University of Kansas Medical Center | 250 | YES | YES | YES |
| University of Kentucky | 165 | YES | NO | NO |
| University of Louisville | 114 | NO | YES | YES |
| University of Maryland | 261 | NO | YES | YES |
| University of Massachusetts | 123 | YES | NO | YES |
| University of Miami | 307 | YES | YES | YES |
| University of Miami Sylvester Comprehensive Cancer Center | 32 | YES | YES | NO |
| University of Michigan | 588 | YES | NO | YES |
| University of Michigan Cancer Center | 167 | YES | YES | NO |
| University of Minnesota - Clinical and Translational Science Institute | 441 | YES | YES | YES |
| University of Mississippi Medical Center | 73 | YES | YES | YES |
| University of Missouri-Columbia | 122 | NO | NO | YES |
| University of Missouri-Kansas City | 21 | YES | NO | YES |
| University of Montana | 7 | NO | NO | NO |
| University of Nebraska Lincoln | 3 | NO | NO | NO |
| University of Nebraska Medical Center | 232 | NO | YES | YES |
| University of New Mexico | 125 | YES | NO | YES |
| University of North Carolina, Chapel Hill | 520 | YES | NO | YES |
| University of Oklahoma | 149 | NO | NO | YES |
| University of Pennsylvania | 630 | YES | NO | NO |
| University of Pittsburgh | 946 | YES | YES | YES |
| University of Rochester | 353 | YES | YES | YES |
| University of South Alabama | 12 | NO | NO | NO |
| University of South Florida | 168 | NO | NO | YES |
| University of Southern California | 292 | YES | YES | YES |
| University of Texas Southwestern Medical Center | 393 | YES | YES | YES |
| University of the Pacific | 2 | NO | NO | NO |
| University of Utah | 380 | YES | YES | YES |
| University of Virginia | 288 | NO | YES | YES |
| University of Washington | 530 | YES | YES | YES |
| University of Wisconsin, Madison | 482 | YES | YES | YES |
| University Reproductive Associates | 6 | NO | NO | NO |
| Urology of Virginia | 2 | NO | NO | NO |
| Valley Anesthesiology Consultants | 2 | NO | NO | NO |
| Valley Retina Institute | 3 | NO | NO | NO |
| Vanderbilt University | 594 | YES | NO | NO |
| Vector Oncology | 4 | NO | NO | NO |
| Virginia Commonwealth University | 243 | YES | YES | YES |
| Visiting Nurse Service of New York | 2 | NO | NO | NO |
| Wake Forest NCORP Research Base | 21 | YES | YES | YES |
| Washington State University | 13 | NO | NO | NO |
| Washington University | 743 | YES | YES | YES |
| Weill Medical College of Cornell University | 394 | YES | [Skipped] | NO |
| WellSpan Health | 7 | NO | NO | NO |
| Westat | 45 | NO | NO | NO |
| Western Michigan University | 2 | NO | NO | NO |
| Western Regional Medical Center | 19 | NO | YES | NO |
| William Beaumont Hospitals | 97 | NO | NO | NO |
| William Marsh Rice University | 3 | NO | NO | NO |
| William Sansum Diabetes Center | 13 | NO | NO | NO |
| Windber Research Institute | 2 | NO | NO | NO |
| Women & Infants Hospital of Rhode Island | 38 | NO | NO | NO |
| Yale University | 694 | YES | YES | YES |

**ACCOUNT NAME:** Name of the PRS account that participated in the survey. **RECORDS:** Number of studies registered on ClnicalTrial.gov in August 2016 for which the organization was listed as the “lead sponsor” and the study was conducted in the U.S. **CTSA:** Whether the account is affiliated Clinical and Translational Science Awards (CTSAs). **CANCER CENTERS:** Whether the account is affiliated with a cancer center (NCI designated and not NCI designated). **MEDICAL SCHOOLS:** Whether the account is affiliated with a medical school.
