# Supplementary material for: Clinical trial registration and reporting: a survey of academic organizations in the United States
Source: BMC Med. 2018 May 2;16:60. doi: 10.1186/s12916-018-1042-6 (PMC5930804; doi:10.1186/s12916-018-1042-6)
Supplement: Supplementary file 4 — Additional survey results. (DOCX 440 kb) [file 12916_2018_1042_MOESM4_ESM.docx]

**ONLINE SUPPLEMENTS**

**Additional file 4: Additional survey results**

| **Question (number of participants who viewed question)** | **No** | **%** |
| --- | --- | --- |
| **According to the policy, who may register a trial? (N=139) ^1^** |  |  |
| Principal investigator | 102 | 73% |
| Designee of the principal investigator | 82 | 59% |
| Institutional review board | 4 | 3% |
| PRS administrator | 48 | 35% |
| Other | 6 | 4% |
| This responsibility is not assigned in the policy | 11 | 8% |
| Don't know | 2 | 1% |
| Skipped (did not answer) | 1 | 1% |
| According to the policy, who is responsible for determining if trials must be registered? (N=139)^1^ |  |  |
| Principal investigator | 84 | 60% |
| Institutional review board | 28 | 20% |
| PRS administrator | 40 | 29% |
| Other | 16 | 12% |
| This responsibility is not assigned in the policy | 18 | 13% |
| Don't know | 1 | 1% |
| Skipped (did not answer) | 1 | 1% |
| According to the policy, what penalties may be applied by the organization to an investigator for non-compliance with the registration policy? (N=27)^2,3^ |  |  |
| May not continue with enrollment on current study | 10 | 37% |
| May not begin any new research projects | 8 | 30% |
| May be suspended from work | 2 | 7% |
| Investigator or their department may be assessed a monetary penalty | 11 | 41% |
| Other (please specify) | 7 | 26% |
| Don't know | 5 | 19% |
| Skipped (did not answer) | 0 | 0% |
| For accounts with policies that do not indicate who is responsible for registering trials: In practice, who is responsible for determining if trials must be registered? (N=18)^2,4^ |  |  |
| Principal investigator | 9 | 50% |
| Institutional review board | 3 | 17% |
| PRS administrator | 7 | 39% |
| Other | 9 | 50% |
| Don't know | 1 | 6% |
| Skipped (did not answer) | 0 | 0% |
| For accounts with policies that do not specify a person who is responsible for registering trials: In practice, who is responsible for registering trials? (N=11)^2,5^ |  |  |
| Principal investigator | 9 | 82% |
| Designee of the principal investigator | 8 | 73% |
| Institutional review board | 0 | 0% |
| PRS administrator | 4 | 36% |
| Other | 1 | 9% |
| Don't know | 0 | 0% |
| Skipped (did not answer) | 0 | 0% |
| Which trials are registered according to the policy: All trials (N=140)^1^ |  |  |
| Required to register | 46 | 33% |
| Encouraged but not required to register | 49 | 35% |
| Not mentioned in the policy | 39 | 28% |
| Skipped (did not answer) | 6 | 4% |
| Which trials are registered according to the policy: Trials reimbursed by CMS (N=140)^1^ |  |  |
| Required to register | 61 | 44% |
| Encouraged but not required to register | 16 | 11% |
| Not mentioned in the policy | 55 | 39% |
| Skipped (did not answer) | 8 | 6% |
| Which trials are registered according to the policy: Trials meeting ICMJE definition (N=140)^1^ |  |  |
| Required to register | 57 | 41% |
| Encouraged but not required to register | 46 | 33% |
| Not mentioned in the policy | 29 | 21% |
| Skipped (did not answer) | 8 | 6% |
| **Which trials are registered according to the policy: Trials applicable under FDAAA (N=140)**^1^ |  |  |
| Required to register | 118 | 84% |
| Encouraged but not required to register | 5 | 4% |
| Not mentioned in the policy | 11 | 8% |
| Skipped (did not answer) | 6 | 4% |
| Which trials are registered according to the policy: Trials funded by NIH (N=140)^1^ |  |  |
| Required to register | 72 | 51% |
| Encouraged but not required to register | 22 | 16% |
| Not mentioned in the policy | 40 | 29% |
| Skipped (did not answer) | 6 | 4% |
| Which trials are registered according to the policy: Trials funded by NCI (N=140)^1^ |  |  |
| Required to register | 53 | 38% |
| Encouraged but not required to register | 19 | 14% |
| Not mentioned in the policy | 62 | 44% |
| Skipped (did not answer) | 6 | 4% |
| Which trials are registered according to the policy: Other (N=140)^1^ |  |  |
| Required to register | 9 | 6% |
| Encouraged but not required to register | 9 | 6% |
| Not mentioned in the policy | 92 | 66% |
| Skipped (did not answer) | 30 | 21% |
| Who is assigned the role of “Responsible party”: Always designated according to policy (N=61)^6^ |  |  |
| Principal investigator | 39 | 64% |
| Sponsor | 19 | 31% |
| Don’t know | 3 | 5% |
| Skipped (did not answer) | 0 | 0% |
| Who is assigned the role of “Responsible party”: May be designated according to policy (N=68)^2,6^ |  |  |
| Principal investigator | 46 | 67% |
| Sponsor | 42 | 61% |
| Other | 0 | 0% |
| This responsibility is not assigned in the policy | 16 | 24% |
| Don’t know | 2 | 3% |
| Skipped (did not answer) | 0 | 0% |
| Who is assigned the role of “Responsible party”: Designated in practice (N=16)^2,7^ |  |  |
| Principal investigator | 12 | 75% |
| Sponsor | 8 | 50% |
| Don’t know | 1 | 6% |
| Skipped (did not answer) | 0 | 0% |
| Which trials are registered in practice: All trials (N=157)^8^ |  |  |
| Always registered | 77 | 49% |
| Sometimes registered | 61 | 39% |
| Not registered | 8 | 5% |
| Skipped (did not answer) | 11 | 1% |
| Which trials are registered in practice: Trials reimbursed by CMS (N=157)^8^ |  |  |
| Always registered | 75 | 48% |
| Sometimes registered | 32 | 20% |
| Not registered | 16 | 10% |
| Skipped (did not answer) | 34 | 22% |
| Which trials are registered in practice: Trials meeting ICMJE definition (N=157)^8^ |  |  |
| Always registered | 80 | 51% |
| Sometimes registered | 38 | 24% |
| Not registered | 12 | 8% |
| Skipped (did not answer) | 27 | 17% |
| Which trials are registered in practice: Trials applicable under FDAAA (N=157)^8^ |  |  |
| Always registered | 112 | 71% |
| Sometimes registered | 12 | 8% |
| Not registered | 10 | 6% |
| Skipped (did not answer) | 23 | 15% |
| Which trials are registered in practice: Trials funded by NIH (N=157)^8^ |  |  |
| Always registered | 84 | 54% |
| Sometimes registered | 39 | 25% |
| Not registered | 9 | 6% |
| Skipped (did not answer) | 25 | 16% |
| Which trials are registered in practice: Trials funded by NCI (N=157)^8^ |  |  |
| Always registered | 79 | 50% |
| Sometimes registered | 27 | 17% |
| Not registered | 19 | 12% |
| Skipped (did not answer) | 32 | 20% |
| According to the organization policy, who is responsible for entering results? (n=115)^2,9^ |  |  |
| Principal investigator | 88 | 63% |
| PRS administrator | 24 | 17% |
| Other | 7 | 5% |
| This responsibility is not assigned in the policy | 12 | 9% |
| Don't know | 0 | 0% |
| Skipped (did not answer) | 0 | 0% |
| In practice, who is responsible for monitoring if results are entered on time? (N=8)^2,10^ |  |  |
| Principal investigator | 4 | 50% |
| Institutional review board | 0 | 0% |
| PRS administrator | 4 | 50% |
| Other | 2 | 25% |
| Don't know | 0 | 0% |
| Skipped (did not answer) | 0 | 0% |
| For accounts with a results reporting policy that does not assign responsibility for entering results: In practice, who is responsible for entering results? (N=12)^2,11^ |  |  |
| Principal investigator or their designee | 12 | 100% |
| PRS administrator | 4 | 33% |
| Other | 1 | 8% |
| Don't know | 0 | 0% |
| Skipped (did not answer) | 0 | 0% |
| For accounts that do not have a results reporting policy: In practice, who is responsible for entering results? (N=193)^2,12^ |  |  |
| Principal investigator | 145 | 75% |
| PRS administrator | 47 | 24% |
| Other | 14 | 7% |
| Don't know | 4 | 2% |
| Skipped (did not answer) | 0 | 0% |
| According to the policy, what penalties may be applied by the organization to an investigator for non-compliance with the results reporting policy? (N=27)^2,13^ |  |  |
| May not continue with enrollment on current study | 10 | 37% |
| May not begin any new research projects | 8 | 30% |
| May be suspended from work | 2 | 7% |
| Investigator or their department may be assessed a monetary penalty | 11 | 41% |
| Other (please specify) | 7 | 26% |
| Don't know | 3 | 11% |
| Skipped (did not answer) | 0 | 0% |
| In which office are staff who support trial registration and results reporting employed? (N=338)^2^ |  |  |
| Dedicated ClinicalTrials.gov office | 5 | 1% |
| Clinical Research | 89 | 26% |
| Clinical Trials | 36 | 11% |
| Institutional Review Board (IRB) | 49 | 14% |
| Quality Improvement | 17 | 5% |
| Regulatory Affairs | 42 | 12% |
| Research Administration | 86 | 25% |
| Research Compliance | 60 | 18% |
| Specific departments | 31 | 9% |
| Other | 50 | 15% |
| Don’t know | 8 | 2% |
| Skipped (did not answer) | 1 | <1% |
| Does the organization have a dedicated CT.gov program or office? (N=325) |  |  |
| Yes | 65 | 20% |
| No | 226 | 70% |
| Don’t know | 33 | 10% |
| Skipped question (did not answer) | 1 | <1% |
| Does the CTSA support compliance with trial registration and results reporting requirements (N=109)^14^ |  |  |
| Yes | 24 | 22% |
| No | 52 | 48% |
| Don’t know | 31 | 28% |
| Skipped (did not answer) | 2 | 2% |

^1^ The number of accounts that viewed each question is less than the total number of accounts in the study because (1) participants did not see all questions because of skip logic, and (2) some participants discontinued the survey before viewing all questions. The number of possible responses was limited to the accounts with a registration policy.

^2^ Because participants could “check all that apply”, the sum of all categories exceeds the number of participants who responded (i.e., some participants selected multiple responses).

^3^ The number of possible responses was limited to accounts with a policy that includes penalties for investigators who do not comply with the registration policy.

^4^ The number of possible responses was limited to accounts with policies that do not indicate who is responsible for registering trials.

^5^ The number of possible responses was limited to the accounts with policies that do not specify a person who is responsible for registering trials.

^6^ We asked the accounts with a registration policy whether a certain entity is always designated the responsible party. Based on their response, 61 accounts were asked to identify the entity that is always designated according to the policy, and 68 accounts were asked to identify entities that may be designated according to the policy.

^7^ The number of possible responses was limited to the 17 accounts with policies that do not specify a person or entity to designate as the responsible party.

^8^ The number of possible responses was limited to the accounts that do not have a registration policy.

^9^ The number of possible responses was limited to the accounts with a results reporting policy.

^10^ The number of possible responses was limited to the accounts with policies that do not specify a person who is responsible for monitoring if results are entered on time.

^11^ The number of possible responses was limited to the accounts with a results reporting policy that does not assign responsibility for entering results.

^12^ The number of possible responses was limited to the accounts that do not have a results reporting policy.

^13^ The number of possible responses was limited to the accounts that indicated an investigator could be penalized by the organization for noncompliance with the results reporting policy.

^14^ The number of possible responses was limited to the accounts affiliated with a CTSA.
