# Supplementary material for: Clinical trial registration and reporting: a survey of academic organizations in the United States
Source: BMC Med. 2018 May 2;16:60. doi: 10.1186/s12916-018-1042-6 (PMC5930804; doi:10.1186/s12916-018-1042-6)
Supplement: Supplementary file 5 — Sensitivity analysis. (DOCX 436 kb) [file 12916_2018_1042_MOESM5_ESM.docx]

**ONLINE SUPPLEMENTS**

**Additional file 5. Sensitivity analysis**

| **Question (number of participants who viewed question)** | **Before**  **Jan 18 2017**  **(N=335)** | | **on or after**  **Jan 18, 2017**  **(N=31)** | | **Total**  **(N=366)** | |
| --- | --- | --- | --- | --- | --- | --- |
| Number of records | 38,379  (mean=114, SD= 215) | | 1,972  (mean=64, SD =143) | | 40351  (mean=110, SD =210) | |
| Does the organization have a registration policy? (N=366) |  |  |  |  |  |  |
| Yes | 144 | 43% | 12 | 39% | 156 | 43% |
| No | 158 | 47% | 15 | 48% | 173 | 47% |
| Don’t know | 33 | 10% | 4 | 13% | 37 | 10% |
| Does the organization have a results reporting policy? (N=366) |  |  |  |  |  |  |
| Yes | 122 | 36% | 7 | 23% | 129 | 35% |
| No | 173 | 52% | 20 | 65% | 193 | 53% |
| Don’t know | 40 | 12% | 4 | 13% | 44 | 12% |
| Does the organization have an electronic system for managing trial registration or results reporting? (N=366) |  |  |  |  |  |  |
| Yes | 61 | 18% | 7 | 23% | 68 | 19% |
| No | 252 | 75% | 20 | 65% | 272 | 74% |
| Don’t know | 22 | 7% | 4 | 13% | 26 | 7% |
